# Implications of a New Obesity Definition Among the All of Us Cohort

**DOI:** 10.1001/jamanetworkopen.2025.37619

**Published:** 2025-10-15

**Authors:** Lindsay T. Fourman, Aya Awwad, Alba Gutiérrez-Sacristán, Camille A. Dash, Julia E. Johnson, Allison K. Thistle, Nikhita Chahal, Sara L. Stockman, Mabel Toribio, Chika Anekwe, Arijeet K. Gattu, Steven K. Grinspoon

**Affiliations:** 1 Metabolism Unit, Massachusetts General Hospital and Harvard Medical School, Boston; 2 Department of Biomedical Informatics, Harvard Medical School, Boston, Massachusetts

## Abstract

**Importance:**

A recent *Lancet* Commission proposed an obesity definition that integrates body mass index (BMI [calculated as weight in kilograms divided by height in meters squared]) with anthropometric measures, marking a paradigm shift in how obesity is conceptualized and classified.

**Objective:**

To determine the clinical implications of the new definition of obesity.

**Design, Setting, and Participants:**

This population-based longitudinal cohort study leveraged data from the All of Us cohort. Participants in All of Us with complete anthropometric data were enrolled between May 31, 2017, and September 30, 2023, with a median follow-up of 4.0 (IQR, 1.7-4.7) years.

**Exposure:**

Obesity classified by traditional and new definitions.

**Main Outcomes and Measures:**

Obesity was defined by the new framework using sex- and race-specific thresholds as (1) BMI above the traditional obesity threshold plus at least 1 elevated anthropometric measure or BMI greater than 40 (BMI-plus-anthropometric obesity) or (2) at least 2 elevated anthropometric measures with BMI below the traditional obesity threshold (anthropometric-only obesity). Obesity was categorized as clinical or preclinical based on organ dysfunction and/or physical limitation.

**Results:**

Among 301 026 individuals (183 633 [61.0%] female; median age, 54 [IQR, 38-65] years), 206 361 (68.6%) had obesity according to the new definition vs 128 992 (42.9%) according to the traditional definition, due to inclusion of individuals with anthropometric-only obesity. Among the overall cohort, 108 650 individuals (36.1%) had clinical obesity according to the new definition; this prevalence increased with age, as 24 498 of 45 018 individuals 70 years or older (54.4%) had clinical obesity. Compared with no obesity, odds ratios of organ dysfunction were 3.31 (95% CI, 3.24-3.37) for BMI-plus-anthropometric obesity and 1.76 (95% CI, 1.73-1.80) for anthropometric-only obesity per the new definition. In longitudinal analyses, clinical obesity conferred elevated risks of incident diabetes (adjusted hazard ratio [AHR], 6.11; 95% CI, 5.67-6.60), cardiovascular events (AHR, 5.88; 95% CI, 5.38-6.43), and all-cause mortality (AHR, 2.71; 95% CI, 2.41-3.05) compared with no obesity or organ dysfunction. Preclinical obesity was also associated with increased risks of incident diabetes (AHR, 3.32; 95% CI, 3.08-3.58) and cardiovascular events (AHR, 1.40; 95% CI, 1.27-1.55), albeit to a lesser degree.

**Conclusions and Relevance:**

In this cohort study, adoption of the new definition of obesity significantly increased obesity prevalence with major implications for clinical practice and public policy. The new framework effectively stratified individuals at high risk of organ dysfunction and long-term complications while introducing anthropometric-only obesity and preclinical obesity as distinct entities warranting further study.

## Key Points

**Question:**

What are the clinical implications of a definition of obesity that integrates body mass index (BMI) with anthropometric measures?

**Findings:**

In this analysis of the All of Us cohort that included 301 026 adults, the prevalence of obesity increased by 60% when using the new definition compared with the traditional BMI-based definition. This rise was driven by inclusion of individuals with anthropometric-only obesity (ie, elevated anthropometrics despite BMI below the traditional obesity threshold).

**Meaning:**

These findings suggest major implications for clinical practice and public policy while also introducing high-priority areas for further study, such as optimal prevention and management of anthropometric-only obesity.

## Teaser

This cohort study examines how the application of a new definition of obesity, which uses anthropometric measures and traditional body mass index measures, affects the prevalence of and outcomes associated with obesity in the All of Us cohort.

## Introduction

Obesity has traditionally been defined as elevated body mass index (BMI; calculated as weight in kilograms divided by height in square meters), yet BMI alone is an imprecise measure of adiposity. Recognizing this limitation, a recent consensus guideline published in *The Lancet Diabetes & Endocrinology* proposed a new definition of obesity that incorporates anthropometrics and/or direct measures of body fat to better differentiate adipose tissue excess.^1^ Developed by an international commission of experts spanning multiple specialties and countries, this guideline has already been endorsed by at least 76 professional organizations, marking a significant shift in how obesity will be conceptualized and classified.

The new definition allows for classification of obesity based on any of the following criteria: (1) elevated BMI plus at least 1 elevated anthropometric measure (eg, waist circumference, waist-to-hip ratio, and/or waist-to-height ratio) or BMI greater than 40; (2) at least 2 elevated anthropometric measures, irrespective of BMI; or (3) excess body fat as assessed by dual-energy x-ray absorptiometry or similar modalities. The guideline also introduces the concepts of clinical and preclinical obesity to differentiate individuals with vs without obesity-associated organ dysfunction and/or physical limitation. The consensus group suggests lower urgency and intensity of care for preclinical obesity, with pharmacologic and surgical interventions reserved for select cases. This approach represents a notable departure from current clinical practice, recent clinical trials, and US Food and Drug Administration (FDA)–approved use of modern antiobesity medications, which have traditionally considered all individuals with obesity to be candidates for therapy.^2,3,4,5,6,7^

The new obesity definition may have major ramifications for patients, clinicians, payers, and policymakers. Nonetheless, its clinical implications and relevance to long-term health outcomes have yet to be comprehensively evaluated in a large cohort. In this study, we applied the new obesity definition to the US-based All of Us (AoU) cohort with the following objectives: (1) to determine the prevalence of obesity and clinical obesity under the new definition, including variations by age, sex, and race; (2) to compare characteristics of individuals who meet criteria for obesity based on elevated BMI plus anthropometric measures vs elevated anthropometric measures alone; and (3) to evaluate longitudinal health outcomes across obesity subgroups compared with individuals without obesity. This analysis delineates the clinical and practical relevance of the new obesity definition and highlights areas for further investigation.

## Methods

### Study Participants

We leveraged the AoU cohort to examine the clinical implications of the new obesity guideline. All US adults able to provide consent were eligible to enroll in the AoU research program.^8,9^ We analyzed a controlled tier dataset (version 8; C2024Q3R4; February 3, 2025) for participants enrolled between May 31, 2017, and September 30, 2023 (median follow-up, 4.0 [IQR, 1.7-4.7 years). All participants provided written informed consent, and the institutional review board at Massachusetts General Hospital, Boston, approved this study. This study followed the Strengthening the Reporting of Observational Studies in Epidemiology (STROBE) reporting guideline.

We included individuals aged 18 to 80 years at baseline with complete physical measurements and electronic health record (EHR) data available (eMethods and eFigures 1 and 2 in Supplement 1). Characteristics were similar between participants included in this analysis and those excluded due to missing anthropometric data (eTable 1 in Supplement 1).

### Study Procedures

At baseline, participants underwent standardized measurements of height, weight, waist circumference, and hip circumference. Participants also completed health surveys, including demographic assessments (eg, self-reported sex, race and ethnicity), and could complete additional health surveys over time. Race was classified as American Indian or Alaska Native, Asian, Black or African American, Middle Eastern or North African, White, and other race (including Native Hawaiian or Other Pacific Islander, multiracial, did not identify with any group, and preferred not to answer); ethnicity was classified as Hispanic or Latinx or non–Hispanic or Latinx. These data were collected to compare obesity prevalence and phenotypes by race and to adjust for race in multivariable analyses. Longitudinal EHR data from all encounters before and after enrollment, including *International Classification of Diseases, Ninth Revision* (*ICD-9*), and *International and Statistical Classification of Diseases, Tenth Revision* (*ICD-10*), codes and clinical laboratory results, were abstracted from partnered health care organizations (eMethods and eTables 2-6 in Supplement 1).

### Exposures and Outcomes

The traditional definition of obesity was applied at baseline using race-specific BMI cutoffs per World Health Organization criteria.^10,11^ The new definition was also applied, which we further subclassified into 2 mutually exclusive phenotypes: (1) BMI-plus-anthropometric obesity, defined as BMI above the traditional obesity threshold plus at least 1 elevated anthropometric measure or BMI greater than 40, and (2) anthropometric-only obesity, defined as at least 2 elevated anthropometric measures with BMI below the traditional obesity threshold.^1^ BMI, waist circumference, waist-to-hip ratio, and waist-to-height ratio were evaluated according to sex- and/or race-specific thresholds (eMethods in Supplement 1).

Per the new definition, obesity was categorized at baseline as clinical or preclinical based on the presence of at least 1 prespecified manifestation of organ dysfunction and/or physical limitation (termed *organ dysfunction* throughout).^1^ These conditions were captured using *ICD* codes, survey data, and/or clinical laboratory results within the year prior to baseline (eMethods and eTable 2 in Supplement 1). Sensitivity analyses using *ICD* codes alone or from any time before baseline yielded similar prevalence estimates of clinical obesity.

Incident diabetes, cardiovascular events, and all-cause mortality were evaluated as longitudinal outcomes. Incident diabetes was defined using *ICD* codes, survey data, and/or clinical laboratory results among individuals without diabetes at baseline or within the first 6 months of follow-up (eMethods and eTable 3 in Supplement 1). Cardiovascular events were defined as myocardial infarction, stroke, or acute heart failure per *ICD* codes (eMethods and eTable 6 in Supplement 1). Eligibility for obesity pharmacotherapy was defined by current BMI-based indications as (1) BMI of 30 or higher or (2) BMI of 27 or higher plus hypertension, dyslipidemia, obstructive sleep apnea, or cardiovascular disease (using *ICD* codes, questionnaires, and/or laboratory data) per obesity guidelines, recent clinical trials, and FDA guidance (eTable 7 in Supplement 1).^2,4,5,6,7^

### Statistical Analysis

Baseline characteristics were summarized using medians (IQR) or proportions. Groups were compared using a Wilcoxon rank sum test and χ^2^ test. Logistic regression models estimated odds ratios (ORs) with 95% CI for organ dysfunction by obesity phenotype. Cause-specific Cox proportional hazards regression models estimated adjusted hazard ratios (AHRs) with 95% CIs for time-to-event outcomes, accounting for age, sex, race, and tobacco use (for cardiovascular events and all-cause mortality) (eMethods in Supplement 1). Analyses were performed using R, version 4.2.2 (R Program for Statistical Computing) on the AoU Researcher Workbench platform. Two-sided *P* < .05 indicated statistical significance.

## Results

### Prevalence of Obesity Per the New Definition

A total of 301 026 participants (183 633 [61.0%] female and 117 393 [39.0%] male; median age, 54 [IQR, 38-65] years) were included in the analysis. In terms of race, 4409 participants (1.5%) were American Indian or Alaska Native, 9037 (3.0%) were Asian, 59 347 (19.7%) were Black or African American, 1745 (0.6%) were Middle Eastern or North African, 160 158 (53.2%) were White, and 66 330 (22.0%) were of other race. In terms of ethnicity, a total of 54 308 participants (18.0%) identified as Hispanic or Latinx and 238 933 (79.4%) identified as non–Hispanic or Latinx.

**Figure 1.**
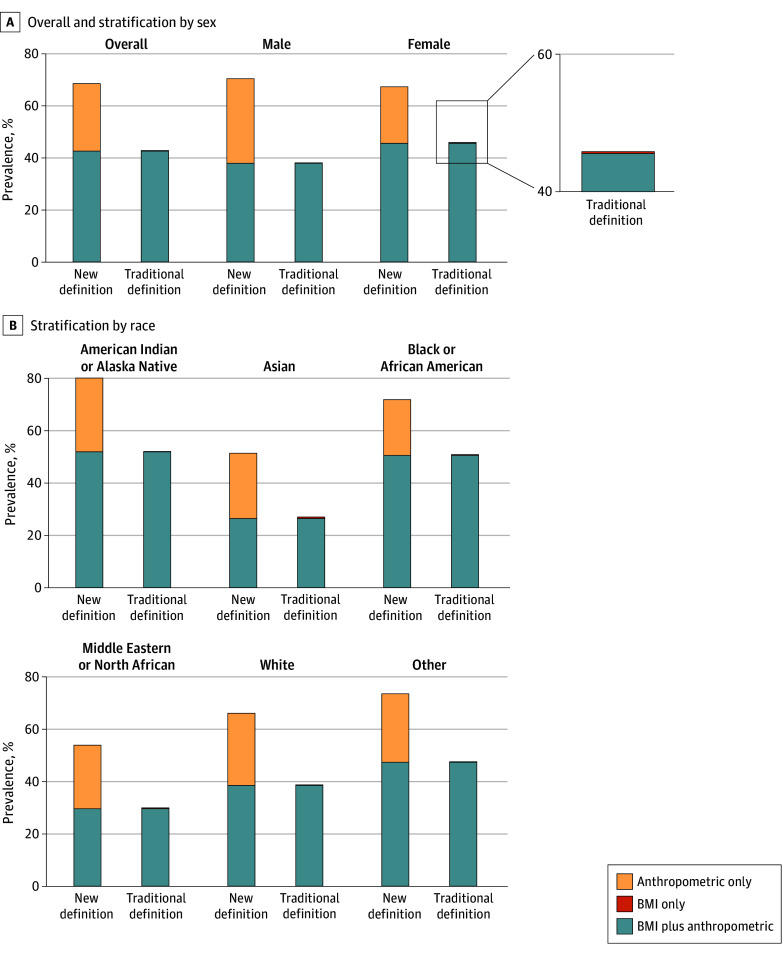
Prevalence of Obesity by the Traditional and New Definitions Among the All of Us Cohort The inset highlights the low prevalence of high body mass index (BMI) but nonelevated anthropometric measures under the traditional BMI-based obesity definition.

Using traditional BMI-based criteria, 128 992 individuals (42.9%) had obesity. Under the new definition, obesity prevalence increased nearly 60% to 206 361 individuals (68.6%) (Figure 1A). Nearly all individuals with obesity by the traditional definition also met criteria for BMI-plus-anthropometric obesity by the new definition. Among the overall sample, only 678 participants (0.2%) no longer met criteria for obesity per the new classification due to high BMI despite nonelevated anthropometric measures. In contrast, 78 047 participants (25.9%) did not have obesity per the traditional definition but were reclassified as having anthropometric-only obesity per the new framework.

While obesity prevalence by the new definition was similar between sexes, the distribution of obesity phenotypes differed by sex with a higher frequency of anthropometric-only obesity in male vs female participants (38 157 of 117 393 [32.5%] vs 39 890 of 183 633 [21.7%]; *P* < .001) (Figure 1A). Across racial groups, obesity prevalence increased by a similar absolute percentage when transitioning from the traditional to the new definition, with the highest relative increase among Asian individuals (90.3% relative increase from 2439 [27.0%] to 4641 [51.4%] of 9037 participants) (Figure 1B). By the new definition, obesity was more prevalent with older age, affecting 15 991 of 36 396 individuals aged 18 to 29 years (43.9%) and 35 268 of 45 018 individuals 70 years or older (78.3%) (*P* for trend < .001).

### Differential Characteristics of New Obesity Phenotypes

Under the new definition, compared with individuals with BMI-plus-anthropometric obesity, individuals with anthropometric-only obesity were older (median age, 54 [IQR, 40-64] vs 60 [IQR, 48-69] years, respectively; *P* < .001), were more commonly male (44 555 of 128 314 [34.7%] vs 38 157 of 78 047 [48.9%], respectively; *P* < .001), and had greater attainment of higher education (45 201 of 128 314 [35.2%] vs 35 124 of 78 047 [45.0%], respectively; *P* < .001) and income (eg, >$150 000, 9318 of 128 314 [7.3%] vs 9753 of 78 047 [12.5%], respectively; *P* < .001) (Table and eTable 8 in Supplement 1). Among those with obesity, the proportion with anthropometric-only obesity increased with age, affecting 4298 of 15 991 individuals aged 18 to 29 years (26.9%) and 18 671 of 35 268 individuals 70 years or older (52.9%) (*P* for trend < .001) (eFigure 3 in Supplement 1).

**Table.** Characteristics of Obesity Categories and Obesity Phenotypes Per the New Definition of Obesity^a^

Characteristic	Obesity category, No. (%)			*P* value^b^	Obesity phenotype, No. (%)		*P* value^c^
	Overall (N = 301 026)	Obesity absent (n = 94 665)	Obesity present (n = 206 361)		Anthropometric-only (n = 78 047)	BMI-plus- anthropometric (n = 128 314)	
Age, median (IQR), y	54 (38-65)	46 (31-61)	56 (43-66)	<.001	60 (48-69)	54 (40-64)	<.001
Sex							
Female	183 633 (61.0)	59 984 (63.4)	123 649 (59.9)	<.001	39 890 (51.1)	83 759 (65.3)	<.001
Male	117 393 (39.0)	34 681 (36.6)	82 712 (40.1)		38 157 (48.9)	44 555 (34.7)	
Race							
American Indian or Alaska Native	4409 (1.5)	877 (0.9)	3532 (1.7)	<.001	1243 (1.6)	2289 (1.8)	<.001
Asian	9037 (3.0)	4396 (4.6)	4641 (2.2)		2257 (2.9)	2384 (1.9)	
Black or African American	59 347 (19.7)	16 717 (17.7)	42 630 (20.7)		12 638 (16.2)	29 992 (23.4)	
Middle Eastern or North African	1745 (0.6)	805 (0.9)	940 (0.5)		424 (0.5)	516 (0.4)	
White	160 158 (53.2)	54 316 (57.4)	105 842 (51.3)		44 110 (56.5)	61 732 (48.1)	
Other^d^	66 330 (22.0)	17 554 (18.5)	48 776 (23.6)		17 375 (22.3)	31 401 (24.5)	
Ethnicity							
Hispanic or Latinx	54 308 (18.0)	13 977 (14.8)	40 331 (19.5)	<.001	14 219 (18.2)	26 112 (20.4)	<.001
Non–Hispanic or Latinx	238 933 (79.4)	78 349 (82.8)	160 584 (77.8)		61 568 (78.9)	99 016 (77.2)	
Missing	7785 (2.6)	2339 (2.5)	5446 (2.6)		2260 (2.9)	3186 (2.5)	
Smoking status							
Never smoker	172 287 (57.2)	58 012 (61.3)	114 275 (55.4)	<.001	40 745 (52.2)	73 530 (57.3)	<.001
Ever smoker	69 023 (22.9)	16 738 (17.7)	52 285 (25.3)		20 733 (26.6)	31 552 (24.6)	
Current smoker	49 589 (16.5)	16 599 (17.5)	32 990 (16.0)		13 917 (17.8)	19 073 (14.9)	
Missing	10 127 (3.4)	3316 (3.5)	6811 (3.3)		2652 (3.4)	4159 (3.2)	
BMI, median (IQR)	28.7 (24.6-33.9)	23.5 (21.4-25.7)	31.6 (28.0-36.5)	<.001	27.0 (25.2-28.5)	35.0 (32.1-39.6)	<.001
Waist circumference, median (IQR), cm	96.0 (84.0-108.2)	79.0 (73.5-84.2)	103.1 (95.0-113.8)	<.001	95.3 (90.2-101.0)	110.0 (101.5-119.6)	<.001
Hip circumference, median (IQR), cm	106.7 (99.0-116.8)	98.0 (92.5-103.0)	111.9 (104.1-122.0)	<.001	103.0 (98.2-107.6)	119.0 (111.8-128.2)	<.001
Elevated waist circumference							
Yes	165 315 (54.9)	209 (0.2)	165 106 (80.0)	<.001	45 080 (57.8)	120 026 (93.5)	<.001
No	135 711 (45.1)	94 456 (99.8)	41 255 (20.0)		32 967 (42.2)	8288 (6.5)	
Elevated WHR							
Yes	175 840 (58.4)	9158 (9.7)	166 682 (80.8)	<.001	70 907 (90.9)	95 775 (74.6)	<.001
No	125 186 (41.6)	85 507 (90.3)	39 679 (19.2)		7140 (9.1)	32 539 (25.4)	
Elevated waist-to-height ratio							
Yes	227 810 (75.7)	21 991 (23.2)	205 819 (99.7)	<.001	77 963 (99.9)	127 856 (99.6)	<.001
No	73 216 (24.3)	72 674 (76.8)	542 (0.3)		84 (0.1)	458 (0.4)	
Classification by traditional definition							
No obesity	172 034 (57.1)	93 987 (99.3)	78 047 (37.8)	<.001	78 047 (100)	0	<.001
Obesity	128 992 (42.9)	678 (0.7)	128 314 (62.2)		0	128 314 (100)	

Abbreviations: BMI, body mass index (calculated as weight in kilograms divided by height in meters squared); WHR, waist-to-hip ratio.

^a^ Presence and absence of obesity were defined based on the new framework. Obesity was further subdivided into BMI-plus-anthropometric and anthropometric-only phenotypes.

^b^ Compared between obesity groups.

^c^ Compared between anthropometric-only and BMI-plus-anthropometric groups.

^d^ Includes Native Hawaiian or Other Pacific Islander, multiracial, did not identify with any group, and preferred not to answer.

Among 78 047 individuals with anthropometric-only obesity, 17 426 (22.3%) had a BMI traditionally classified as normal or underweight, whereas the remainder fell within the traditional overweight category (eFigure 4 in Supplement 1). All 3 anthropometric measures were elevated among 37 856 of 78 047 people with anthropometric-only obesity (48.5%) and 92 200 of 128 314 with BMI-plus-anthropometric obesity (71.9%) (eFigure 5 in Supplement 1).

### Prevalence of Clinical Obesity Per the New Definition

By the new definition, 108 650 of 301 026 participants overall (36.1%) and 108 650 of 206 361 participants with obesity (52.7%) had clinical obesity (eFigures 6 and 7 in Supplement 1). Clinical obesity prevalence was comparable between sexes, but increased with age, both in the overall cohort and among those with obesity (both *P* for trend < .001) (eFigure 8 in Supplement 1). Specifically, among participants aged 18 to 29 years, 3107 of 36 396 overall (8.5%) and 3107 of 15 991 with obesity (19.4%) met criteria for clinical obesity. In contrast, among participants 70 years or older, 24 498 of 45 018 overall (54.4%) and 24 498 of 35 268 with obesity (69.5%) had clinical obesity. Clinical obesity was least common among Asian individuals, comprising 1784 of 9037 individuals overall (19.7%) and 1784 of 4641 with obesity (38.4%) (*P* < .001 each) (eFigure 9 in Supplement 1). Per traditional BMI categories, among 76 460 individuals with normal weight, 9388 (12.3%) and 7902 (10.3%) were classified as having preclinical and clinical obesity, respectively, under the new definition. Among 91 644 individuals with overweight, 31 403 (34.3%) and 29 218 (31.9%) met criteria for preclinical and clinical obesity, respectively (eFigure 10 in Supplement 1).

### Clinical Obesity by Obesity Phenotype

**Figure 2.**
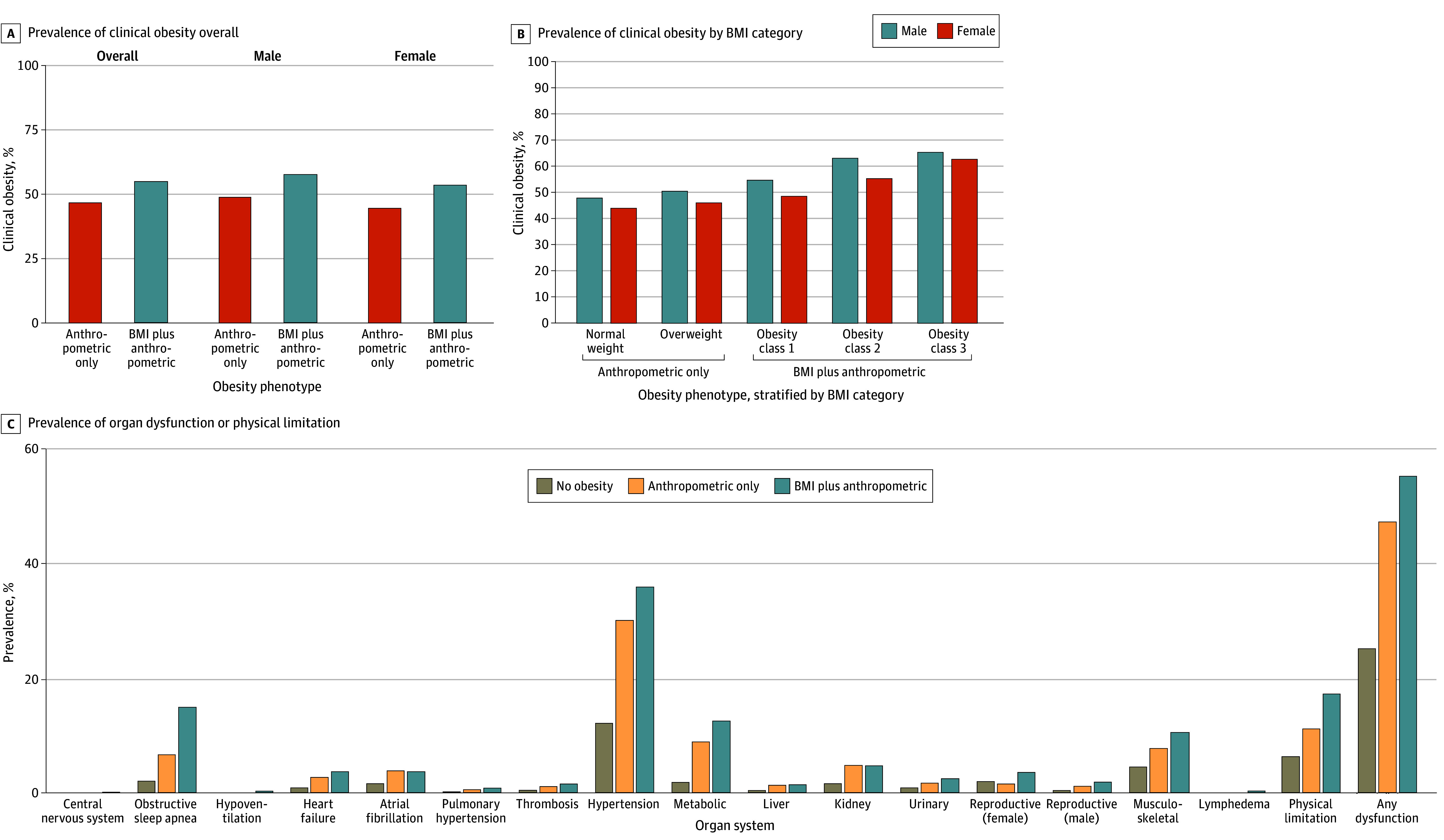
Differential Characteristics of New Obesity Phenotypes BMI indicates body mass index.

Individuals with BMI-plus-anthropometric obesity had a higher proportion of clinical obesity (71 457 of 128 314 [55.7%] vs 37 193 of 78 047 [47.7%]; *P* < .001) (Figure 2A) and a greater number of manifestations of organ dysfunction (median for BMI-plus-anthropometric obesity, 1 [IQR, 0-2]; median for anthropometric-only obesity, 0 [IQR, 0-1]; *P* < .001) (eFigure 11 in Supplement 1) compared with those with anthropometric-only obesity. Relatedly, the frequency of organ dysfunction among people with obesity progressively increased with higher BMI among male and female participants from normal weight (47.8% and 43.9%, respectively) to overweight (50.4% and 46.0%, respectively), obesity class 1 (54.7% and 48.5%, respectively), obesity class 2 (63.0% and 55.3%, respectively), and obesity class 3 (65.3% and 62.6%, respectively) (*P* for trend < .001) (Figure 2B and eFigure 12 in Supplement 1). In a multivariable model adjusting for age, sex, and race, odds ratios of organ dysfunction were 3.31 (95% CI, 3.24-3.37) for individuals with BMI-plus-anthropometric obesity and 1.76 (95% CI, 1.73-1.80) for those with anthropometric-only obesity vs individuals without obesity. Odds were comparable across sexes and races, although sex- and race-specific patterns of organ dysfunction emerged (eFigures 13 and 14 in Supplement 1). In the overall cohort, the most common manifestations of clinical obesity were hypertension, physical limitation, and obstructive sleep apnea (Figure 2C and eFigure 15 and eTable 9 in Supplement 1).

### Eligibility for Obesity Pharmacotherapy

Excluding individuals with diabetes who were otherwise candidates for glucagonlike peptide-1 receptor agonists (GLP1RAs), 111 467 of 249 235 individuals in the overall cohort (44.7%) met current BMI-based eligibility criteria for obesity pharmacotherapy.^2,4,5,6,7^ Notably, 15 495 of 69 894 individuals with clinical obesity (22.2%) by the new definition did not meet these criteria. Conversely, 57 068 of 111 467 participants eligible for obesity pharmacotherapy per BMI-based indications (51.2%) did not have clinical obesity (eFigure 16 in Supplement 1).

### Longitudinal Health Outcomes by Obesity Definition and Phenotype

**Figure 3.**
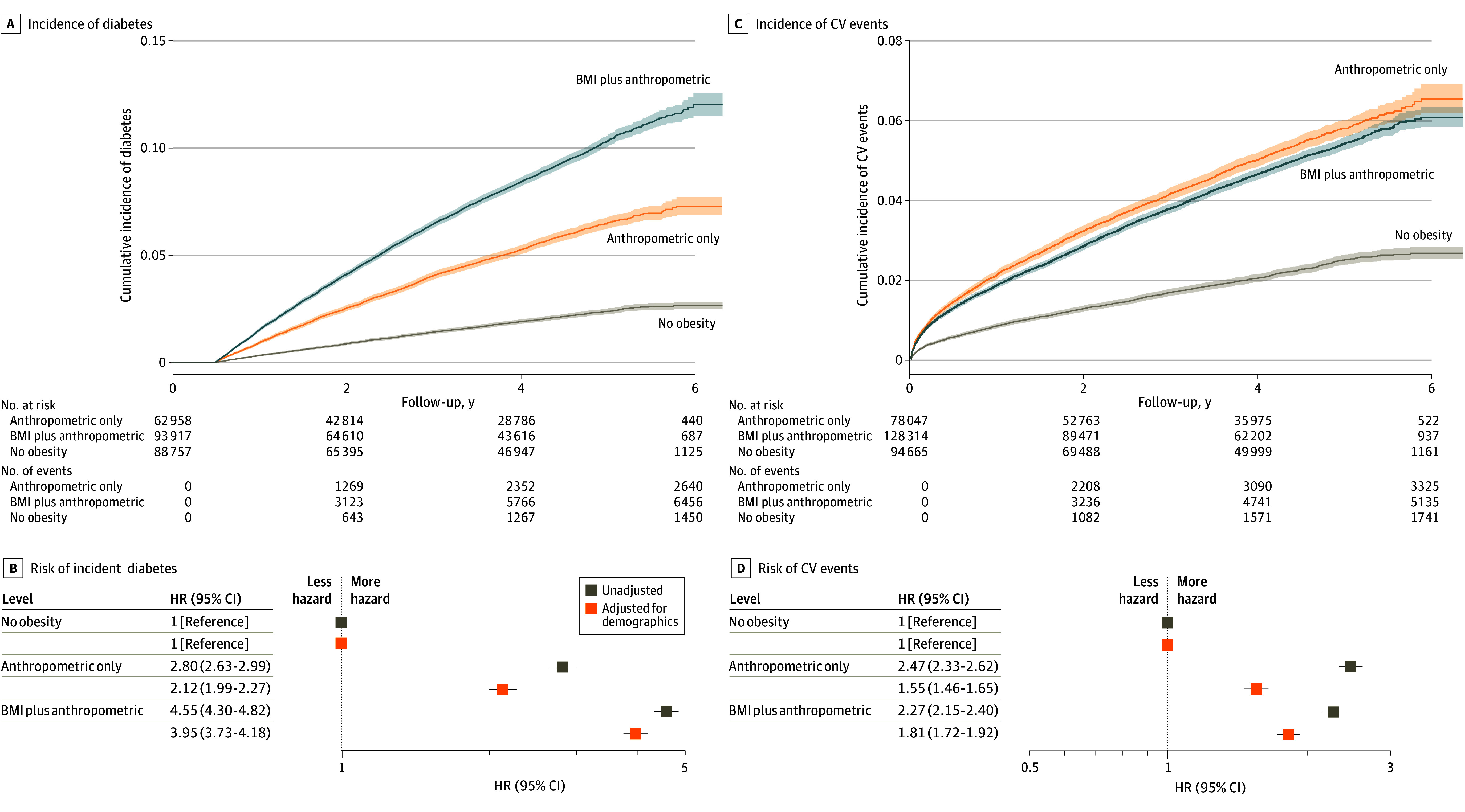
Longitudinal Risks of Adverse Health Outcomes by New Obesity Phenotype in the All of Us Cohort A and C, Cumulative incidence plots with shaded 95% CIs depict the probability of each longitudinal outcome by obesity phenotype. B and D, Forest plots display hazard ratios (HRs) with 95% CIs for each longitudinal health outcome among subgroups. The unadjusted model includes only the exposure variable (obesity phenotype). The demographics model adjusted for age, sex, and race, with additional adjustment for smoking status in the analysis of cardiovascular (CV) events. BMI indicates body mass index.

By the traditional and new definitions, obesity conferred elevated risks of incident diabetes (AHRs, 2.60 [95% CI, 2.50-2.70] vs 3.21 [95% CI, 3.03-3.39]), cardiovascular events (AHRs, 1.39 [95% CI, 1.34-1.45] vs 1.70 [95% CI, 1.62-1.80]), and all-cause mortality (AHRs 1.10 [95% CI, 1.03-1.18] vs 1.21 [95% CI, 1.12-1.31]), with higher AHRs under the new framework (eFigure 17 in Supplement 1). Using the new definition, BMI-plus-anthropometric obesity carried the greatest risk of incident diabetes (AHR, 3.95 [95% CI, 3.73-4.18]), followed by anthropometric-only obesity (AHR, 2.12 [95% CI, 1.99-2.27]), compared with no obesity (Figure 3A and B). In contrast, risks of cardiovascular events (BMI-plus-anthropometric AHR, 1.81 [95% CI, 1.72-1.92]; anthropometric-only AHR, 1.55 [95% CI, 1.46-1.65]) and all-cause mortality (AHRs, 1.22 [95% CI, 1.12-1.34] and 1.20 [95% CI, 1.09-1.31], respectively) were similarly elevated across obesity phenotypes relative to no obesity (Figure 3C and D and eFigure 18 in Supplement 1).

### Longitudinal Health Outcomes by Clinical Obesity Status

**Figure 4.**
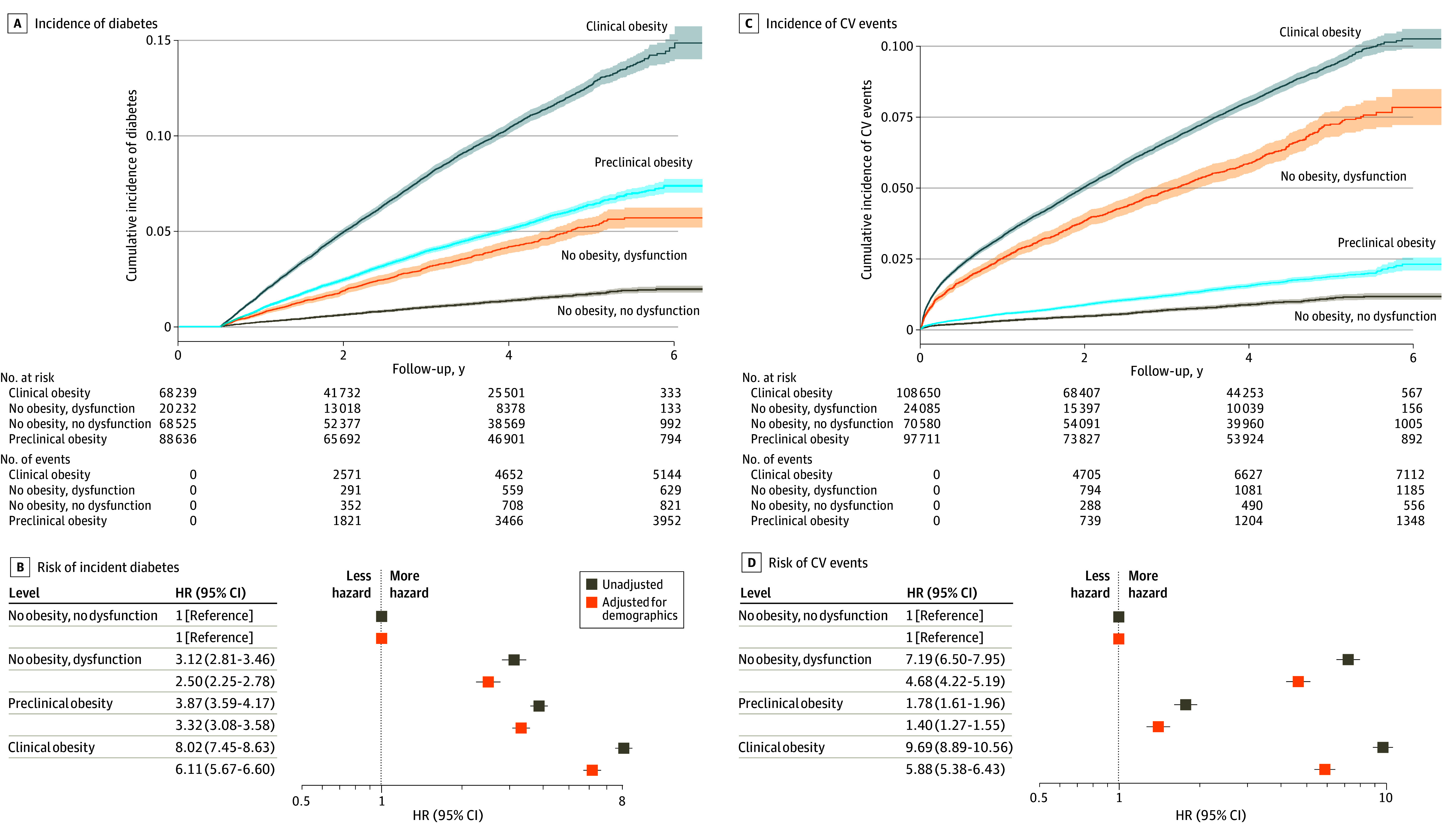
Longitudinal Risks of Adverse Health Outcomes by Clinical Obesity Status Per the New Definition of Obesity in the All of Us Cohort A and C, Cumulative incidence plots with shaded 95% CIs depict the probability of each longitudinal outcome among individuals with clinical obesity, preclinical obesity, and no obesity with or without organ dysfunction. B and D, Forest plots display hazard ratios (HRs) with 95% CIs for each longitudinal health outcome among these 4 subgroups. The unadjusted model includes only the exposure variable (obesity and organ dysfunction status). The demographics model adjusted for age, sex, and race, with additional adjustment for smoking status in the analysis of cardiovascular (CV) events. BMI indicates body mass index.

Compared with individuals without obesity or organ dysfunction, clinical obesity according to the new definition conferred the highest risk of incident diabetes (AHR, 6.11 [95% CI, 5.67-6.60]), followed by preclinical obesity (AHR, 3.32 [95% CI, 3.08-3.58]) and organ dysfunction in the absence of obesity (AHR, 2.50 [95% CI, 2.25-2.78]) (Figure 4A and B). In contrast, clinical obesity (AHR, 5.88 [95% CI, 5.38-6.43]) and organ dysfunction in the absence of obesity (AHR, 4.68 [95% CI, 4.22-5.19]) were both associated with a highly increased risk of cardiovascular events, whereas the risk elevation associated with preclinical obesity was more moderate (AHR, 1.40 [95% CI, 1.27-1.55]) (Figure 4C and D). Similarly, clinical obesity (AHR, 2.71 [95% CI, 2.41-3.05]) and organ dysfunction in the absence of obesity (AHR, 2.82 [95% CI, 2.45-3.26]) were both associated with increased risk of all-cause mortality, whereas there was no risk elevation observed for preclinical obesity (AHR, 1.09 [95% CI, 0.96-1.25]) (eFigure 19 in Supplement 1). Associations were consistent across age strata (eFigure 20 in Supplement 1).

## Discussion

In this analysis, we applied the *Lancet* Commission’s new definition of obesity to AoU, a large, diverse US cohort. Under this framework, 68.6% of participants met criteria for obesity, a 60% increase in prevalence from the traditional BMI-based definition. This rise was entirely driven by inclusion of individuals with anthropometric-only obesity, defined as having at least 2 elevated anthropometric measures despite a nonelevated BMI. Meanwhile, nearly all individuals classified as having obesity by the traditional definition met criteria under the new framework for BMI-plus-anthropometric obesity, defined as having an elevated BMI plus at least 1 elevated anthropometric measure or BMI of greater than 40. Our findings suggest that the new obesity definition effectively stratified individuals at high-risk of organ dysfunction and long-term complications while exposing potential public health implications and areas for further study.

Based on the new definition, approximately 1 in 4 participants in the AoU cohort met criteria for anthropometric-only obesity. Notably, an estimated 1 in 4 of these individuals had BMI in the traditionally normal (nonoverweight) range. Despite their nonelevated BMI, individuals with anthropometric-only obesity exhibited a heightened prevalence of organ dysfunction and risk of incident diabetes compared with those without obesity, although to a moderately lesser extent than those with BMI-plus-anthropometric obesity. Meanwhile, risks of cardiovascular events and mortality were similarly increased across obesity phenotypes.

These findings support the new definition of obesity by identifying individuals with anthropometric-only obesity as having a heightened risk of adverse health outcomes. Concordant with our data, prior studies have repeatedly shown that central adiposity, independently of BMI, is a key factor associated with cardiometabolic disease.^12,13,14,15^ However, our data also suggest that anthropometric-only obesity (reflecting excess abdominal adiposity) and BMI-plus-anthropometric obesity (reflecting excess total body adiposity) may represent distinct clinical entities with unique pathophysiologic mechanisms and outcomes. In this regard, risk factors and treatment strategies that have been studied in the context of traditional BMI-based obesity may be more applicable to BMI-plus-anthropometric obesity, whereas the drivers and optimal management of anthropometric-only obesity remain less well understood. The sociodemographic differences that we observed between subgroups highlight the importance of investigating the variable contributions of aging, sex hormones, genetics, and social determinants of health to each obesity phenotype to inform targeted prevention strategies. Furthermore, as BMI appears to compound the adverse effects of central adiposity, our findings underscore the continued need to address elevated BMI as a cornerstone of obesity management. At the same time, clinical trials are critically needed to evaluate interventions that specifically target excess abdominal adiposity in individuals with anthropometric-only obesity who may not derive the same benefit from traditional weight-centric approaches. Notably, lifestyle interventions such as exercise^16,17,18^ and pharmacologic therapies including tesamorelin^19,20,21^ have been shown to reduce visceral adiposity without significantly altering body weight and thus may warrant further investigation in the context of anthropometric-only obesity.

Among the AoU cohort, we found that approximately half of participants classified as having obesity under the new definition also exhibited organ dysfunction and/or physical limitation consistent with clinical obesity. Our analyses suggest that the new definition of clinical obesity appropriately designates individuals with obesity who are at the highest long-term risk of incident diabetes, cardiovascular events, and mortality. However, our findings also demonstrate that preclinical obesity is not a benign entity. Individuals with preclinical obesity exhibited an increased risk of diabetes and cardiovascular events compared with those without obesity or organ dysfunction. Moreover, clinical obesity prevalence increased with age, suggesting that preclinical obesity may progress to clinical obesity over time. Further research is needed to distinguish which individuals with preclinical obesity are at highest risk of adverse health outcomes to allow for targeted, cost-effective interventions among this heterogenous population.

While the definition of clinical obesity appropriately designates individuals with obesity who face the highest long-term health risks, our findings also underscore the substantial risks conferred by organ dysfunction itself, even in the absence of obesity. This was particularly evident for cardiovascular events and mortality, whereby individuals with organ dysfunction, irrespective of obesity status, exhibited comparably elevated risk. Since obesity was associated with an increased likelihood of organ dysfunction in our analysis, these findings support a framework in which obesity may contribute to organ dysfunction, which in turn may predispose to downstream clinical events. This interpretation aligns with established cardiovascular risk prediction tools such as the Pooled Cohort Equation,^22^ the Systemic Coronary Risk Evaluation 2 (SCORE2),^23^ and the Framingham Risk Score,^24^ which incorporate markers of organ dysfunction but not BMI. At the same time, given the elevated risks of diabetes and cardiovascular events that we observed among individuals with preclinical obesity, organ dysfunction may not be required for adiposity to confer harm. Further research is needed to determine whether treating obesity can reverse organ dysfunction and whether this may translate to improved long-term outcomes.

Across racial groups, implementation of the new obesity definition led to the greatest relative rise in obesity prevalence among Asian individuals. Distinct profiles of clinical obesity emerged by race, with metabolic dysfunction being a predominant manifestation among this group. This aligns with prior observations that certain Asian populations have heightened risks of diabetes and cardiovascular disease relative to other racial groups.^25,26,27,28,29^

Strikingly, nearly 80% of participants 70 years or older in the AoU cohort met criteria for obesity, which constitutes a doubling in obesity prevalence from the traditional BMI-based definition. Furthermore, over half of individuals 70 years or older exhibited clinical obesity. The predisposition to anthropometric-only obesity in older adults aligns with prior studies that have shown a tendency toward central fat accumulation with older age and the menopausal transition.^30,31,32,33^ Clinical obesity conferred a similarly elevated risk of incident diabetes, cardiovascular events, and mortality across age groups, providing a rationale for guideline-directed pharmacologic and/or surgical management among older adults. These findings may have significant public health implications, presaging cost challenges for health payer systems, including Medicare.

Finally, the new definition may substantially reshape obesity pharmacotherapy prescribing patterns. Approximately 45% of our cohort met current BMI-based indications for antiobesity medication, which falls midway between the prevalence of clinical obesity and overall obesity under the new framework. Since the new guideline recommends pharmacologic therapy for all individuals with clinical obesity and select individuals with preclinical obesity,^1^ overall use of obesity pharmacotherapy may remain comparable to current levels. However, the composition of the treatment-eligible population is likely to shift; as many as half of those currently eligible for medication may no longer qualify due to absence of clinical obesity (eg, a person with BMI of 34 and no organ dysfunction), whereas approximately 1 in 4 individuals with clinical obesity who are newly designated for treatment would not meet eligibility criteria for recent GLP1RA clinical trials^4,5^ or current FDA-labeled indications^7^ (eg, a person with BMI of 24, central adiposity, and organ dysfunction). Thus, implementation of the new framework may have significant ramifications for patients, including existing GLP1RA users,^34^ and creates a compelling need to evaluate use of antiobesity medications within this redefined target population.

### Strengths and Limitations

To our knowledge, this is the first study to comprehensively examine the clinical implications of the new obesity definition and its relevance to long-term health outcomes. The study’s strengths include a large, diverse sample and evaluation of both cross-sectional and longitudinal health conditions. The AoU cohort provides access to longitudinal EHR data, which may be less well-captured in other cohorts. Although Black or African American individuals are somewhat overrepresented,^35^ the obesity prevalence in our sample using traditional BMI-based criteria (42.9%) closely aligns with Centers for Disease Control and Prevention estimates (40.3%),^36^ supporting the generalizability of our findings.

A key limitation of our analysis is its reliance on *ICD* codes, survey responses, and laboratory results to classify clinical obesity, which may not fully capture organ dysfunction or physical limitation. Nonetheless, our findings remained robust in sensitivity analyses using alternate diagnostic criteria and data capture windows. Additionally, while the guideline specifies that organ dysfunction must be secondary to obesity, we could not establish causality. Consequently, we classified all individuals with both obesity and organ dysfunction as having clinical obesity, reflecting how the guideline is likely to be applied in clinical practice where establishing causality is also challenging. Ideally, causality would be inferred if organ dysfunction improved following treatment of obesity.^37^ However, this presumes reversibility and introduces a circular dilemma whereby diagnosis is required to justify treatment, yet treatment response is required to confirm diagnosis. Last, in the absence of a gold standard for defining obesity, we relied on the new framework’s ability to stratify future health risk as an indirect measure of clinical utility. While the new obesity framework is more complex and resource-intensive than the traditional BMI-based definition, its capacity to appropriately risk-stratify individuals may justify the effort. Smart tools integrated into EHR systems and online calculators could enhance adoption.

## Conclusions

In this cohort study, implementation of a new obesity definition resulted in a substantial rise in obesity prevalence, particularly among older adults, that may have profound public health and financial implications. Our findings support inclusion of anthropometric-only obesity within the new obesity definition and affirm the value of clinical obesity in identifying individuals at highest risk of adverse health outcomes. At the same time, our results highlight critical gaps in knowledge regarding anthropometric-only obesity, preclinical obesity, and the shifting target population for obesity pharmacotherapy, underscoring the need for further research to inform evidence-based care of these groups.

## Notes

Supplement 1. **eMethods.** Design, Data Sources, Definitions, and Analyses **eFigure 1.** Study Design Timeline Including Covariate Assessment, Baseline, and Follow-Up Windows **eFigure 2.** Flow Diagram of the Inclusion/Exclusion Criteria of the Study Analysis **eTable 1.** Characteristics of Participants Included vs Excluded From the Analysis Due to Incomplete Anthropometric Data **eTable 2.** Data Sources for the Phenotypic Curation of Manifestations of Organ Dysfunction as Defined by the New Definition **eTable 3.** *ICD* Concepts Mapped to the Organ Dysfunction Categories **eTable 4.** Other Curated Phenotypic Information **eTable 5.** Smoking-Related *ICD* Codes **eTable 6.** Cardiovascular-Related *ICD* Codes **eTable 7.** BMI-Based Eligibility Criteria for Obesity Pharmacotherapy **eTable 8.** Social Determinants of Health by Obesity Status and Phenotype Per the New Definition **eTable 9.** Organ Dysfunction by Obesity Status and Phenotype Per the New Definition **eFigure 3.** Distribution of New Obesity Phenotypes by Age **eFigure 4.** Distribution of BMI Among Individuals With Anthropometric-Only Obesity **eFigure 5.** Elevated Anthropometrics by New Obesity Phenotype **eFigure 6.** Prevalence of Clinical Obesity by the Traditional and New Definitions Among the All of Us Cohort **eFigure 7.** Proportion of Individuals With Obesity Per the New Definition Meeting Clinical Obesity Criteria **eFigure 8.** Prevalence of Obesity and Clinical Obesity by the Traditional and New Definitions Across Age Groups **eFigure 9.** Prevalence of Obesity and Clinical Obesity Per the New Definition by Race Among the All of Us Cohort **eFigure 10.** Prevalence of Obesity by the New Definition Stratified by Traditional BMI Category **eFigure 11.** Burden of Organ Dysfunction by New Obesity Phenotype **eFigure 12.** Association of Organ Dysfunction With BMI in Individuals With Obesity Per the New Definition **eFigure 13.** Differential Characteristics of New Obesity Phenotypes by Sex **eFigure 14.** Differential Characteristics of New Obesity Phenotypes Across Racial Groups **eFigure 15.** Patterns of Organ Dysfunction Among Individuals With Clinical Obesity Per the New Definition **eFigure 16.** Obesity Pharmacotherapy Eligibility by Classification Scheme **eFigure 17.** Longitudinal Risks of Adverse Health Outcomes by Traditional vs New Obesity Definitions **eFigure 18.** Longitudinal Risks of All-Cause Mortality by New Obesity Phenotype **eFigure 19.** Longitudinal Risks of All-Cause Mortality by Clinical Obesity Status Per the New Definition **eFigure 20.** Associations of Clinical Obesity Status Per the New Definition With Longitudinal Health Outcomes by Age Strata
eReferences

Supplement 2.
Data Sharing Statement
