# Supplementary material for: Implications of a New Obesity Definition Among the All of Us Cohort
Source: JAMA Netw Open. 2025 Oct 15;8(10):e2537619. doi: 10.1001/jamanetworkopen.2025.37619 (PMC12529213; doi:10.1001/jamanetworkopen.2025.37619)
Supplement: Supplement 1. — eMethods. Design, Data Sources, Definitions, and Analyses eFigure 1. Study Design Timeline Including Covariate Assessment, Baseline, and Follow-Up Windows eFigure 2. Flow Diagram of the Inclusion/Exclusion Criteria of the Study Analysis eTable 1. Characteristics of Participants Included vs Excluded From the Analysis Due to Incomplete Anthropometric Data eTable 2. Data Sources for the Phenotypic Curation of Manifestations of Organ Dysfunction as Defined by the New Definition eTable 3. ICD Concepts Mapped to the Organ Dysfunction Categories eTable 4. Other Curated Phenotypic Information eTable 5. Smoking-Related ICD Codes eTable 6. Cardiovascular-Related ICD Codes eTable 7. BMI-Based Eligibility Criteria for Obesity Pharmacotherapy eTable 8. Social Determinants of Health by Obesity Status and Phenotype Per the New Definition eTable 9. Organ Dysfunction by Obesity Status and Phenotype Per the New Definition eFigure 3. Distribution of New Obesity Phenotypes by Age eFigure 4. Distribution of BMI Among Individuals With Anthropometric-Only Obesity eFigure 5. Elevated Anthropometrics by New Obesity Phenotype eFigure 6. Prevalence of Clinical Obesity by the Traditional and New Definitions Among the All of Us Cohort eFigure 7. Proportion of Individuals With Obesity Per the New Definition Meeting Clinical Obesity Criteria eFigure 8. Prevalence of Obesity and Clinical Obesity by the Traditional and New Definitions Across Age Groups eFigure 9. Prevalence of Obesity and Clinical Obesity Per the New Definition by Race Among the All of Us Cohort eFigure 10. Prevalence of Obesity by the New Definition Stratified by Traditional BMI Category eFigure 11. Burden of Organ Dysfunction by New Obesity Phenotype eFigure 12. Association of Organ Dysfunction With BMI in Individuals With Obesity Per the New Definition eFigure 13. Differential Characteristics of New Obesity Phenotypes by Sex eFigure 14. Differential Characteristics of New Obesity Phenotypes Across Racial Groups eFigure 15. Patte [file jamanetwopen-e2537619-s001.pdf]

Supplementary Online Content

Fourman LT, Awwad A, Gutiérrez-Sacristán A, et al. Implications of the *Lancet* Commission obesity definition among the All of Us cohort. *JAMA Netw Open.* 2025;8(10):e2537619. doi:10.1001/jamanetworkopen.2025.37619

eMethods. Design, Data Sources, Definitions, and Analyses
eFigure 1. Study Design Timeline Including Covariate Assessment, Baseline, and Follow-Up Windows
eFigure 2. Flow Diagram of the Inclusion/Exclusion Criteria of the Study Analysis
eTable 1. Characteristics of Participants Included vs Excluded From the Analysis Due to Incomplete Anthropometric Data
eTable 2. Data Sources for the Phenotypic Curation of Manifestations of Organ Dysfunction as Defined by the New Definition
eTable 3. *ICD* Concepts Mapped to the Organ Dysfunction Categories
eTable 4. Other Curated Phenotypic Information
eTable 5. Smoking-Related *ICD* Codes
eTable 6. Cardiovascular-Related *ICD* Codes
eTable 7. BMI-Based Eligibility Criteria for Obesity Pharmacotherapy
eTable 8. Social Determinants of Health by Obesity Status and Phenotype Per the New Definition
eTable 9. Organ Dysfunction by Obesity Status and Phenotype Per the New Definition
eFigure 3. Distribution of New Obesity Phenotypes by Age
eFigure 4. Distribution of BMI Among Individuals With Anthropometric-Only Obesity
eFigure 5. Elevated Anthropometrics by New Obesity Phenotype
eFigure 6. Prevalence of Clinical Obesity by the Traditional and New Definitions Among the All of Us Cohort
eFigure 7. Proportion of Individuals With Obesity Per the New Definition Meeting Clinical Obesity Criteria
eFigure 8. Prevalence of Obesity and Clinical Obesity by the Traditional and New Definitions Across Age Groups
eFigure 9. Prevalence of Obesity and Clinical Obesity Per the New Definition by Race Among the All of Us Cohort
eFigure 10. Prevalence of Obesity by the New Definition Stratified by Traditional BMI Category
eFigure 11. Burden of Organ Dysfunction by New Obesity Phenotype
eFigure 12. Association of Organ Dysfunction With BMI in Individuals With Obesity Per the New Definition
eFigure 13. Differential Characteristics of New Obesity Phenotypes by Sex
eFigure 14. Differential Characteristics of New Obesity Phenotypes Across Racial Groups
eFigure 15. Patterns of Organ Dysfunction Among Individuals With Clinical Obesity Per the New Definition

© 2025 Fourman LT et al. *JAMA Network Open*.

eFigure 16. Obesity Pharmacotherapy Eligibility by Classification Scheme
eFigure 17. Longitudinal Risks of Adverse Health Outcomes by Traditional vs New Obesity Definitions
eFigure 18. Longitudinal Risks of All-Cause Mortality by New Obesity Phenotype
eFigure 19. Longitudinal Risks of All-Cause Mortality by Clinical Obesity Status per the New Definition
eFigure 20. Associations of Clinical Obesity Status Per the New Definition With Longitudinal Health Outcomes by Age Strata
eReferences

This supplementary material has been provided by the authors to give readers additional information about their work.

© 2025 Fourman LT et al. *JAMA Network Open*.

eMethods. Design, Data Sources, Definitions, and Analyses

All of Us Research Program (AoU) Design

The AoU research initiative was launched May 6th, 2018, and it is funded by the National Institutes of Health (NIH). It is the largest cohort study in the US, and it incorporates different and diverse data types (1,2).

We used data from the AoU Researcher Workbench, which included data that has been collected from participants through surveys (Participant Provided Information [PPI]), physical measurements, electronic health records (EHRs), wearables, and biospecimens when enrolling in and throughout the duration of the program. The provided information is then transformed, harmonized, and anonymized into custom tables according to the Observational Medical Outcomes Partnership (OMOP) Common Data Model (CDM) version 5.3 when possible.

The data in AoU is curated by privacy experts to remove all personally identifying information from participant records. For this project, we used the controlled tier data, version release C2024Q3R4; 2/3/2025, with a collecting data cutoff point of 10/1/2023. This tier contains participant-level data with fewer transformations compared to the registered tier data.

Only researchers that were authorized to have access to the controlled tier dataset were able to view and access the AoU workbench.

Study Design

The baseline for participants was defined as the date of the physical measurements (**eFigure 1**). Out of 375,752 non-pregnant participants with linked EHRs and available physical measurements, 301,026 met our inclusion criteria (**eFigure 2**). Participants were evaluated for concurrent "baseline" conditions through assessing a retrospective window of 1 year prior to the baseline. Clinical assessment relied on multiple data sources as explained later (**eTables 2-6**).

Data Sources for Assessment of Demographic and Clinical Information

We assessed clinical information utilizing the following sources:

1) Electronic health records (EHR) - Many participants agreed to share their electronic health records (EHR) with the program. The All of Us program removes personal identifiers from participants' EHR data before adding this information to the Research Hub. We established a list of International Classification of Diseases (ICD) codes that reflect the manifestations of organ dysfunction that define clinical obesity as specified in the *Lancet* Commission guidelines (**eTables 2 and 3**). We constructed similar lists of ICD codes to reflect tobacco use (**eTable 5**) and longitudinal health outcomes (**eTables 3 and 6**). Using EHR, we collected conditions based on these ICD codes.

2) Survey data - Participants in the All of Us cohort completed surveys at baseline and had the option to complete additional surveys longitudinally; data was collected from more than 633,000 participants. Information was extracted from the following surveys for the purpose of this analysis:

   Baseline surveys:

© 2025 Fourman LT et al. *JAMA Network Open*.

a. The basics
b. Overall health
c. Lifestyle

Follow-up surveys:

d. Personal and overall family history

The baseline surveys were available for all included participants at baseline, whereas the personal and overall family history was included only if the participant completed it by the day of the physical measurement. These surveys were used as a complementary source in addition to the available EHR data to increase our ability to better capture clinical phenotypic data. Survey questions used to ascertain phenotypic and demographic information are provided in **eTables 2 and 4**.

Demographic and sociodemographic factors:

AoU collects self-reported sex, race and ethnicity data from participants via the basics survey, which is administered at enrollment. The exact question and the available answers from the survey are detailed in **eTable 4**. For the race and ethnicity variables, we are using the AoU transformed curated variables as is described in details on the AoU website. We further categorize race into the following groups: Asians, American Indian or Alaska Native, Black or African American, Middle Eastern or North African, White and Other. The 'Other' group includes Native Hawaiian or Other Pacific Islander, individuals that chose more than one population, did not identify with any group or preferred not to answer.

3) Laboratory data: Clinical laboratory data was retrieved from the EHR, specifically hemoglobin A1C, fasting glucose, glucose, high-density lipoprotein cholesterol (HDL-C), and triglycerides.

4) Physical measurements

At the baseline visit, a standardized set of physical measurements was taken from participants. Measurements were taken by trained study staff following AoU standard operating procedures (the detailed protocol for height, weight, waist and hip measurements can be found on All of Us website) (3). Measurements were then recorded in HealthPro, the platform used by AoU for collecting results of physical measurements, processing biospecimens, and viewing individual-level participant operational data.

Height and weight: Trained staff measured height using a stadiometer and weight using a calibrated digital or non-digital scale with participants standing without shoes and in light clothing. Equipment was maintained and calibrated according to protocol. Special procedures were followed for pregnant participants and individuals unable to stand, including self-reported values when necessary. Height was recorded in centimeters to the nearest millimeter, and weight in kilograms to the nearest 0.1 kg. Standardized techniques ensured consistency across sites and participants, with modifications documented when standard procedures could not be followed.

Waist and hip circumference: Participants were asked to remove bulky items or clothing, to stand with feet positioned close together and weight evenly distributed, and to relax and take a few deep breaths. Waist circumference was measured at the end of normal expiration using a non-stretchable tape at the narrowest part of the waist, the top of the iliac crest, or the umbilicus—depending on participant anatomy—with location documented. Hip circumference was measured at the widest part of the buttocks. For both waist and hip, two measurements were taken, and a third was obtained if the first two differed by more than 1.0 cm. The final value was the average of the two closest measurements (or all three if equidistant). Participants who were pregnant, wheelchair users, or declined measurement were excluded per protocol.

© 2025 Fourman LT et al. *JAMA Network Open*.

Definition of Obesity Based on the Traditional and New Criteria

Definitions of Obesity	
**Traditional Obesity**	Elevated BMI ($\frac{weight}{height^2}$) only
**New *Lancet* Commission Obesity**	BMI-plus-anthropometric obesity: Elevated BMI plus at least 1 elevated anthropometric OR BMI >40 kg/m^2 (elevated defined below)
	Anthropometric-only obesity: At least 2 elevated anthropometrics with non-elevated BMI
**Clinical Obesity**	Obesity plus at least one manifestation of organ dysfunction and/or physical limitation as defined by the *Lancet* Commission guidelines

**BMI Categories<br>(Based on WHO-recommended cutoffs)**	
Asians:<br><br>Elevated BMI: ≥27.5 kg/m^2<br><br>Traditional BMI Categories<br>Underweight: <18.5 kg/m²<br>Normal: BMI 18.5 to <23 kg/m²<br>Overweight: BMI 23 to <27.5 kg/m²<br>Obesity Class I: BMI 27.5 to <35 kg/m²<br>Obesity Class II: BMI 35 to <40 kg/m²<br>Obesity Class III: BMI ≥40 kg/m²	All Others:<br><br>Elevated BMI: ≥30 kg/m^2<br><br>Traditional BMI Categories<br>Underweight: <18.5 kg/m^2<br>Normal: BMI 18.5 to <25 kg/m^2<br>Overweight: BMI 25 to <30 kg/m²<br>Obesity Class I: BMI 30 to <35 kg/m²<br>Obesity Class II: BMI 35 to <40 kg/m²<br>Obesity Class III: BMI ≥40 kg/m²

**Anthropometric Measurements<br>(Based on sex- and race-specific cut-offs,<br>per *Lancet* Commission supplement)**		
	Males	Females
**Elevated Waist Circumference**	Asians: ≥90 cm<br>All Others: ≥102 cm	Asians: ≥80 cm<br>All Others: ≥88 cm
**Elevated Waist-to-Height Ratio**	≥0.5	≥0.5
**Elevated Waist-to-Hip Ratio**	≥0.9	≥0.85

© 2025 Fourman LT et al. *JAMA Network Open*.

Definition of Longitudinal Outcomes

We examined three longitudinal outcomes that were defined as followed:

1) Incident diabetes:

   Individuals with any history of diabetes (as captured by either ICD codes, surveys or abnormal laboratory measurement) were excluded. Incident diabetes was defined as the first occurrence of: 1) ICD code of type 2 diabetes (**eTable 3**), 2) abnormal laboratory measurement (fasting glucose ≥126 mg/dL, hemoglobin A1C ≥6.5%, glucose ≥200 mg/dL) 3) survey. Incident cases in the first 6 months were excluded and assumed to have been prevalent at baseline but not yet reflected in the ICD billing codes; participants were censored at last contact (defined as the last available date of any participant record in the All of Us database) or death.

2) Cardiovascular (CV) event:

   Cardiovascular event was defined as the incidence of either acute myocardial infarction, stroke, or heart failure. All were captured using ICD codes (**eTable 6),** and the incidence was defined as the occurrence of the first ICD code corresponding to any of those conditions. Follow-up began immediately after the baseline visit; participants were censored at last contact (defined as the last available date of any participant record in the All of Us database) or death.

3) All-cause mortality:

   The death data was collected from two main sources: (1) EHR, (2) HealthPro. A total of 422 records did not have a recorded death date and were subsequently excluded from this analysis. Follow-up began immediately after the baseline visit; participants were censored at last contact (defined as the last available date of any participant record in All of Us database).

© 2025 Fourman LT et al. *JAMA Network Open*.

Statistical Analysis

Age adjusted prevalence:

To compare prevalence across racial and sex subgroups, we employed age-adjusted prevalence approach using the direct standardization method. This helps eliminate possible differences in the observed rates that stem from different age compositions of the compared groups.

This approach requires the use of a standard age distribution. The standard for age-adjusting death rates and estimates from surveys in *Health, United States* is the projected year 2000 U.S.(4)

Age-adjusted prevalence was calculated using the formula:(5)

$$\sum_{i=1}^{n} r_i(p_i/P)$$

Where:

r_i = Prevalence in age group i in the population of interest

p_i = Standard population in age group i

P = total standard population across all age groups $\sum_{i=1}^{n} p_i$

n= Total number of age groups over the age range of the age-adjusted rate

Survival analysis:

Each outcome was assessed independently of the other outcomes. Start of follow-up was defined as the day of the physical measurements. Follow-up ended at the earliest of the following: occurrence of the outcome, last contact (based on the most recent date available from the EHR or survey data), or death. For incident diabetes and cardiovascular events, death was treated as a competing event, and participants were censored at the time of death or last contact. The proportional hazard assumptions were met when inspecting the Kaplan-Meier curves, and thus cause-specific proportional hazard models were employed for all outcomes. For CV events and incident diabetes, we visualized cumulative incidence using a Fine-Gray subdistribution hazards model, treating death as a competing risk. A crude unadjusted analysis as well as an adjusted analysis were conducted, accounting for age, sex, and race, as well as smoking status for CV events and mortality.

A stratified analysis was conducted, where the population was divided into strata based on age groups. In each stratum, a separate cause-specific Cox proportional model was utilized, with adjustment to the covariates mentioned earlier. The hazard ratios (HR) and 95% confidence intervals were reported.

A complete case analysis was conducted, and no imputations for missing data were used.

© 2025 Fourman LT et al. *JAMA Network Open*.

eFigure 1. Study Design Timeline Including Covariate Assessment, Baseline, And Follow-Up Windows

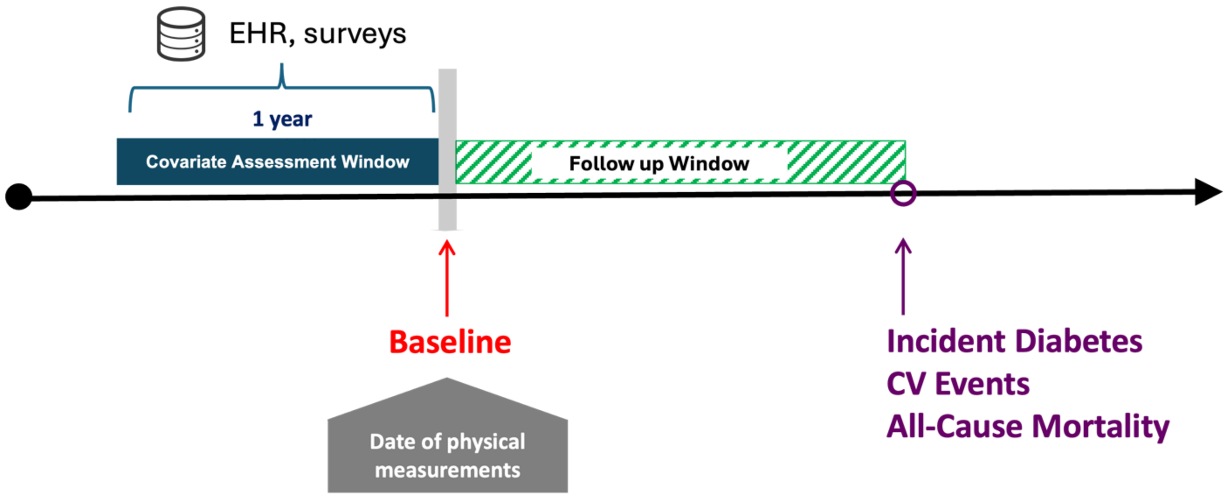

© 2025 Fourman LT et al. *JAMA Network Open*.

eFigure 2. Flow Diagram of the Inclusion/Exclusion Criteria of the Study Analysis

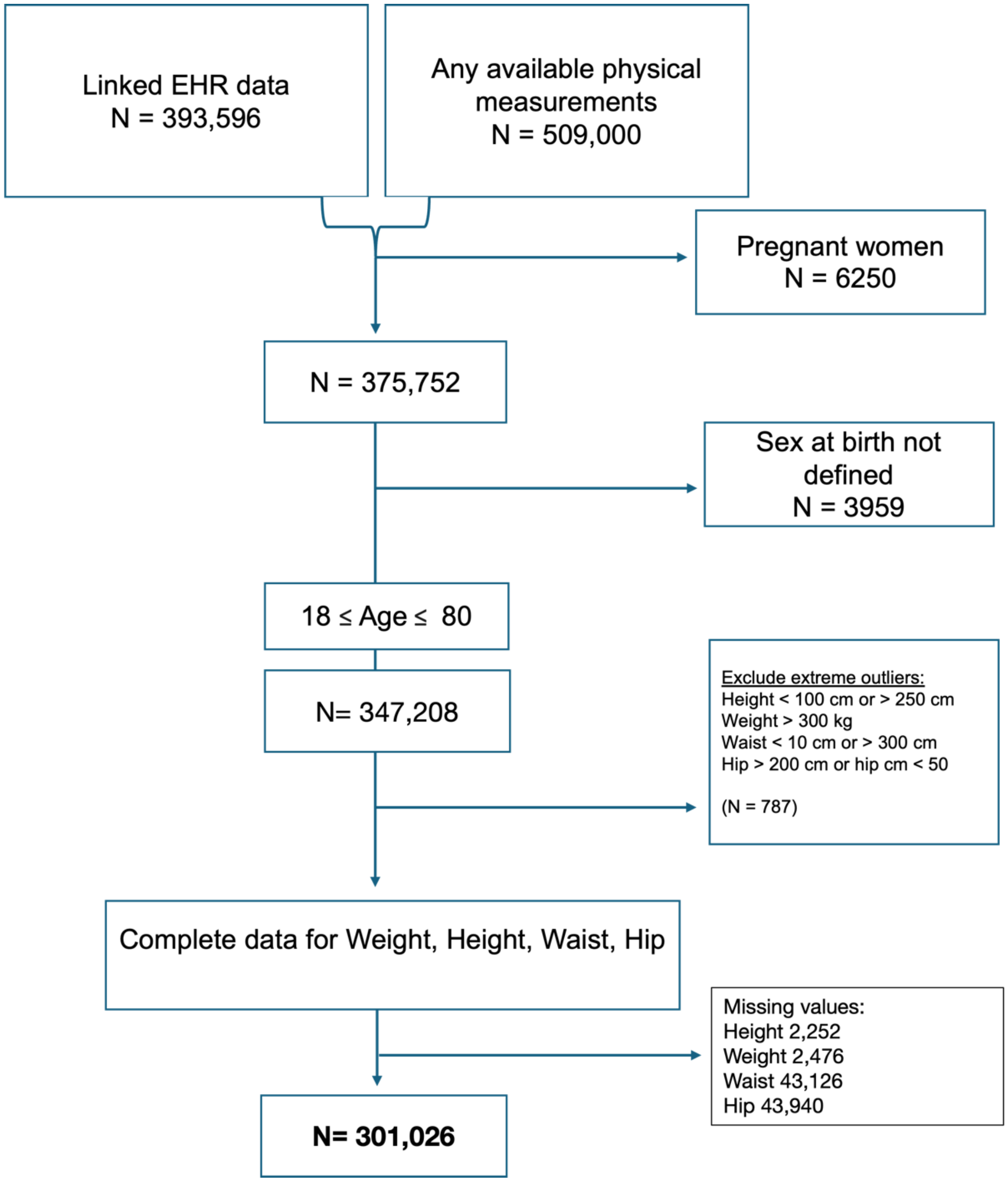

© 2025 Fourman LT et al. *JAMA Network Open*.

eTable 1. Characteristics of Participants Included vs Excluded From the Analysis Due to Incomplete Anthropometric Data

Characteristic	Overall, n (%)	Included in the analysis, n (%)	Excluded from the analysis, n (%)
	(N=347208)	(N=301026)	(N=46182)
**Age, years (median (Q1, Q3))**	54 (39, 65)	54 (38, 65)	55 (39, 67)
**Sex at birth**			
**Male**	135677 (39.1)	117393 (39.0)	18284 (39.6)
**Female**	211531 (60.9)	183633 (61.0)	27898 (60.4)
**Race**			
**American Indian or Alaska Native**	5028 (1.4)	4409 (1.5)	619 (1.3)
**Asian**	10584 (3.0)	9037 (3.0)	1547 (3.3)
**Black or African American**	64239 (18.5)	59347 (19.7)	4892 (10.6)
**Middle Eastern or North African**	2007 (0.6)	1745 (0.6)	262 (0.6)
**White**	188783 (54.4)	160158 (53.2)	28625 (62.0)
**Other[a]**	76567 (22.1)	66330 (22.0)	10237 (22.2)
**Ethnicity**			
**Not Hispanic or Latino**	275825 (79.4)	238933 (79.4)	36892 (79.9)
**Hispanic or Latino**	62293 (17.9)	54308 (18.0)	7985 (17.3)
**Missing**	9090 (2.6)	7785 (2.6)	1305 (2.8)
**BMI, kg/m^2**			
**Median (Q1, Q3)**	28.6 (24.6, 33.9)	28.7 (24.6, 33.9)	28.4 (24.3, 33.9)
**Missing**	3372 (1.0)	0 (0)	3372 (7.3)
**Classification by traditional definition**			
**No Obesity**	197018 (56.7)	172034 (57.1)	24984 (54.1)
**Obesity**	146818 (42.3)	128992 (42.9)	17826 (38.6)
**Missing**	3372 (1.0)	0 (0)	3372 (7.3)

[a]Other group includes Native Hawaiian or Other Pacific Islander (N=353), individuals who chose more than one race (N=15456), individuals who did not identify with any group (N=55226), and individuals who preferred not to answer (N=5532). Continuous variables are reported as median (Q1, Q3). Abbreviations: BMI, body mass index; WHR, waist-hip ratio.

© 2025 Fourman LT et al. *JAMA Network Open*.

eTable 2. Data Sources for the Phenotypic Curation of Manifestations of Organ Dysfunction as Defined by the New Definition

Organ	Condition as defined by *Lancet* Commission	Term	Data sources and definition	ICD concepts[a]	Surveys	
					Survey name	Survey Question
**Central nervous system (CNS)**	Signs of raised intracranial pressure such as vision loss and/or recurrent headaches	Central nervous system (CNS)	Presence of at least one ICD code	Benign intracranial hypertension	-	-
**Upper airways**	Apnoeas/hypopnoeas during sleep due to increased upper airways resistance	Obstructive sleep apnea	ICD OR survey	Obstructive sleep apnea	Personal and overall family history survey	Including yourself, who in your family has had sleep apnea? Select all that apply.<br>• **Self**<br>• Mother<br>• Father<br>• Sibling<br>• Grandparent
**Respiratory**	Hypoventilation and/or breathlessness and/or wheezing due to reduced lung	Hypoventilation	Presence of at least one ICD code	Obesity hypoventilation	-	-

© 2025 Fourman LT et al. *JAMA Network Open*.

Organ	Condition as defined by *Lancet* Commission	Term	Data sources and definition	ICD concepts[a]	Surveys	
	and/or diaphragmatic compliance					
**Cardiovascular (ventricular)**	Reduced Left Ventricular systolic function - Heart Failure with Reduced Ejection Fraction - HFrEF	Heart failure	ICD <u>OR</u> survey	Heart failure	Personal and overall family history survey	Including yourself, who in your family has had congestive heart failure? Select all that apply.<br>• **Self**<br>• Mother<br>• Father<br>• Sibling<br>• Grandparent
**Cardiovascular**	Chronic fatigue, lower limb edema due to impaired diastolic dysfunction– Heart Failure with Preserved Ejection Fraction - HFpEF	Heart failure				
**Cardiovascular (atrial)**	Chronic/recurrent atrial fibrillation	Atrial fibrillation	ICD <u>OR</u> survey	Atrial fibrillation	Personal and overall family history survey	Including yourself, who in your family has had atrial fibrillation (or a-fib) or atrial flutter (or a-flutter)? Select all that apply.<br>• **Self**<br>• Mother<br>• Father<br>• Sibling<br>• Grandparent

© 2025 Fourman LT et al. *JAMA Network Open*.

Organ	Condition as defined by *Lancet* Commission	Term	Data sources and definition	ICD concepts[a]	Surveys	
**Cardiovascular (pulmonary)**	Pulmonary artery hypertension	Pulmonary hypertension	Presence of at least one ICD code	Pulmonary artery hypertension	-	-
**Cardiovascular (thrombosis)**	Recurrent DVT and/or pulmonary thromboembolic disease	Thrombosis	ICD OR survey	• Pulmonary embolism<br>OR<br>• Venous thrombosis	Personal and overall family history survey	Including yourself, who in your family has had pulmonary embolism or deep vein thrombosis (DVT)? Select all that apply.<br>• **Self**<br>• Mother<br>• Father<br>• Sibling<br>• Grandparent
**Cardiovascular (arterial)**	Raised arterial blood pressure	Hypertension	ICD OR survey	Hypertension	Personal and overall family history survey	Including yourself, who in your family has had high blood pressure (hypertension)? Select all that apply.<br>• **Self**<br>• Mother<br>• Father<br>• Sibling<br>• Grandparent

© 2025 Fourman LT et al. *JAMA Network Open*.

Organ	Condition as defined by *Lancet* Commission	Term	Data sources and definition	ICD concepts[a]	Surveys	
**Metabolism**	The cluster of hyperglycaemia, high triglyceride levels, and low HDL cholesterol levels	Metabolic	Hyperglycemia: ICD codes OR survey OR labs (HgA1C >= 5.7, fasting glucose >= 126 mg/dL, glucose >=200 mg/dL).<br>AND<br>Dyslipidemia: ICD codes OR labs (HDL <=40 mg/dL & triglycerides >=150 mg/dL)	• Type 2 diabetes<br>AND<br>• Dyslipidemia	Personal and overall family history survey	Including yourself, who in your family has had type 2 diabetes? Select all that apply.<br>• **Self**<br>• Mother<br>• Father<br>• Sibling
**Liver**	NAFLD with hepatic fibrosis	Liver	ICD OR survey	For ICD codes we required the following:<br>Cirrhosis OR (Hepatic fibrosis & fatty liver & not (viral or alcoholic))	Personal and overall family history survey	Including yourself, who in your family has had a liver condition (e.g., cirrhosis)? Select all that apply.<br>• **Self**<br>• Mother<br>• Father<br>• Sibling

© 2025 Fourman LT et al. *JAMA Network Open*.

Organ	Condition as defined by *Lancet* Commission	Term	Data sources and definition	ICD concepts[a]	Surveys	
**Renal**	Microalbuminuria with reduced eGFR	Renal	Presence of at least one ICD code	• Chronic kidney disease<br>OR<br>• End Stage Kidney Disease	-	-
**Urinary**	Recurrent/chronic urinary incontinence	Urinary	Presence of at least one ICD code	Recurrent/chronic urinary incontinence	-	-
**Reproductive (female)**	Anovulation, oligo-menorrhea and PCOS	Reproductive (female)	ICD OR survey	Female hypogonadism	Personal and overall family history survey	Including yourself, who in your family has had polycystic ovarian syndrome? Select all that apply.<br>• **Self**<br>• Mother<br>• Father<br>• Sibling<br>• Grandparent
**Reproductive (male)**	Male hypogonadism	Reproductive (male)	Presence of at least one ICD code	Male hypogonadism	-	-

© 2025 Fourman LT et al. *JAMA Network Open*.

Organ	Condition as defined by *Lancet* Commission	Term	Data sources and definition	ICD concepts[a]	Surveys	
**Musculoskeletal**	Chronic, severe knee or hip pain associated with joint stiffness and reduced range of joint motion	Musculoskeletal	ICD OR survey	Osteoarthritis	Personal and overall family history survey	Including yourself, who in your family has had osteoarthritis? Select all that apply.<br>• **Self**<br>• Mother<br>• Father<br>• Sibling<br>• Grandparent
**Lymphatic**	Lower limbs lymphedema causing chronic pain and/or reduced range of motion	Lymphedema	ICD AND Survey (pain scale of >= 7)	Lymphedema	Overall health	In the past 7 days, how would you rate your pain on average?<br>Scale of 0 (no pain) - 10 (severe pain)
**Limitations of day-to-day activities**	Significant, age-adjusted limitations of mobility and/or other basic Activities of Daily Living (ADL=bathing, dressing, toileting, continence, eating)	Physical limitation	Surveys<br>An affirmative answer of the following survey item was considered as	-	Overall health	To what extent are you able to carry out your everyday physical activities such as walking, climbing stairs, carrying groceries, or moving a chair?

© 2025 Fourman LT et al. *JAMA Network Open*.

Organ	Condition as defined by *Lancet* Commission	Term	Data sources and definition	ICD concepts[a]	Surveys	
			having physical limitation			• Completely<br>• Mostly<br>• Moderately<br>• **A little**<br>• **Not at all**<br><br>Serious limitation was defined as: a little OR not at all.
					The basics	Do you have serious difficulty walking or climbing stairs?<br>• **Yes**<br>• No<br>• Prefer not to say<br><br>Do you have difficulty dressing or bathing?<br>• **Yes**<br>• No<br>• Prefer not to say

[a]The ICD concepts map to **eTable 3**, with all the ICDs related to that concept.

© 2025 Fourman LT et al. *JAMA Network Open*.

eTable 3. *ICD* Concepts Mapped to the Organ Dysfunction Categories

Organ	ICD concept	ICD code	ICD description	ICD year	Count
**Central nervous system (CNS)**	Benign intracranial hypertension	G93.2	Benign intracranial hypertension	ICD10CM	18719
**Upper airways**	Obstructive sleep apnea	G47.33	Obstructive sleep apnea (adult) (pediatric)	ICD10CM	708772
**Respiratory**	Obesity hypoventilation	E66.2	Morbid (severe) obesity with alveolar hypoventilation	ICD10CM	9741
**Cardiovascular (ventricular)**	Heart Failure	428	Heart failure	ICD9CM	180
		428	Congestive heart failure, unspecified	ICD9CM	86501
		428.1	Left heart failure	ICD9CM	5271
		428.2	Systolic heart failure	ICD9CM	5
		428.2	Systolic heart failure, unspecified	ICD9CM	6715
		428.22	Chronic systolic heart failure	ICD9CM	15666
		428.23	Acute on chronic systolic heart failure	ICD9CM	4179
		428.3	Diastolic heart failure	ICD9CM	133
		428.3	Diastolic heart failure, unspecified	ICD9CM	5910
		428.32	Chronic diastolic heart failure	ICD9CM	11109
		428.33	Acute on chronic diastolic heart failure	ICD9CM	3100
		428.4	Combined systolic and diastolic heart failure, unspecified	ICD9CM	1040
		428.42	Chronic combined systolic and diastolic heart failure	ICD9CM	3765
		428.43	Acute on chronic combined systolic and diastolic heart failure	ICD9CM	1761
		428.9	Heart failure, unspecified	ICD9CM	13033
		I50	Heart failure	ICD10CM	63
		I50.1	Left ventricular failure, unspecified	ICD10CM	4991
		I50.2	Systolic (congestive) heart failure	ICD10CM	12

© 2025 Fourman LT et al. *JAMA Network Open*.

Organ	ICD concept	ICD code	ICD description	ICD year	Count
		I50.20	Unspecified systolic (congestive) heart failure	ICD10CM	84436
		I50.22	Chronic systolic (congestive) heart failure	ICD10CM	185863
		I50.23	Acute on chronic systolic (congestive) heart failure	ICD10CM	52193
		I50.3	Diastolic (congestive) heart failure	ICD10CM	89
		I50.30	Unspecified diastolic (congestive) heart failure	ICD10CM	97727
		I50.32	Chronic diastolic (congestive) heart failure	ICD10CM	171946
		I50.33	Acute on chronic diastolic (congestive) heart failure	ICD10CM	58363
		I50.40	Unspecified combined systolic (congestive) and diastolic (congestive) heart failure	ICD10CM	6940
		I50.42	Chronic combined systolic (congestive) and diastolic (congestive) heart failure	ICD10CM	44246
		I50.814	Right heart failure due to left heart failure	ICD10CM	166
		I50.82	Biventricular heart failure	ICD10CM	2577
		I50.84	End stage heart failure	ICD10CM	1442
		I50.9	Heart failure, unspecified	ICD10CM	171385
**Cardiovascular (atrial)**	Atrial fibrillation	427.3	Atrial fibrillation and flutter	ICD9CM	301
		427.31	Atrial fibrillation	ICD9CM	238900
		427.32	Atrial flutter	ICD9CM	24019
		I48	Atrial fibrillation and flutter	ICD10CM	338
		I48.0	Paroxysmal atrial fibrillation	ICD10CM	283865
		I48.1	Persistent atrial fibrillation	ICD10CM	17796
		I48.11	Longstanding persistent atrial fibrillation	ICD10CM	6008
		I48.19	Other persistent atrial fibrillation	ICD10CM	32295

© 2025 Fourman LT et al. *JAMA Network Open*.

Organ	ICD concept	ICD code	ICD description	ICD year	Count
		I48.2	Chronic atrial fibrillation	ICD10CM	29125
		I48.20	Chronic atrial fibrillation, unspecified	ICD10CM	37604
		I48.21	Permanent atrial fibrillation	ICD10CM	8926
		I48.3	Typical atrial flutter	ICD10CM	13907
		I48.4	Atypical atrial flutter	ICD10CM	7331
		I48.9	Unspecified atrial fibrillation and atrial flutter	ICD10CM	1
		I48.91	Unspecified atrial fibrillation	ICD10CM	306611
		I48.92	Unspecified atrial flutter	ICD10CM	54803
		427.3	Atrial fibrillation and flutter	ICD9CM	301
		427.31	Atrial fibrillation	ICD9CM	238900
		427.32	Atrial flutter	ICD9CM	24019
		I48	Atrial fibrillation and flutter	ICD10CM	338
		I48.0	Paroxysmal atrial fibrillation	ICD10CM	283865
		I48.1	Persistent atrial fibrillation	ICD10CM	17796
		I48.11	Longstanding persistent atrial fibrillation	ICD10CM	6008
		I48.19	Other persistent atrial fibrillation	ICD10CM	32295
		I48.2	Chronic atrial fibrillation	ICD10CM	29125
		I48.20	Chronic atrial fibrillation, unspecified	ICD10CM	37604
		I48.21	Permanent atrial fibrillation	ICD10CM	8926
		I48.3	Typical atrial flutter	ICD10CM	13907
		I48.4	Atypical atrial flutter	ICD10CM	7331
		I48.9	Unspecified atrial fibrillation and atrial flutter	ICD10CM	1
		I48.91	Unspecified atrial fibrillation	ICD10CM	306611
		I48.92	Unspecified atrial flutter	ICD10CM	54803
**Cardiovascular (pulmonary)**	Pulmonary artery hypertension	416	Primary pulmonary hypertension	ICD9CM	5846

© 2025 Fourman LT et al. *JAMA Network Open*.

Organ	ICD concept	ICD code	ICD description	ICD year	Count
		I27.0	Primary pulmonary hypertension	ICD10CM	8440
		I27.2	Other secondary pulmonary hypertension	ICD10CM	10860
		I27.20	Pulmonary hypertension, unspecified	ICD10CM	60826
		I27.21	Secondary pulmonary arterial hypertension	ICD10CM	7889
		I27.22	Pulmonary hypertension due to left heart disease	ICD10CM	2708
		I27.24	Chronic thromboembolic pulmonary hypertension	ICD10CM	2282
		I27.29	Other secondary pulmonary hypertension	ICD10CM	3754
		416	Primary pulmonary hypertension	ICD9CM	5846
		I27.0	Primary pulmonary hypertension	ICD10CM	8440
		I27.2	Other secondary pulmonary hypertension	ICD10CM	10860
		I27.20	Pulmonary hypertension, unspecified	ICD10CM	60826
		I27.21	Secondary pulmonary arterial hypertension	ICD10CM	7889
		I27.22	Pulmonary hypertension due to left heart disease	ICD10CM	2708
		I27.24	Chronic thromboembolic pulmonary hypertension	ICD10CM	2282
		I27.29	Other secondary pulmonary hypertension	ICD10CM	3754
**Cardiovascular (thrombosis)**	Pulmonary embolism	415.1	Pulmonary embolism and infarction	ICD9CM	691
		415.13	Saddle embolus of pulmonary artery	ICD9CM	666
		415.19	Other pulmonary embolism and infarction	ICD9CM	62903
		416.2	Chronic pulmonary embolism	ICD9CM	1630
		I26	Pulmonary embolism	ICD10CM	295
		I26.02	Saddle embolus of pulmonary artery with acute cor pulmonale	ICD10CM	1410

© 2025 Fourman LT et al. *JAMA Network Open*.

Organ	ICD concept	ICD code	ICD description	ICD year	Count
		I26.09	Other pulmonary embolism with acute cor pulmonale	ICD10CM	6026
		I26.92	Saddle embolus of pulmonary artery without acute cor pulmonale	ICD10CM	2578
		I26.93	Single subsegmental pulmonary embolism without acute cor pulmonale	ICD10CM	1517
		I26.94	Multiple subsegmental pulmonary emboli without acute cor pulmonale	ICD10CM	1705
		I26.99	Other pulmonary embolism without acute cor pulmonale	ICD10CM	112004
		I27.82	Chronic pulmonary embolism	ICD10CM	9689
	Venous thrombosis	453.72	Chronic venous embolism and thrombosis of deep veins of upper extremity	ICD9CM	271
		I82.501	Chronic embolism and thrombosis of unspecified deep veins of right lower extremity	ICD10CM	995
		I82.502	Chronic embolism and thrombosis of unspecified deep veins of left lower extremity	ICD10CM	1396
		I82.503	Chronic embolism and thrombosis of unspecified deep veins of lower extremity, bilateral	ICD10CM	1002
		I82.509	Chronic embolism and thrombosis of unspecified deep veins of unspecified lower extremity	ICD10CM	2626
		I82.511	Chronic embolism and thrombosis of right femoral vein	ICD10CM	2065
		I82.512	Chronic embolism and thrombosis of left femoral vein	ICD10CM	2586
		I82.513	Chronic embolism and thrombosis of femoral vein, bilateral	ICD10CM	785
		I82.519	Chronic embolism and thrombosis of unspecified femoral vein	ICD10CM	264
		I82.521	Chronic embolism and thrombosis of right iliac vein	ICD10CM	184

© 2025 Fourman LT et al. *JAMA Network Open*.

Organ	ICD concept	ICD code	ICD description	ICD year	Count
		I82.522	Chronic embolism and thrombosis of left iliac vein	ICD10CM	374
		I82.523	Chronic embolism and thrombosis of iliac vein, bilateral	ICD10CM	108
		I82.529	Chronic embolism and thrombosis of unspecified iliac vein	ICD10CM	45
		I82.531	Chronic embolism and thrombosis of right popliteal vein	ICD10CM	956
		I82.532	Chronic embolism and thrombosis of left popliteal vein	ICD10CM	1509
		I82.533	Chronic embolism and thrombosis of popliteal vein, bilateral	ICD10CM	258
		I82.539	Chronic embolism and thrombosis of unspecified popliteal vein	ICD10CM	217
		I82.541	Chronic embolism and thrombosis of right tibial vein	ICD10CM	220
		I82.542	Chronic embolism and thrombosis of left tibial vein	ICD10CM	181
		I82.543	Chronic embolism and thrombosis of tibial vein, bilateral	ICD10CM	55
		I82.549	Chronic embolism and thrombosis of unspecified tibial vein	ICD10CM	53
		I82.551	Chronic embolism and thrombosis of right peroneal vein	ICD10CM	138
		I82.552	Chronic embolism and thrombosis of left peroneal vein	ICD10CM	82
		I82.553	Chronic embolism and thrombosis of peroneal vein, bilateral	ICD10CM	42
		I82.559	Chronic embolism and thrombosis of unspecified peroneal vein	ICD10CM	7
		I82.561	Chronic embolism and thrombosis of right calf muscular vein	ICD10CM	180
		I82.562	Chronic embolism and thrombosis of left calf muscular vein	ICD10CM	184
		I82.563	Chronic embolism and thrombosis of calf muscular vein, bilateral	ICD10CM	66
		I82.569	Chronic embolism and thrombosis of unspecified calf muscular vein	ICD10CM	44

© 2025 Fourman LT et al. *JAMA Network Open*.

Organ	ICD concept	ICD code	ICD description	ICD year	Count
		I82.591	Chronic embolism and thrombosis of other specified deep vein of right lower extremity	ICD10CM	293
		I82.592	Chronic embolism and thrombosis of other specified deep vein of left lower extremity	ICD10CM	491
		I82.593	Chronic embolism and thrombosis of other specified deep vein of lower extremity, bilateral	ICD10CM	446
		I82.599	Chronic embolism and thrombosis of other specified deep vein of unspecified lower extremity	ICD10CM	391
		I82.5Y1	Chronic embolism and thrombosis of unspecified deep veins of right proximal lower extremity	ICD10CM	413
		I82.5Y2	Chronic embolism and thrombosis of unspecified deep veins of left proximal lower extremity	ICD10CM	839
		I82.5Y3	Chronic embolism and thrombosis of unspecified deep veins of proximal lower extremity, bilateral	ICD10CM	431
		I82.5Y9	Chronic embolism and thrombosis of unspecified deep veins of unspecified proximal lower extremity	ICD10CM	810
		I82.5Z1	Chronic embolism and thrombosis of unspecified deep veins of right distal lower extremity	ICD10CM	785
		I82.5Z2	Chronic embolism and thrombosis of unspecified deep veins of left distal lower extremity	ICD10CM	1008
		I82.5Z3	Chronic embolism and thrombosis of unspecified deep veins of distal lower extremity, bilateral	ICD10CM	210
		I82.5Z9	Chronic embolism and thrombosis of unspecified deep veins of unspecified distal lower extremity	ICD10CM	863
		I82.721	Chronic embolism and thrombosis of deep veins of right upper extremity	ICD10CM	354

© 2025 Fourman LT et al. *JAMA Network Open*.

Organ	ICD concept	ICD code	ICD description	ICD year	Count
		I82.722	Chronic embolism and thrombosis of deep veins of left upper extremity	ICD10CM	647
		I82.723	Chronic embolism and thrombosis of deep veins of upper extremity, bilateral	ICD10CM	154
		I82.729	Chronic embolism and thrombosis of deep veins of unspecified upper extremity	ICD10CM	404
**Cardiovascular (arterial)**	Hypertension	401	Essential hypertension	ICD9CM	623
		401.1	Benign essential hypertension	ICD9CM	295648
		401.9	Unspecified essential hypertension	ICD9CM	1356697
		I10	Essential (primary) hypertension	ICD10CM	3081719
**Metabolism**	Type 2 diabetes	E11	Type 2 diabetes mellitus	ICD10CM	1000
		E11.00	Type 2 diabetes mellitus with hyperosmolarity without nonketotic hyperglycemic-hyperosmolar coma (NKHHC)	ICD10CM	15906
		E11.01	Type 2 diabetes mellitus with hyperosmolarity with coma	ICD10CM	645
		E11.10	Type 2 diabetes mellitus with ketoacidosis without coma	ICD10CM	11898
		E11.11	Type 2 diabetes mellitus with ketoacidosis with coma	ICD10CM	90
		E11.2	Type 2 diabetes mellitus with kidney complications	ICD10CM	2
		E11.21	Type 2 diabetes mellitus with diabetic nephropathy	ICD10CM	45559
		E11.22	Type 2 diabetes mellitus with diabetic chronic kidney disease	ICD10CM	268087
		E11.29	Type 2 diabetes mellitus with other diabetic kidney complication	ICD10CM	39308
		E11.3	Type 2 diabetes mellitus with ophthalmic complications	ICD10CM	9
		E11.31	Type 2 diabetes mellitus with unspecified diabetic retinopathy	ICD10CM	3

© 2025 Fourman LT et al. *JAMA Network Open*.

Organ	ICD concept	ICD code	ICD description	ICD year	Count
		E11.311	Type 2 diabetes mellitus with unspecified diabetic retinopathy with macular edema	ICD10CM	6635
		E11.319	Type 2 diabetes mellitus with unspecified diabetic retinopathy without macular edema	ICD10CM	23731
		E11.321	Type 2 diabetes mellitus with mild nonproliferative diabetic retinopathy with macular edema	ICD10CM	524
		E11.3211	Type 2 diabetes mellitus with mild nonproliferative diabetic retinopathy with macular edema, right eye	ICD10CM	1918
		E11.3212	Type 2 diabetes mellitus with mild nonproliferative diabetic retinopathy with macular edema, left eye	ICD10CM	1258
		E11.3213	Type 2 diabetes mellitus with mild nonproliferative diabetic retinopathy with macular edema, bilateral	ICD10CM	3871
		E11.3219	Type 2 diabetes mellitus with mild nonproliferative diabetic retinopathy with macular edema, unspecified eye	ICD10CM	975
		E11.329	Type 2 diabetes mellitus with mild nonproliferative diabetic retinopathy without macular edema	ICD10CM	1125
		E11.3291	Type 2 diabetes mellitus with mild nonproliferative diabetic retinopathy without macular edema, right eye	ICD10CM	2032
		E11.3292	Type 2 diabetes mellitus with mild nonproliferative diabetic retinopathy without macular edema, left eye	ICD10CM	2087
		E11.3293	Type 2 diabetes mellitus with mild nonproliferative diabetic retinopathy without macular edema, bilateral	ICD10CM	10536
		E11.3299	Type 2 diabetes mellitus with mild nonproliferative diabetic retinopathy without macular edema, unspecified eye	ICD10CM	10307

© 2025 Fourman LT et al. *JAMA Network Open*.

Organ	ICD concept	ICD code	ICD description	ICD year	Count
		E11.331	Type 2 diabetes mellitus with moderate nonproliferative diabetic retinopathy with macular edema	ICD10CM	440
		E11.3311	Type 2 diabetes mellitus with moderate nonproliferative diabetic retinopathy with macular edema, right eye	ICD10CM	2340
		E11.3312	Type 2 diabetes mellitus with moderate nonproliferative diabetic retinopathy with macular edema, left eye	ICD10CM	1994
		E11.3313	Type 2 diabetes mellitus with moderate nonproliferative diabetic retinopathy with macular edema, bilateral	ICD10CM	6342
		E11.3319	Type 2 diabetes mellitus with moderate nonproliferative diabetic retinopathy with macular edema, unspecified eye	ICD10CM	500
		E11.339	Type 2 diabetes mellitus with moderate nonproliferative diabetic retinopathy without macular edema	ICD10CM	305
		E11.3391	Type 2 diabetes mellitus with moderate nonproliferative diabetic retinopathy without macular edema, right eye	ICD10CM	667
		E11.3392	Type 2 diabetes mellitus with moderate nonproliferative diabetic retinopathy without macular edema, left eye	ICD10CM	612
		E11.3393	Type 2 diabetes mellitus with moderate nonproliferative diabetic retinopathy without macular edema, bilateral	ICD10CM	2678
		E11.3399	Type 2 diabetes mellitus with moderate nonproliferative diabetic retinopathy without macular edema, unspecified eye	ICD10CM	722

© 2025 Fourman LT et al. *JAMA Network Open*.

Organ	ICD concept	ICD code	ICD description	ICD year	Count
		E11.341	Type 2 diabetes mellitus with severe nonproliferative diabetic retinopathy with macular edema	ICD10CM	304
		E11.3411	Type 2 diabetes mellitus with severe nonproliferative diabetic retinopathy with macular edema, right eye	ICD10CM	1166
		E11.3412	Type 2 diabetes mellitus with severe nonproliferative diabetic retinopathy with macular edema, left eye	ICD10CM	1004
		E11.3413	Type 2 diabetes mellitus with severe nonproliferative diabetic retinopathy with macular edema, bilateral	ICD10CM	2650
		E11.3419	Type 2 diabetes mellitus with severe nonproliferative diabetic retinopathy with macular edema, unspecified eye	ICD10CM	383
		E11.349	Type 2 diabetes mellitus with severe nonproliferative diabetic retinopathy without macular edema	ICD10CM	160
		E11.3491	Type 2 diabetes mellitus with severe nonproliferative diabetic retinopathy without macular edema, right eye	ICD10CM	369
		E11.3492	Type 2 diabetes mellitus with severe nonproliferative diabetic retinopathy without macular edema, left eye	ICD10CM	290
		E11.3493	Type 2 diabetes mellitus with severe nonproliferative diabetic retinopathy without macular edema, bilateral	ICD10CM	882
		E11.3499	Type 2 diabetes mellitus with severe nonproliferative diabetic retinopathy without macular edema, unspecified eye	ICD10CM	283
		E11.35	Type 2 diabetes mellitus with proliferative diabetic retinopathy	ICD10CM	3
		E11.351	Type 2 diabetes mellitus with proliferative diabetic retinopathy with macular edema	ICD10CM	937

© 2025 Fourman LT et al. *JAMA Network Open*.

Organ	ICD concept	ICD code	ICD description	ICD year	Count
		E11.3511	Type 2 diabetes mellitus with proliferative diabetic retinopathy with macular edema, right eye	ICD10CM	3198
		E11.3512	Type 2 diabetes mellitus with proliferative diabetic retinopathy with macular edema, left eye	ICD10CM	2444
		E11.3513	Type 2 diabetes mellitus with proliferative diabetic retinopathy with macular edema, bilateral	ICD10CM	11040
		E11.3519	Type 2 diabetes mellitus with proliferative diabetic retinopathy with macular edema, unspecified eye	ICD10CM	1231
		E11.3521	Type 2 diabetes mellitus with proliferative diabetic retinopathy with traction retinal detachment involving the macula, right eye	ICD10CM	440
		E11.3522	Type 2 diabetes mellitus with proliferative diabetic retinopathy with traction retinal detachment involving the macula, left eye	ICD10CM	422
		E11.3523	Type 2 diabetes mellitus with proliferative diabetic retinopathy with traction retinal detachment involving the macula, bilateral	ICD10CM	914
		E11.3529	Type 2 diabetes mellitus with proliferative diabetic retinopathy with traction retinal detachment involving the macula, unspecified eye	ICD10CM	21
		E11.3531	Type 2 diabetes mellitus with proliferative diabetic retinopathy with traction retinal detachment not involving the macula, right eye	ICD10CM	315
		E11.3532	Type 2 diabetes mellitus with proliferative diabetic retinopathy with traction retinal detachment not involving the macula, left eye	ICD10CM	271
		E11.3533	Type 2 diabetes mellitus with proliferative diabetic retinopathy with	ICD10CM	156

© 2025 Fourman LT et al. *JAMA Network Open*.

Organ	ICD concept	ICD code	ICD description	ICD year	Count
			traction retinal detachment not involving the macula, bilateral		
		E11.3539	Type 2 diabetes mellitus with proliferative diabetic retinopathy with traction retinal detachment not involving the macula, unspecified eye	ICD10CM	16
		E11.3541	Type 2 diabetes mellitus with proliferative diabetic retinopathy with combined traction retinal detachment and rhegmatogenous retinal detachment, right eye	ICD10CM	115
		E11.3542	Type 2 diabetes mellitus with proliferative diabetic retinopathy with combined traction retinal detachment and rhegmatogenous retinal detachment, left eye	ICD10CM	110
		E11.3543	Type 2 diabetes mellitus with proliferative diabetic retinopathy with combined traction retinal detachment and rhegmatogenous retinal detachment, bilateral	ICD10CM	24
		E11.3549	Type 2 diabetes mellitus with proliferative diabetic retinopathy with combined traction retinal detachment and rhegmatogenous retinal detachment, unspecified eye	ICD10CM	57
		E11.3551	Type 2 diabetes mellitus with stable proliferative diabetic retinopathy, right eye	ICD10CM	396
		E11.3552	Type 2 diabetes mellitus with stable proliferative diabetic retinopathy, left eye	ICD10CM	390
		E11.3553	Type 2 diabetes mellitus with stable proliferative diabetic retinopathy, bilateral	ICD10CM	2081

© 2025 Fourman LT et al. *JAMA Network Open*.

Organ	ICD concept	ICD code	ICD description	ICD year	Count
		E11.3559	Type 2 diabetes mellitus with stable proliferative diabetic retinopathy, unspecified eye	ICD10CM	156
		E11.359	Type 2 diabetes mellitus with proliferative diabetic retinopathy without macular edema	ICD10CM	1095
		E11.3591	Type 2 diabetes mellitus with proliferative diabetic retinopathy without macular edema, right eye	ICD10CM	1100
		E11.3592	Type 2 diabetes mellitus with proliferative diabetic retinopathy without macular edema, left eye	ICD10CM	1623
		E11.3593	Type 2 diabetes mellitus with proliferative diabetic retinopathy without macular edema, bilateral	ICD10CM	5858
		E11.3599	Type 2 diabetes mellitus with proliferative diabetic retinopathy without macular edema, unspecified eye	ICD10CM	4213
		E11.36	Type 2 diabetes mellitus with diabetic cataract	ICD10CM	8837
		E11.37X1	Type 2 diabetes mellitus with diabetic macular edema, resolved following treatment, right eye	ICD10CM	12
		E11.37X2	Type 2 diabetes mellitus with diabetic macular edema, resolved following treatment, left eye	ICD10CM	23
		E11.37X3	Type 2 diabetes mellitus with diabetic macular edema, resolved following treatment, bilateral	ICD10CM	229
		E11.37X9	Type 2 diabetes mellitus with diabetic macular edema, resolved following treatment, unspecified eye	ICD10CM	45
		E11.39	Type 2 diabetes mellitus with other diabetic ophthalmic complication	ICD10CM	8290
		E11.4	Type 2 diabetes mellitus with neurological complications	ICD10CM	6

© 2025 Fourman LT et al. *JAMA Network Open*.

Organ	ICD concept	ICD code	ICD description	ICD year	Count
		E11.40	Type 2 diabetes mellitus with diabetic neuropathy, unspecified	ICD10CM	120637
		E11.41	Type 2 diabetes mellitus with diabetic mononeuropathy	ICD10CM	3250
		E11.42	Type 2 diabetes mellitus with diabetic polyneuropathy	ICD10CM	140607
		E11.43	Type 2 diabetes mellitus with diabetic autonomic (poly)neuropathy	ICD10CM	18965
		E11.44	Type 2 diabetes mellitus with diabetic amyotrophy	ICD10CM	386
		E11.49	Type 2 diabetes mellitus with other diabetic neurological complication	ICD10CM	25585
		E11.5	Type 2 diabetes mellitus with circulatory complications	ICD10CM	1
		E11.51	Type 2 diabetes mellitus with diabetic peripheral angiopathy without gangrene	ICD10CM	33534
		E11.52	Type 2 diabetes mellitus with diabetic peripheral angiopathy with gangrene	ICD10CM	8100
		E11.59	Type 2 diabetes mellitus with other circulatory complications	ICD10CM	39805
		E11.610	Type 2 diabetes mellitus with diabetic neuropathic arthropathy	ICD10CM	12396
		E11.618	Type 2 diabetes mellitus with other diabetic arthropathy	ICD10CM	888
		E11.62	Type 2 diabetes mellitus with skin complications	ICD10CM	2
		E11.620	Type 2 diabetes mellitus with diabetic dermatitis	ICD10CM	1113
		E11.621	Type 2 diabetes mellitus with foot ulcer	ICD10CM	80703
		E11.622	Type 2 diabetes mellitus with other skin ulcer	ICD10CM	5929
		E11.628	Type 2 diabetes mellitus with other skin complications	ICD10CM	9971
		E11.630	Type 2 diabetes mellitus with periodontal disease	ICD10CM	258

© 2025 Fourman LT et al. *JAMA Network Open*.

Organ	ICD concept	ICD code	ICD description	ICD year	Count
		E11.638	Type 2 diabetes mellitus with other oral complications	ICD10CM	516
		E11.64	Type 2 diabetes mellitus with hypoglycemia	ICD10CM	17
		E11.641	Type 2 diabetes mellitus with hypoglycemia with coma	ICD10CM	260
		E11.649	Type 2 diabetes mellitus with hypoglycemia without coma	ICD10CM	27201
		E11.65	Type 2 diabetes mellitus with hyperglycemia	ICD10CM	545819
		E11.69	Type 2 diabetes mellitus with other specified complication	ICD10CM	160693
		E11.8	Type 2 diabetes mellitus with unspecified complications	ICD10CM	168045
		E11.9	Type 2 diabetes mellitus without complications	ICD10CM	1264199
	Dyslipidemia	E78	Disorders of lipoprotein metabolism and other lipidemias	ICD10CM	3337
		E78.2	Mixed hyperlipidemia	ICD10CM	373901
		E78.5	Hyperlipidemia, unspecified	ICD10CM	1309554
**Liver**	Fatty liver	K75.81	Nonalcoholic steatohepatitis (NASH)	ICD10CM	38141
		K76.0	Fatty (change of) liver, not elsewhere classified	ICD10CM	145454
	Cirrhosis	571	Chronic liver disease and cirrhosis	ICD9CM	15
		571.5	Cirrhosis of liver without mention of alcohol	ICD9CM	39092
		K74	Fibrosis and cirrhosis of liver	ICD10CM	51
		K74.60	Unspecified cirrhosis of liver	ICD10CM	87532
		K74.69	Other cirrhosis of liver	ICD10CM	18480
	Hepatic fibrosis	K74.0	Hepatic fibrosis	ICD10CM	2522
		K74.00	Hepatic fibrosis, unspecified	ICD10CM	3654
		K74.01	Hepatic fibrosis, early fibrosis	ICD10CM	198
		K74.02	Hepatic fibrosis, advanced fibrosis	ICD10CM	359

© 2025 Fourman LT et al. *JAMA Network Open*.

Organ	ICD concept	ICD code	ICD description	ICD year	Count
		K74.2	Hepatic fibrosis with hepatic sclerosis	ICD10CM	36
	Viral liver disease (to exclude)	70.22	Chronic viral hepatitis B with hepatic coma without hepatitis delta	ICD9CM	58
		70.23	Chronic viral hepatitis B with hepatic coma with hepatitis delta	ICD9CM	9
		70.32	Chronic viral hepatitis B without mention of hepatic coma without mention of hepatitis delta	ICD9CM	12347
		70.33	Chronic viral hepatitis B without mention of hepatic coma with hepatitis delta	ICD9CM	344
		70.44	Chronic hepatitis C with hepatic coma	ICD9CM	1841
		70.54	Chronic hepatitis C without mention of hepatic coma	ICD9CM	55042
		571.4	Chronic hepatitis	ICD9CM	33
		571.4	Chronic hepatitis, unspecified	ICD9CM	3049
		571.41	Chronic persistent hepatitis	ICD9CM	119
		571.42	Autoimmune hepatitis	ICD9CM	5918
		571.49	Other chronic hepatitis	ICD9CM	1493
		B18.0	Chronic viral hepatitis B with delta-agent	ICD10CM	240
		B18.1	Chronic viral hepatitis B without delta-agent	ICD10CM	16553
		B18.2	Chronic viral hepatitis C	ICD10CM	68150
		B18.8	Other chronic viral hepatitis	ICD10CM	77
		B18.9	Chronic viral hepatitis, unspecified	ICD10CM	136
		K73	Chronic hepatitis, not elsewhere classified	ICD10CM	1
		K73.0	Chronic persistent hepatitis, not elsewhere classified	ICD10CM	34
		K73.1	Chronic lobular hepatitis, not elsewhere classified	ICD10CM	4

© 2025 Fourman LT et al. *JAMA Network Open*.

Organ	ICD concept	ICD code	ICD description	ICD year	Count
		K73.2	Chronic active hepatitis, not elsewhere classified	ICD10CM	277
		K73.8	Other chronic hepatitis, not elsewhere classified	ICD10CM	326
		K73.9	Chronic hepatitis, unspecified	ICD10CM	1865
	Alcoholic liver disease (to exclude)	571	Alcoholic fatty liver	ICD9CM	540
		571.2	Alcoholic cirrhosis of liver	ICD9CM	7860
		571.3	Alcoholic liver damage, unspecified	ICD9CM	1274
		K70	Alcoholic liver disease	ICD10CM	4
		K70.0	Alcoholic fatty liver	ICD10CM	1629
		K70.1	Alcoholic hepatitis	ICD10CM	18
		K70.10	Alcoholic hepatitis without ascites	ICD10CM	4533
		K70.11	Alcoholic hepatitis with ascites	ICD10CM	1546
		K70.2	Alcoholic fibrosis and sclerosis of liver	ICD10CM	50
		K70.3	Alcoholic cirrhosis of liver	ICD10CM	20
		K70.30	Alcoholic cirrhosis of liver without ascites	ICD10CM	19466
		K70.31	Alcoholic cirrhosis of liver with ascites	ICD10CM	16984
		K70.40	Alcoholic hepatic failure without coma	ICD10CM	816
		K70.41	Alcoholic hepatic failure with coma	ICD10CM	8
		K70.9	Alcoholic liver disease, unspecified	ICD10CM	3490
**Renal**	Chronic Kidney disease	585	Chronic kidney disease (CKD)	ICD9CM	6473
		585.1	Chronic kidney disease, Stage I	ICD9CM	4681
		585.2	Chronic kidney disease, Stage II (mild)	ICD9CM	13501
		585.3	Chronic kidney disease, Stage III (moderate)	ICD9CM	71344

© 2025 Fourman LT et al. *JAMA Network Open*.

Organ	ICD concept	ICD code	ICD description	ICD year	Count
		585.4	Chronic kidney disease, Stage IV (severe)	ICD9CM	22269
		585.5	Chronic kidney disease, Stage V	ICD9CM	8594
		585.9	Chronic kidney disease, unspecified	ICD9CM	61396
		N18	Chronic kidney disease (CKD)	ICD10CM	156
		N18.1	Chronic kidney disease, stage 1	ICD10CM	9502
		N18.2	Chronic kidney disease, stage 2 (mild)	ICD10CM	45762
		N18.3	Chronic kidney disease, stage 3 (moderate)	ICD10CM	129056
		N18.30	Chronic kidney disease, stage 3 unspecified	ICD10CM	117175
		N18.31	Chronic kidney disease, stage 3a	ICD10CM	44399
		N18.32	Chronic kidney disease, stage 3b	ICD10CM	34256
		N18.4	Chronic kidney disease, stage 4 (severe)	ICD10CM	91341
		N18.5	Chronic kidney disease, stage 5	ICD10CM	35308
		N18.9	Chronic kidney disease, unspecified	ICD10CM	144300
	End stage renal disease	585.6	End stage renal disease	ICD9CM	62841
		N18.6	End stage renal disease	ICD10CM	227934
**Urinary**	Recurrent/chronic urinary incontinence	788.3	Urinary incontinence	ICD9CM	1672
		788.3	Urinary incontinence, unspecified	ICD9CM	34153
		788.31	Urge incontinence	ICD9CM	22908
		788.32	Stress incontinence, male	ICD9CM	2052
		788.33	Mixed incontinence (male) (female)	ICD9CM	13481
		788.35	Post-void dribbling	ICD9CM	896
		788.37	Continuous leakage	ICD9CM	458
		788.38	Overflow incontinence	ICD9CM	222

© 2025 Fourman LT et al. *JAMA Network Open*.

Organ	ICD concept	ICD code	ICD description	ICD year	Count
		788.39	Other urinary incontinence	ICD9CM	1672
		N39.4	Other specified urinary incontinence	ICD10CM	1
		N39.41	Urge incontinence	ICD10CM	59353
		N39.42	Incontinence without sensory awareness	ICD10CM	2228
		N39.45	Continuous leakage	ICD10CM	1552
		N39.46	Mixed incontinence	ICD10CM	41410
		N39.490	Overflow incontinence	ICD10CM	1320
		N39.492	Postural (urinary) incontinence	ICD10CM	134
		N39.498	Other specified urinary incontinence	ICD10CM	3515
		R32	Unspecified urinary incontinence	ICD10CM	60014
**Reproductive (female)**	Female hypogonadism	628	Infertility, female	ICD9CM	10
		628	Infertility, female, associated with anovulation	ICD9CM	1903
		628.1	Infertility, female, of pituitary-hypothalamic origin	ICD9CM	90
		628.2	Infertility, female, of tubal origin	ICD9CM	581
		628.3	Infertility, female, of uterine origin	ICD9CM	177
		628.4	Infertility, female, of cervical or vaginal origin	ICD9CM	26
		628.8	Infertility, female, of other specified origin	ICD9CM	4527
		628.9	Infertility, female, of unspecified origin	ICD9CM	25568
		E28.2	Polycystic ovarian syndrome	ICD10CM	44081
		N91.0	Primary amenorrhea	ICD10CM	569
		N91.1	Secondary amenorrhea	ICD10CM	3587
		N91.2	Amenorrhea, unspecified	ICD10CM	17363
		N91.3	Primary oligomenorrhea	ICD10CM	212
		N91.4	Secondary oligomenorrhea	ICD10CM	1136

© 2025 Fourman LT et al. *JAMA Network Open*.

Organ	ICD concept	ICD code	ICD description	ICD year	Count
		N91.5	Oligomenorrhea, unspecified	ICD10CM	2932
		N97	Female infertility	ICD10CM	134
		N97.0	Female infertility associated with anovulation	ICD10CM	3750
		N97.1	Female infertility of tubal origin	ICD10CM	777
		N97.2	Female infertility of uterine origin	ICD10CM	216
		N97.8	Female infertility of other origin	ICD10CM	2584
		N97.9	Female infertility, unspecified	ICD10CM	40732
**Reproductive (male)**	Male hypogonadism	257.2	Other testicular hypofunction	ICD9CM	38308
		E29.1	Testicular hypofunction	ICD10CM	62360
**Musculoskeletal**	Osteoarthritis	M16	Osteoarthritis of hip	ICD10CM	164
		M16.0	Bilateral primary osteoarthritis of hip	ICD10CM	28637
		M16.10	Unilateral primary osteoarthritis, unspecified hip	ICD10CM	8670
		M16.11	Unilateral primary osteoarthritis, right hip	ICD10CM	55888
		M16.12	Unilateral primary osteoarthritis, left hip	ICD10CM	44452
		M16.9	Osteoarthritis of hip, unspecified	ICD10CM	16269
		M17	Osteoarthritis of knee	ICD10CM	405
		M17.0	Bilateral primary osteoarthritis of knee	ICD10CM	131888
		M17.10	Unilateral primary osteoarthritis, unspecified knee	ICD10CM	28493
		M17.11	Unilateral primary osteoarthritis, right knee	ICD10CM	142390
		M17.12	Unilateral primary osteoarthritis, left knee	ICD10CM	129839
		M17.9	Osteoarthritis of knee, unspecified	ICD10CM	37173
**Lymphatic**	Lymphedema	457.1	Other lymphedema	ICD9CM	16400
		I89.0	Lymphedema, not elsewhere classified	ICD10CM	59397

© 2025 Fourman LT et al. *JAMA Network Open*.

eTable 4. Other Curated Phenotypic Information

Phenotype	Data sources	ICD concepts	Surveys		Phenotype definition
			**Survey name**	**Questions**	
**Race and ethnicity**	Surveys		The basics		We used the AoU curated phenotypes where they transformed the collected answers into race variable and ethnicity variable, the approach taken is detailed on their website.<br><br>Which categories describe you? Select all that apply. Note, you may select more than one group.<br>• American Indian or Alaska Native (For example: Aztec, Blackfeet Tribe, Mayan, Navajo Nation, Native Village of Barrow (Utqiagvik) Inupiat Traditional Government, Nome Eskimo Community, etc.)<br>• Asian (For example: Asian Indian, Chinese, Filipino, Japanese, Korean, Vietnamese, etc.) [Original source question text: Asian – Provide details below.]<br>• Black, African American, or African (For example: African American, Ethiopian, Haitian, Jamaican, Nigerian, Somali, etc.)<br>• Hispanic, Latino, or Spanish (For example: Colombian, Cuban, Dominican, Mexican or Mexican American, Puerto Rican, Salvadoran, etc.)<br>• Middle Eastern or North African (For example: Algerian, Egyptian, Iranian, Lebanese, Moroccan, Syrian, etc.)

© 2025 Fourman LT et al. *JAMA Network Open*.

Phenotype	Data sources	ICD concepts	Surveys		Phenotype definition
			**Survey name**	**Questions**	
					• Native Hawaiian or other Pacific Islander (For example: Chamorro, Fijian, Marshallese, Native Hawaiian, Tongan, etc.) [Original source question text: Native Hawaiian or other Pacific Islander – Provide details below.]<br>• White (For example: English, European, French, German, Irish, Italian, Polish, etc.) [Original source question text: White – Provide details below.]<br>• None of these fully describe me<br>• Prefer not to answer<br><br>(Branching logic categories are not shown; full survey can be found on AoU website)
**Smoking status**	EHR and surveys	eTable 5	Lifestyle	Have you smoked at least 100 cigarettes in your entire life?<br>• Yes<br>• No<br><br>Do you now smoke cigarettes every day, some days, or not at all Not at all<br>• Some days<br>• Every day	A variable with 3 levels was created:<br>• Nonsmoker<br>• Ever smoker<br>• Current smoker<br><br>Following the following ruling:<br>An affirmative response to “Have you smoked at least 100 cigarettes in your entire life?” to define “Ever smoker”, and the branching logic question ‘Do you now smoke cigarettes every day, some days or not at all?” with the response ‘every day’ or ‘some days’ to designate ‘Current smoker’, participants answering ‘No’ the first question were included as ‘Never smokers’. Participants that were classified as never smokers using survey data,

© 2025 Fourman LT et al. *JAMA Network Open*.

Phenotype	Data sources	ICD concepts	Surveys		Phenotype definition
			Survey name	Questions	
					were queried for ICD smoking related diagnosis, the presence of at least 2 occurrences of the code before baseline was considered as ever smoker. (**eTable 5**). This approach has been used and validated before.(6)
**Social determinants of health**					
**Employment**	Surveys	-	The basics	What is your current employment status? Please select 1 or more of these categories.<br>• Employed for wages (part- time or full-time)<br>• Self-employed<br>• Out of work for 1 year or more<br>• Out of work for less than 1 year<br>• A homemaker<br>• A student<br>• Retired<br>• Unable to work (disabled)<br>• Prefer not to answer	The values were mapped into the following categories:<br>Employed<br>• Employed for wages (part- time or full-time)<br>• Self-employed<br>Unemployed/other<br>• Out of work for 1 year or more<br>• Out of work for less than 1 year<br>• A homemaker<br>• A student<br>• Unable to work (disabled)<br>Retired<br>• Retired<br>in cases where the participant provided multiple answers, employed status was prioritized.

© 2025 Fourman LT et al. *JAMA Network Open*.

Phenotype	Data sources	ICD concepts	Surveys		Phenotype definition
			Survey name	Questions	
**Education**	Surveys	-	The basics	What is the highest grade or year of school you completed?<br><br>• Never attended school or only attended kindergarten<br>• Grades 1 through 4 (Primary)<br>• Grades 5 through 8 (Middle school)<br>• Grades 9 through 11 (Some high school)<br>• Grade 12 or GED (High school graduate)<br>• 1 to 3 years after high school (Some college, associate's degree, or technical school)<br>• College 4 years or more (College graduate)<br>• Advanced degree (Master's, Doctorate, etc.)<br>• Prefer not to answer	A new variable with the following levels mapped was created:<br><br>Higher education<br>• College 4 years or more (College graduate)<br>• Advanced degree (Master's, Doctorate, etc.)<br><br>Some higher education<br>• 1 to 3 years after high school (Some college, associate's degree, or technical school)<br><br>Lower education – all the rest
**Insurance**	Surveys	-	The basics	Are you covered by health insurance or some other kind of health care plan?	The values were mapped into a new variable with the following levels:<br><br>• Yes

© 2025 Fourman LT et al. *JAMA Network Open*.

Phenotype	Data sources	ICD concepts	Surveys		Phenotype definition
			Survey name	Questions	
				• Yes<br>• No<br>• Prefer not to say	• No<br>• Don't know
**Annual income**	Surveys	-	The basics	What is your annual household income from all sources?<br><br>• Less than $10,000<br>• $10,000- $24,999<br>• $25,000-$34,999<br>• $35,000-$49,999<br>• $50,000- $74,999<br>• $75,000-$99,999<br>• $100,000-$149,999<br>• $150,000-$199,999<br>• $200,000 or more<br>• Prefer not to answer	The values were mapped into a new variable with the following levels:<br><br>• Low (<35K)<br>• Middle-Low (35K-75K)<br>• Middle-High (75K-150K)<br>• High (>150K)

© 2025 Fourman LT et al. *JAMA Network Open*.

eTable 5. Smoking-Related *ICD* Codes

ICD code	ICD description	ICD year	Count
**305.1**	Tobacco uses disorder	ICD9CM	188488
**649**	Tobacco use disorder complicating pregnancy, childbirth, or the puerperium, unspecified as to episode of care or not applicable	ICD9CM	207
**649.01**	Tobacco use disorder complicating pregnancy, childbirth, or the puerperium, delivered, with or without mention of antepartum condition	ICD9CM	387
**649.03**	Tobacco use disorder complicating pregnancy, childbirth, or the puerperium, antepartum condition or complication	ICD9CM	1452
**649.04**	Tobacco use disorder complicating pregnancy, childbirth, or the puerperium, postpartum condition or complication	ICD9CM	28
**989.84**	Toxic effect of tobacco	ICD9CM	126
**F17.2**	Nicotine dependence	ICD10CM	83
**F17.20**	Nicotine dependence, unspecified	ICD10CM	9
**F17.200**	Nicotine dependence, unspecified, uncomplicated	ICD10CM	182590
**F17.208**	Nicotine dependence, unspecified, with other nicotine-induced disorders	ICD10CM	198
**F17.209**	Nicotine dependence, unspecified, with unspecified nicotine-induced disorders	ICD10CM	1396
**F17.21**	Nicotine dependence, cigarettes	ICD10CM	11
**F17.210**	Nicotine dependence, cigarettes, uncomplicated	ICD10CM	208280
**F17.218**	Nicotine dependence, cigarettes, with other nicotine-induced disorders	ICD10CM	2438
**F17.219**	Nicotine dependence, cigarettes, with unspecified nicotine-induced disorders	ICD10CM	3678
**F17.220**	Nicotine dependence, chewing tobacco, uncomplicated	ICD10CM	1748
**F17.228**	Nicotine dependence, chewing tobacco, with other nicotine-induced disorders	ICD10CM	62
**F17.229**	Nicotine dependence, chewing tobacco, with unspecified nicotine-induced disorders	ICD10CM	160
**F17.29**	Nicotine dependence, other tobacco product	ICD10CM	1
**F17.290**	Nicotine dependence, other tobacco product, uncomplicated	ICD10CM	8473
**F17.298**	Nicotine dependence, other tobacco product, with other nicotine-induced disorders	ICD10CM	82
**F17.299**	Nicotine dependence, other tobacco product, with unspecified nicotine-induced disorders	ICD10CM	204
**O99.330**	Smoking (tobacco) complicating pregnancy, unspecified trimester	ICD10CM	668

© 2025 Fourman LT et al. *JAMA Network Open*.

ICD code	ICD description	ICD year	Count
**O99.331**	Smoking (tobacco) complicating pregnancy, first trimester	ICD10CM	1022
**O99.332**	Smoking (tobacco) complicating pregnancy, second trimester	ICD10CM	951
**O99.333**	Smoking (tobacco) complicating pregnancy, third trimester	ICD10CM	467
**T65.221A**	Toxic effect of tobacco cigarettes, accidental (unintentional), initial encounter	ICD10CM	29
**T65.221D**	Toxic effect of tobacco cigarettes, accidental (unintentional), subsequent encounter	ICD10CM	18
**T65.222A**	Toxic effect of tobacco cigarettes, intentional self-harm, initial encounter	ICD10CM	13
**T65.222D**	Toxic effect of tobacco cigarettes, intentional self-harm, subsequent encounter	ICD10CM	4
**T65.222S**	Toxic effect of tobacco cigarettes, intentional self-harm, sequela	ICD10CM	4
**T65.224A**	Toxic effect of tobacco cigarettes, undetermined, initial encounter	ICD10CM	2
**T65.224D**	Toxic effect of tobacco cigarettes, undetermined, subsequent encounter	ICD10CM	1
**T65.224S**	Toxic effect of tobacco cigarettes, undetermined, sequela	ICD10CM	4
**T65.291A**	Toxic effect of other tobacco and nicotine, accidental (unintentional), initial encounter	ICD10CM	20
**T65.292A**	Toxic effect of other tobacco and nicotine, intentional self-harm, initial encounter	ICD10CM	8
**T65.294A**	Toxic effect of other tobacco and nicotine, undetermined, initial encounter	ICD10CM	4

© 2025 Fourman LT et al. *JAMA Network Open*.

eTable 6. Cardiovascular-Related *ICD* Codes

ICD concept	ICD code	ICD description	ICD year	Occurrences
**Acute heart failure**	428.21	Acute systolic heart failure	ICD9CM	1784
	428.31	Acute diastolic heart failure	ICD9CM	1076
	428.41	Acute combined systolic and diastolic heart failure	ICD9CM	655
	I50.31	Acute diastolic (congestive) heart failure	ICD10CM	16313
	I50.811	Acute right heart failure	ICD10CM	340
**Acute myocardial infarction**	410	Acute myocardial infarction	ICD9CM	18
	410	Acute myocardial infarction, of anterolateral wall	ICD9CM	27
	410	Acute myocardial infarction of anterolateral wall, episode of care unspecified	ICD9CM	361
	410.01	Acute myocardial infarction of anterolateral wall, initial episode of care	ICD9CM	141
	410.02	Acute myocardial infarction of anterolateral wall, subsequent episode of care	ICD9CM	22
	410.1	Acute myocardial infarction, of other anterior wall	ICD9CM	10
	410.1	Acute myocardial infarction of other anterior wall, episode of care unspecified	ICD9CM	1189
	410.11	Acute myocardial infarction of other anterior wall, initial episode of care	ICD9CM	385
	410.12	Acute myocardial infarction of other anterior wall, subsequent episode of care	ICD9CM	702
	410.2	Acute myocardial infarction of inferolateral wall, episode of care unspecified	ICD9CM	60
	410.21	Acute myocardial infarction of inferolateral wall, initial episode of care	ICD9CM	155
	410.22	Acute myocardial infarction of inferolateral wall, subsequent episode of care	ICD9CM	34
	410.3	Acute myocardial infarction, of inferoposterior wall	ICD9CM	2
	410.3	Acute myocardial infarction of inferoposterior wall, episode of care unspecified	ICD9CM	75
	410.31	Acute myocardial infarction of inferoposterior wall, initial episode of care	ICD9CM	336
	410.32	Acute myocardial infarction of inferoposterior wall, subsequent episode of care	ICD9CM	51
	410.4	Acute myocardial infarction, of other inferior wall	ICD9CM	4

© 2025 Fourman LT et al. *JAMA Network Open*.

ICD concept	ICD code	ICD description	ICD year	Occurrences
	410.4	Acute myocardial infarction of other inferior wall, episode of care unspecified	ICD9CM	596
	410.41	Acute myocardial infarction of other inferior wall, initial episode of care	ICD9CM	835
	410.42	Acute myocardial infarction of other inferior wall, subsequent episode of care	ICD9CM	160
	410.5	Acute myocardial infarction, of other lateral wall	ICD9CM	1
	410.5	Acute myocardial infarction of other lateral wall, episode of care unspecified	ICD9CM	49
	410.51	Acute myocardial infarction of other lateral wall, initial episode of care	ICD9CM	93
	410.52	Acute myocardial infarction of other lateral wall, subsequent episode of care	ICD9CM	37
	410.6	True posterior wall infarction, episode of care unspecified	ICD9CM	56
	410.61	True posterior wall infarction, initial episode of care	ICD9CM	27
	410.62	True posterior wall infarction, subsequent episode of care	ICD9CM	7
	410.7	Subendocardial infarction, episode of care unspecified	ICD9CM	6322
	410.71	Subendocardial infarction, initial episode of care	ICD9CM	3246
	410.72	Subendocardial infarction, subsequent episode of care	ICD9CM	520
	410.8	Acute myocardial infarction, of other specified sites	ICD9CM	95
	410.8	Acute myocardial infarction of other specified sites, episode of care unspecified	ICD9CM	206
	410.81	Acute myocardial infarction of other specified sites, initial episode of care	ICD9CM	137
	410.82	Acute myocardial infarction of other specified sites, subsequent episode of care	ICD9CM	131
	410.9	Acute myocardial infarction, unspecified site	ICD9CM	153
	410.9	Acute myocardial infarction of unspecified site, episode of care unspecified	ICD9CM	23811
	410.91	Acute myocardial infarction of unspecified site, initial episode of care	ICD9CM	1264
	410.92	Acute myocardial infarction of unspecified site, subsequent episode of care	ICD9CM	597
	I21	Acute myocardial infarction	ICD10CM	139
	I21.0	ST elevation (STEMI) myocardial infarction of anterior wall	ICD10CM	1

© 2025 Fourman LT et al. *JAMA Network Open*.

ICD concept	ICD code	ICD description	ICD year	Occurrences
	I21.01	ST elevation (STEMI) myocardial infarction involving left main coronary artery	ICD10CM	187
	I21.02	ST elevation (STEMI) myocardial infarction involving left anterior descending coronary artery	ICD10CM	2914
	I21.09	ST elevation (STEMI) myocardial infarction involving other coronary artery of anterior wall	ICD10CM	1844
	I21.11	ST elevation (STEMI) myocardial infarction involving right coronary artery	ICD10CM	2023
	I21.19	ST elevation (STEMI) myocardial infarction involving other coronary artery of inferior wall	ICD10CM	2325
	I21.21	ST elevation (STEMI) myocardial infarction involving left circumflex coronary artery	ICD10CM	638
	I21.29	ST elevation (STEMI) myocardial infarction involving other sites	ICD10CM	1211
	I21.3	ST elevation (STEMI) myocardial infarction of unspecified site	ICD10CM	8574
	I21.4	Non-ST elevation (NSTEMI) myocardial infarction	ICD10CM	41157
	I21.9	Acute myocardial infarction, unspecified	ICD10CM	10006
**Stroke**	434.11	Cerebral embolism with cerebral infarction	ICD9CM	3935
	434.91	Cerebral artery occlusion, unspecified with cerebral infarction	ICD9CM	49282
	I63	Cerebral infarction	ICD10CM	2
	I63.00	Cerebral infarction due to thrombosis of unspecified precerebral artery	ICD10CM	792
	I63.011	Cerebral infarction due to thrombosis of right vertebral artery	ICD10CM	84
	I63.012	Cerebral infarction due to thrombosis of left vertebral artery	ICD10CM	165
	I63.013	Cerebral infarction due to thrombosis of bilateral vertebral arteries	ICD10CM	270
	I63.019	Cerebral infarction due to thrombosis of unspecified vertebral artery	ICD10CM	264
	I63.02	Cerebral infarction due to thrombosis of basilar artery	ICD10CM	319
	I63.031	Cerebral infarction due to thrombosis of right carotid artery	ICD10CM	262
	I63.032	Cerebral infarction due to thrombosis of left carotid artery	ICD10CM	192
	I63.033	Cerebral infarction due to thrombosis of bilateral carotid arteries	ICD10CM	20
	I63.039	Cerebral infarction due to thrombosis of unspecified carotid artery	ICD10CM	199
	I63.09	Cerebral infarction due to thrombosis of other precerebral artery	ICD10CM	92

© 2025 Fourman LT et al. *JAMA Network Open*.

ICD concept	ICD code	ICD description	ICD year	Occurrences
	I63.10	Cerebral infarction due to embolism of unspecified precerebral artery	ICD10CM	1079
	I63.111	Cerebral infarction due to embolism of right vertebral artery	ICD10CM	305
	I63.112	Cerebral infarction due to embolism of left vertebral artery	ICD10CM	88
	I63.113	Cerebral infarction due to embolism of bilateral vertebral arteries	ICD10CM	4
	I63.119	Cerebral infarction due to embolism of unspecified vertebral artery	ICD10CM	227
	I63.12	Cerebral infarction due to embolism of basilar artery	ICD10CM	215
	I63.131	Cerebral infarction due to embolism of right carotid artery	ICD10CM	408
	I63.132	Cerebral infarction due to embolism of left carotid artery	ICD10CM	159
	I63.133	Cerebral infarction due to embolism of bilateral carotid arteries	ICD10CM	97
	I63.139	Cerebral infarction due to embolism of unspecified carotid artery	ICD10CM	84
	I63.19	Cerebral infarction due to embolism of other precerebral artery	ICD10CM	85
	I63.20	Cerebral infarction due to unspecified occlusion or stenosis of unspecified precerebral arteries	ICD10CM	325
	I63.211	Cerebral infarction due to unspecified occlusion or stenosis of right vertebral artery	ICD10CM	128
	I63.212	Cerebral infarction due to unspecified occlusion or stenosis of left vertebral artery	ICD10CM	263
	I63.213	Cerebral infarction due to unspecified occlusion or stenosis of bilateral vertebral arteries	ICD10CM	43
	I63.219	Cerebral infarction due to unspecified occlusion or stenosis of unspecified vertebral artery	ICD10CM	107
	I63.22	Cerebral infarction due to unspecified occlusion or stenosis of basilar artery	ICD10CM	385
	I63.231	Cerebral infarction due to unspecified occlusion or stenosis of right carotid arteries	ICD10CM	812
	I63.232	Cerebral infarction due to unspecified occlusion or stenosis of left carotid arteries	ICD10CM	1078
	I63.233	Cerebral infarction due to unspecified occlusion or stenosis of bilateral carotid arteries	ICD10CM	216
	I63.239	Cerebral infarction due to unspecified occlusion or stenosis of unspecified carotid artery	ICD10CM	927
	I63.29	Cerebral infarction due to unspecified occlusion or stenosis of other precerebral arteries	ICD10CM	146

© 2025 Fourman LT et al. *JAMA Network Open*.

ICD concept	ICD code	ICD description	ICD year	Occurrences
	I63.30	Cerebral infarction due to thrombosis of unspecified cerebral artery	ICD10CM	1748
	I63.311	Cerebral infarction due to thrombosis of right middle cerebral artery	ICD10CM	760
	I63.312	Cerebral infarction due to thrombosis of left middle cerebral artery	ICD10CM	808
	I63.313	Cerebral infarction due to thrombosis of bilateral middle cerebral arteries	ICD10CM	25
	I63.319	Cerebral infarction due to thrombosis of unspecified middle cerebral artery	ICD10CM	135
	I63.321	Cerebral infarction due to thrombosis of right anterior cerebral artery	ICD10CM	42
	I63.322	Cerebral infarction due to thrombosis of left anterior cerebral artery	ICD10CM	75
	I63.323	Cerebral infarction due to thrombosis of bilateral anterior cerebral arteries	ICD10CM	13
	I63.329	Cerebral infarction due to thrombosis of unspecified anterior cerebral artery	ICD10CM	32
	I63.331	Cerebral infarction due to thrombosis of right posterior cerebral artery	ICD10CM	50
	I63.332	Cerebral infarction due to thrombosis of left posterior cerebral artery	ICD10CM	42
	I63.333	Cerebral infarction due to thrombosis of bilateral posterior cerebral arteries	ICD10CM	6
	I63.339	Cerebral infarction due to thrombosis of unspecified posterior cerebral artery	ICD10CM	21
	I63.341	Cerebral infarction due to thrombosis of right cerebellar artery	ICD10CM	35
	I63.342	Cerebral infarction due to thrombosis of left cerebellar artery	ICD10CM	31
	I63.343	Cerebral infarction due to thrombosis of bilateral cerebellar arteries	ICD10CM	2
	I63.349	Cerebral infarction due to thrombosis of unspecified cerebellar artery	ICD10CM	98
	I63.39	Cerebral infarction due to thrombosis of other cerebral artery	ICD10CM	78
	I63.40	Cerebral infarction due to embolism of unspecified cerebral artery	ICD10CM	2778
	I63.411	Cerebral infarction due to embolism of right middle cerebral artery	ICD10CM	2137
	I63.412	Cerebral infarction due to embolism of left middle cerebral artery	ICD10CM	2560
	I63.413	Cerebral infarction due to embolism of bilateral middle cerebral arteries	ICD10CM	58
	I63.419	Cerebral infarction due to embolism of unspecified middle cerebral artery	ICD10CM	248
	I63.421	Cerebral infarction due to embolism of right anterior cerebral artery	ICD10CM	165

© 2025 Fourman LT et al. *JAMA Network Open*.

ICD concept	ICD code	ICD description	ICD year	Occurrences
	I63.422	Cerebral infarction due to embolism of left anterior cerebral artery	ICD10CM	461
	I63.423	Cerebral infarction due to embolism of bilateral anterior cerebral arteries	ICD10CM	22
	I63.429	Cerebral infarction due to embolism of unspecified anterior cerebral artery	ICD10CM	151
	I63.431	Cerebral infarction due to embolism of right posterior cerebral artery	ICD10CM	372
	I63.432	Cerebral infarction due to embolism of left posterior cerebral artery	ICD10CM	402
	I63.433	Cerebral infarction due to embolism of bilateral posterior cerebral arteries	ICD10CM	27
	I63.439	Cerebral infarction due to embolism of unspecified posterior cerebral artery	ICD10CM	309
	I63.441	Cerebral infarction due to embolism of right cerebellar artery	ICD10CM	183
	I63.442	Cerebral infarction due to embolism of left cerebellar artery	ICD10CM	282
	I63.443	Cerebral infarction due to embolism of bilateral cerebellar arteries	ICD10CM	164
	I63.449	Cerebral infarction due to embolism of unspecified cerebellar artery	ICD10CM	139
	I63.49	Cerebral infarction due to embolism of other cerebral artery	ICD10CM	396
	I63.50	Cerebral infarction due to unspecified occlusion or stenosis of unspecified cerebral artery	ICD10CM	8636
	I63.511	Cerebral infarction due to unspecified occlusion or stenosis of right middle cerebral artery	ICD10CM	2366
	I63.512	Cerebral infarction due to unspecified occlusion or stenosis of left middle cerebral artery	ICD10CM	2822
	I63.513	Cerebral infarction due to unspecified occlusion or stenosis of bilateral middle cerebral arteries	ICD10CM	18
	I63.519	Cerebral infarction due to unspecified occlusion or stenosis of unspecified middle cerebral artery	ICD10CM	157
	I63.521	Cerebral infarction due to unspecified occlusion or stenosis of right anterior cerebral artery	ICD10CM	226
	I63.522	Cerebral infarction due to unspecified occlusion or stenosis of left anterior cerebral artery	ICD10CM	395
	I63.523	Cerebral infarction due to unspecified occlusion or stenosis of bilateral anterior cerebral arteries	ICD10CM	49
	I63.529	Cerebral infarction due to unspecified occlusion or stenosis of unspecified anterior cerebral artery	ICD10CM	46

© 2025 Fourman LT et al. *JAMA Network Open*.

ICD concept	ICD code	ICD description	ICD year	Occurrences
	I63.531	Cerebral infarction due to unspecified occlusion or stenosis of right posterior cerebral artery	ICD10CM	353
	I63.532	Cerebral infarction due to unspecified occlusion or stenosis of left posterior cerebral artery	ICD10CM	482
	I63.533	Cerebral infarction due to unspecified occlusion or stenosis of bilateral posterior cerebral arteries	ICD10CM	28
	I63.539	Cerebral infarction due to unspecified occlusion or stenosis of unspecified posterior cerebral artery	ICD10CM	56
	I63.541	Cerebral infarction due to unspecified occlusion or stenosis of right cerebellar artery	ICD10CM	161
	I63.542	Cerebral infarction due to unspecified occlusion or stenosis of left cerebellar artery	ICD10CM	179
	I63.543	Cerebral infarction due to unspecified occlusion or stenosis of bilateral cerebellar arteries	ICD10CM	9
	I63.549	Cerebral infarction due to unspecified occlusion or stenosis of unspecified cerebellar artery	ICD10CM	55
	I63.59	Cerebral infarction due to unspecified occlusion or stenosis of other cerebral artery	ICD10CM	401
	I63.6	Cerebral infarction due to cerebral venous thrombosis, nonpyogenic	ICD10CM	123
	I63.8	Other cerebral infarction	ICD10CM	1174
	I63.81	Other cerebral infarction due to occlusion or stenosis of small artery	ICD10CM	7738
	I63.89	Other cerebral infarction	ICD10CM	3016
	I63.9	Cerebral infarction, unspecified	ICD10CM	97822
	I69.30	Unspecified sequelae of cerebral infarction	ICD10CM	5035

© 2025 Fourman LT et al. *JAMA Network Open*.

eTable 7. BMI-Based Eligibility Criteria for Obesity Pharmacotherapy

<table>
<tr><th>BMI-based criteria</th><th>Obesity-Related Comorbidity</th><th>Definition</th></tr>
<tr><td>≥ 30 kg/m^2</td><td>-</td><td>-</td></tr>
<tr><td rowspan="4">27 kg/m^2 ≤ BMI < 30 kg/m^2

AND

At least 1 obesity-related comorbidity</td><td>Hypertension</td><td>Cardiovascular (arterial) (“hypertension”) within 1 year preceding the baseline visit as defined in eTable 2</td></tr>
<tr><td>Obstructive Sleep Apnea</td><td>Upper airways (“obstructive sleep apnea”) within 1 year preceding the baseline visit as defined in eTable 2</td></tr>
<tr><td>Dyslipidemia</td><td>Either of the following within 1 year preceding the baseline visit:
• Dyslipidemia as defined by ICD code in eTable 3
• Abnormal labs: Triglycerides ≥ 150 mg/dl OR HDL < 40 mg/dL</td></tr>
<tr><td>Cardiovascular disease</td><td>Any of the cardiovascular associated conditions defined in eTable 2:
• Cardiovascular (ventricular) (“heart failure”) (any prior history)
• Cardiovascular (“heart failure”) (any prior history)
• Cardiovascular (atrial) (“atrial fibrillation”) (within 1 year preceding baseline)
• Cardiovascular (pulmonary) (“pulmonary hypertension”) (within 1 year preceding baseline)
• Cardiovascular (thrombosis) (“thrombosis”) (within 1 year preceding baseline)

OR

Any prior history of myocardial infarction or stroke defined in eTable 6.</td></tr>
</table>

© 2025 Fourman LT et al. *JAMA Network Open*.

eTable 8. Social Determinants of Health by Obesity Status and Phenotype Per the New Definition

Characteristic	Overall, n (%)	No Obesity, n (%)	Obesity, n (%)	*P*-value[c]	Anthropometric Only, n (%)	BMI + Anthropometric, n (%)	*P*-value[d]
	(N=301026)	(N=94665)	(N=206361)		(N=78047)	(N=128314)	
**Education**							
**Lower Education**	85323 (28.3)	22146 (23.4)	63177 (30.6)	<0.001	22275 (28.5)	40902 (31.9)	<0.001
**Some Higher Education**	77759 (25.8)	20203 (21.3)	57556 (27.9)		18518 (23.7)	39038 (30.4)	
**Higher Education**	130610 (43.4)	50285 (53.1)	80325 (38.9)		35124 (45.0)	45201 (35.2)	
**Employment**							
**Employed**	146447 (48.6)	52782 (55.8)	93665 (45.4)	<0.001	32694 (41.9)	60971 (47.5)	<0.001
**Unemployed/Other**	80667 (26.8)	24154 (25.5)	56513 (27.4)		19157 (24.5)	37356 (29.1)	
**Retired**	65078 (21.6)	14980 (15.8)	50098 (24.3)		24013 (30.8)	26085 (20.3)	
**Insurance**							
**No**	18443 (6.1)	5865 (6.2)	12578 (6.1)	0.225	4463 (5.7)	8115 (6.3)	<0.001
**Yes**	274687 (91.3)	86167 (91.0)	188520 (91.4)		71608 (91.7)	116912 (91.1)	
**Income**							
**Low (<35K)**	98514 (32.7)	27781 (29.3)	70733 (34.3)	<0.001	23599 (30.2)	47134 (36.7)	<0.001
**Middle-Low (35K-75K)**	56401 (18.7)	16766 (17.7)	39635 (19.2)		13688 (17.5)	25947 (20.2)	
**Middle-High (75K-150K)**	56146 (18.7)	18955 (20.0)	37191 (18.0)		15364 (19.7)	21827 (17.0)	
**High (>150K)**	33727 (11.2)	14656 (15.5)	19071 (9.2)		9753 (12.5)	9318 (7.3)	

[a,b] Obesity and no obesity were defined based on the *Lancet* Commission framework. Obesity was further subdivided into BMI-plus-anthropometric and anthropometric-only phenotypes.
[c] *P*-value for No Obesity vs. Obesity groups. [d] *P*-value for Anthropometric Only vs. BMI + Anthropometric groups.

© 2025 Fourman LT et al. *JAMA Network Open*.

eTable 9. Organ Dysfunction by Obesity Status and Phenotype Per the New Definition

Organ Dysfunction	No Obesity, n (%)	Obesity, n (%)	P-value[c]	Anthropometric Only, n (%)	BMI + Anthropometric, n (%)	P-value[d]
	(N=94665)	(N=206361)		(N=78047)	(N=128314)	
**Central Nervous System**	50 (0.05)	410 (0.2)	<0.001	43 (0.06)	367 (0.29)	<0.001
**Obstructive Sleep Apnea**	2142 (2.26)	24820 (12.03)	<0.001	5352 (6.86)	19468 (15.17)	<0.001
**Hypoventilation**	3 (0)	294 (0.14)	<0.001	7 (0.01)	287 (0.22)	<0.001
**Heart Failure**	999 (1.06)	7213 (3.5)	<0.001	2298 (2.94)	4915 (3.83)	<0.001
**Atrial Fibrillation**	1698 (1.79)	8093 (3.92)	<0.001	3133 (4.01)	4960 (3.87)	0.09
**Pulmonary Hypertension**	326 (0.34)	1888 (0.91)	<0.001	576 (0.74)	1312 (1.02)	<0.001
**Thrombosis**	618 (0.65)	3170 (1.54)	<0.001	1000 (1.28)	2170 (1.69)	<0.001
**Hypertension**	11663 (12.32)	70191 (34.01)	<0.001	23719 (30.39)	46472 (36.22)	<0.001
**Metabolic**	1934 (2.04)	23492 (11.38)	<0.001	7087 (9.08)	16405 (12.79)	<0.001
**Liver**	587 (0.62)	3133 (1.52)	<0.001	1152 (1.48)	1981 (1.54)	0.23
**Renal**	1672 (1.77)	10199 (4.94)	<0.001	3905 (5)	6294 (4.91)	0.32
**Urinary**	1018 (1.08)	4906 (2.38)	<0.001	1486 (1.9)	3420 (2.67)	<0.001
**Reproductive (female)**	1305 (2.18)	3802 (3.07)	<0.001	682 (1.71)	3120 (3.72)	<0.001
**Reproductive (male)**	208 (0.6)	1436 (1.74)	<0.001	518 (1.36)	918 (2.06)	<0.001
**Musculoskeletal**	4428 (4.68)	19921 (9.65)	<0.001	6195 (7.94)	13726 (10.7)	<0.001
**Lymphedema**	28 (0.03)	309 (0.15)	<0.001	38 (0.05)	271 (0.21)	<0.001
**Physical Limitation**	6170 (6.52)	31296 (15.17)	<0.001	8866 (11.36)	22430 (17.48)	<0.001
**Any Dysfunction**	24085 (25.44)	108650 (52.65)	<0.001	37193 (47.65)	71457 (55.69)	<0.001

[a,b] Obesity and no obesity were defined based on the *Lancet* Commission framework. Obesity was further subdivided into BMI-plus-anthropometric and anthropometric-only phenotypes.
[c] *P*-value for No Obesity vs. Obesity groups. [d] *P*-value for Anthropometric Only vs. BMI + Anthropometric groups.

© 2025 Fourman LT et al. *JAMA Network Open*.

eFigure 3. Distribution of New Obesity Phenotypes by Age

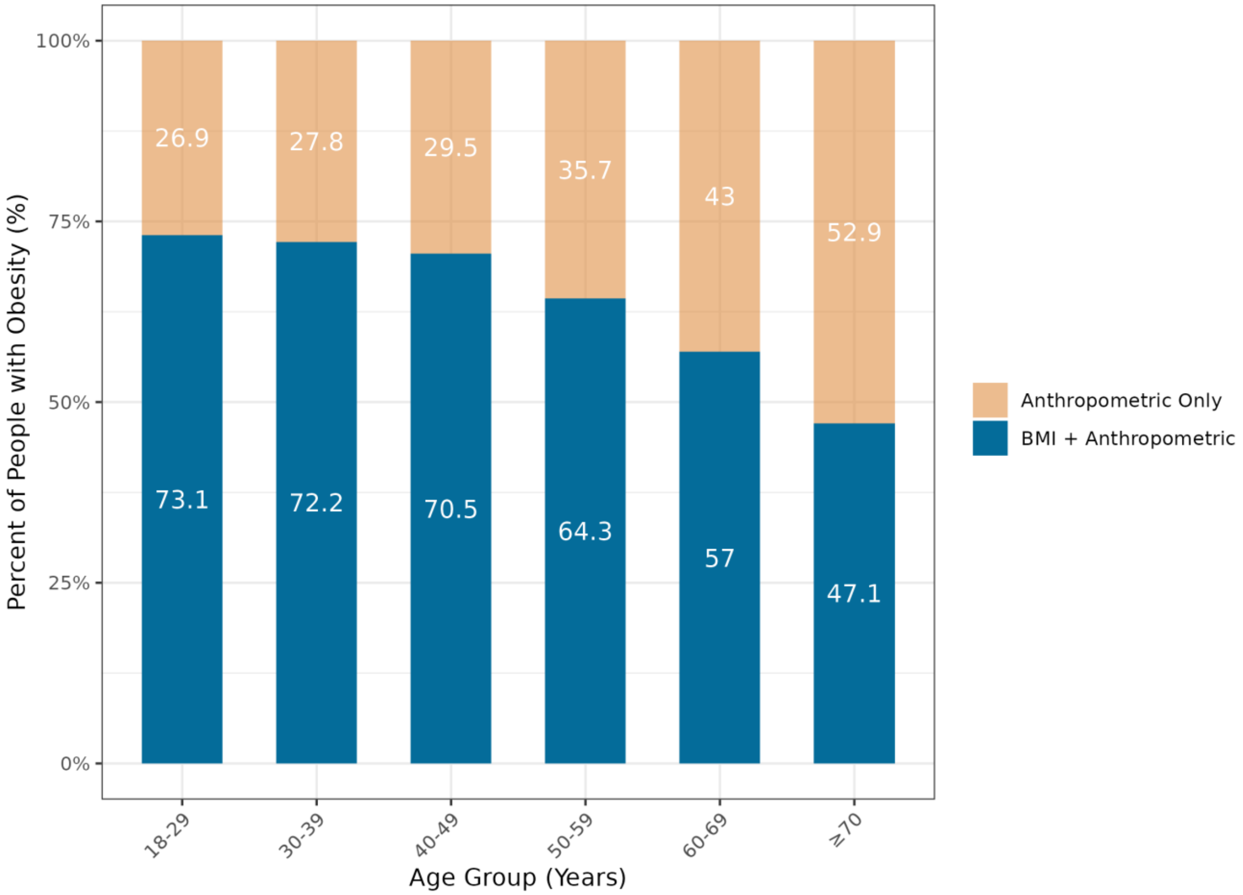

The proportion of anthropometric-only obesity among those with obesity by the new *Lancet* Commission definition increased with age within the All of Us cohort (P_{trend}<0.001). Among the subset of participants 70 years and over, more than half of cases of obesity were ascribed to anthropometric-only obesity.

© 2025 Fourman LT et al. *JAMA Network Open*.

eFigure 4. Distribution of BMI Among Individuals With Anthropometric-Only Obesity

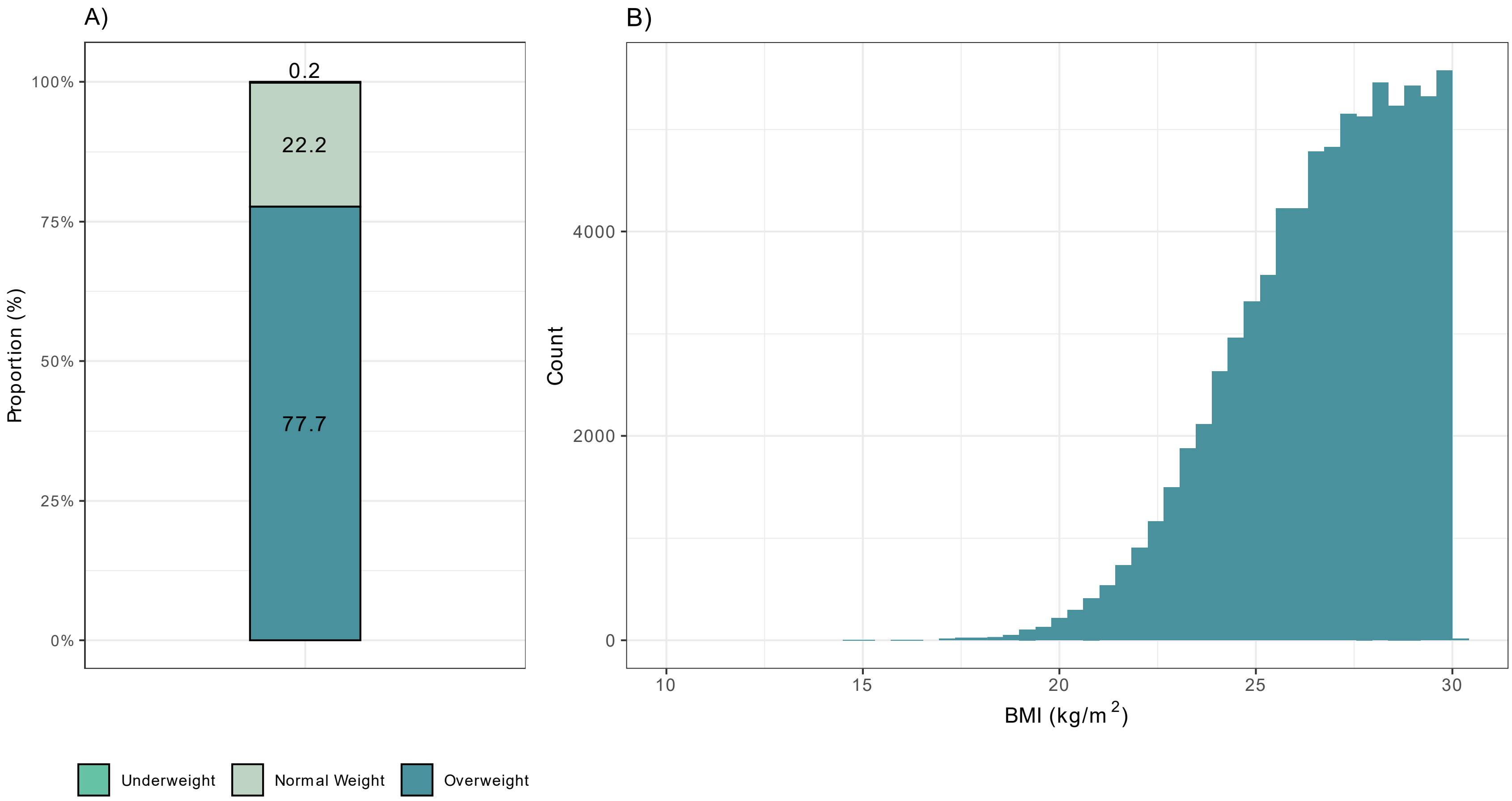

A) Among individuals with anthropometric-only obesity by the new *Lancet* Commission definition, 22.3% had a BMI that was defined as normal or underweight using race-specific cutoffs per the traditional obesity classification. **B)** The distribution of BMI among individuals with anthropometric-only obesity is shown.

© 2025 Fourman LT et al. *JAMA Network Open*.

eFigure 5. Elevated Anthropometrics by New Obesity Phenotype

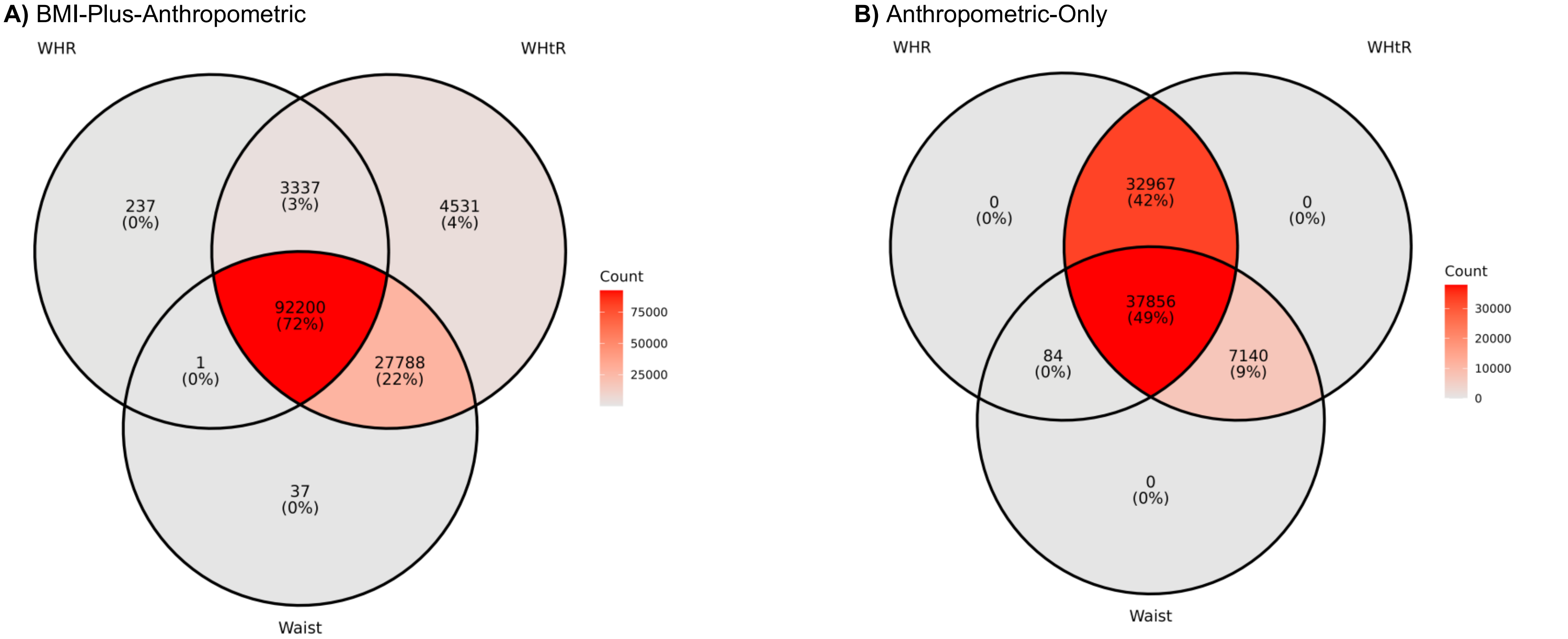

The distribution of elevated waist circumference, waist-hip ratio, and waist-height ratio as defined by sex- and race-specific cutoffs is shown for **A)** BMI-plus-anthropometric and **B)** anthropometric-only obesity. Based on the new *Lancet* Commission framework, BMI-plus-anthropometric obesity was defined as BMI above the traditional obesity threshold with at least one elevated anthropometric measure or BMI >40 kg/m^2, whereas anthropometric-only obesity was defined as at least two elevated anthropometric measures with BMI below the traditional obesity threshold. *Abbreviations:* WHR, waist-hip ratio; WHtR, waist-height ratio.

© 2025 Fourman LT et al. *JAMA Network Open*.

eFigure 6. Prevalence of Clinical Obesity by the Traditional and New Definitions Among the All of Us Cohort

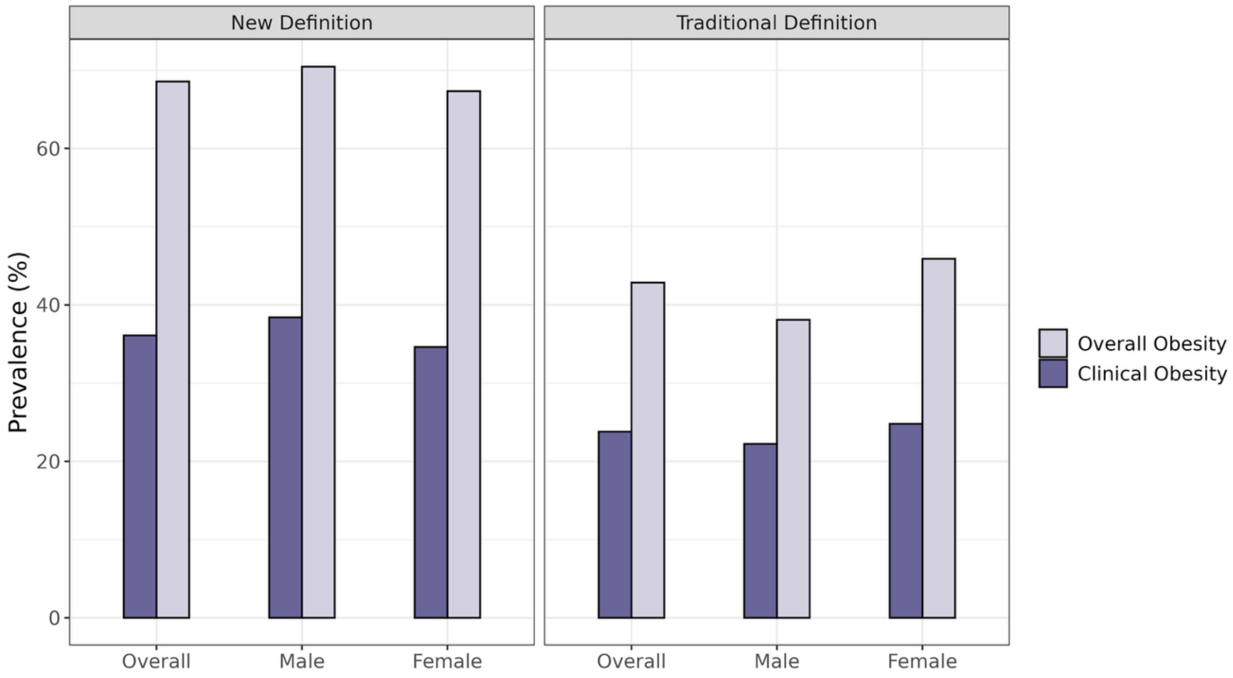

Approximately half of individuals in the All of Us cohort with obesity by the traditional and new definitions had manifestations of organ dysfunction and/or physical limitation consistent with clinical obesity. With transition to the new definition, the absolute prevalence of clinical obesity increased in parallel with a rise in overall obesity prevalence.

© 2025 Fourman LT et al. *JAMA Network Open*.

eFigure 7. Proportion of Individuals With Obesity Per the New Definition Meeting Clinical Obesity Criteria

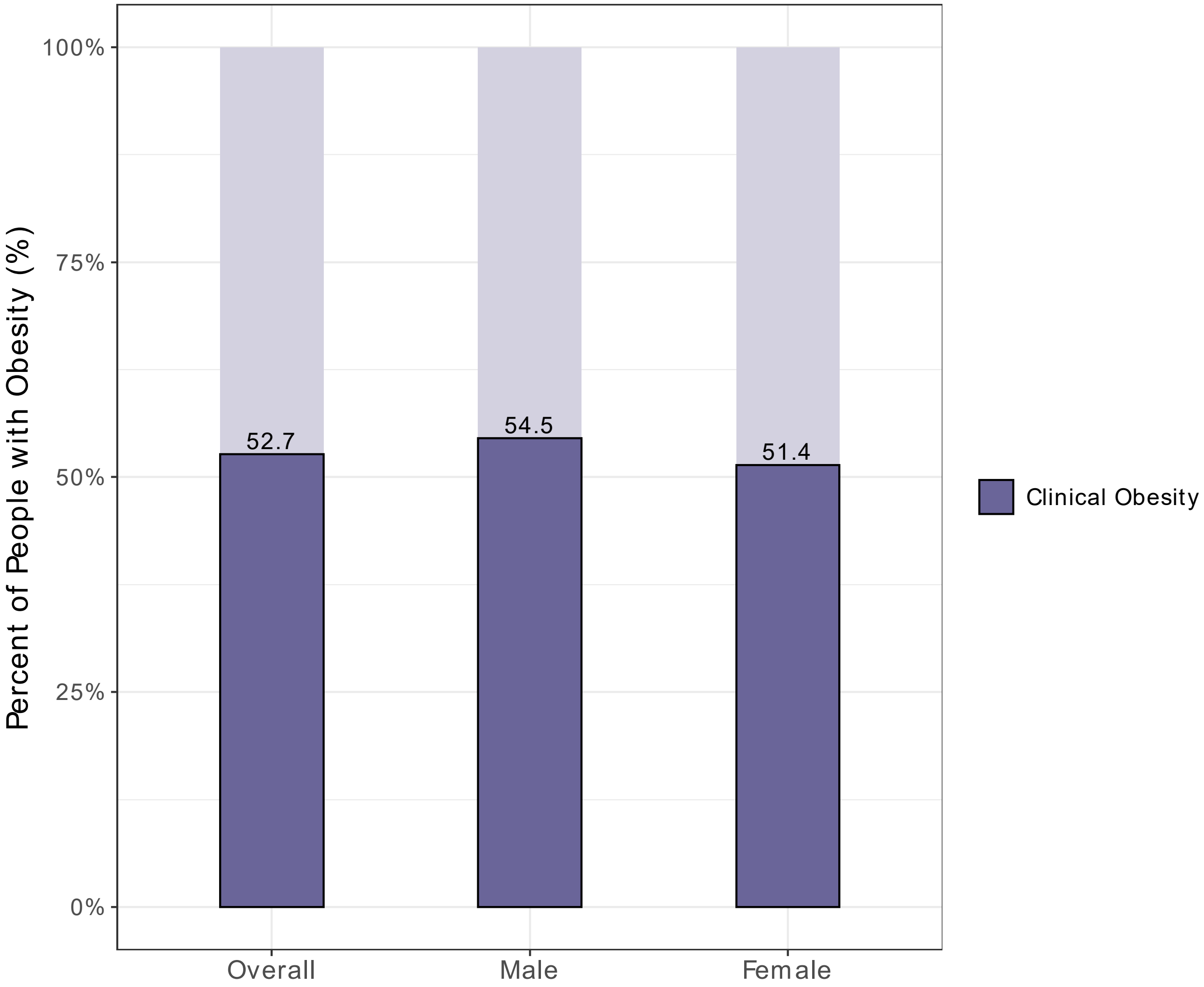

The proportion of individuals with obesity per the new *Lancet* Commission definition who have organ dysfunction and/or physical limitation consistent with clinical obesity is shown overall and by sex.

© 2025 Fourman LT et al. *JAMA Network Open*.

eFigure 8. Prevalence of Obesity and Clinical Obesity by the Traditional and New Definitions Across Age Groups

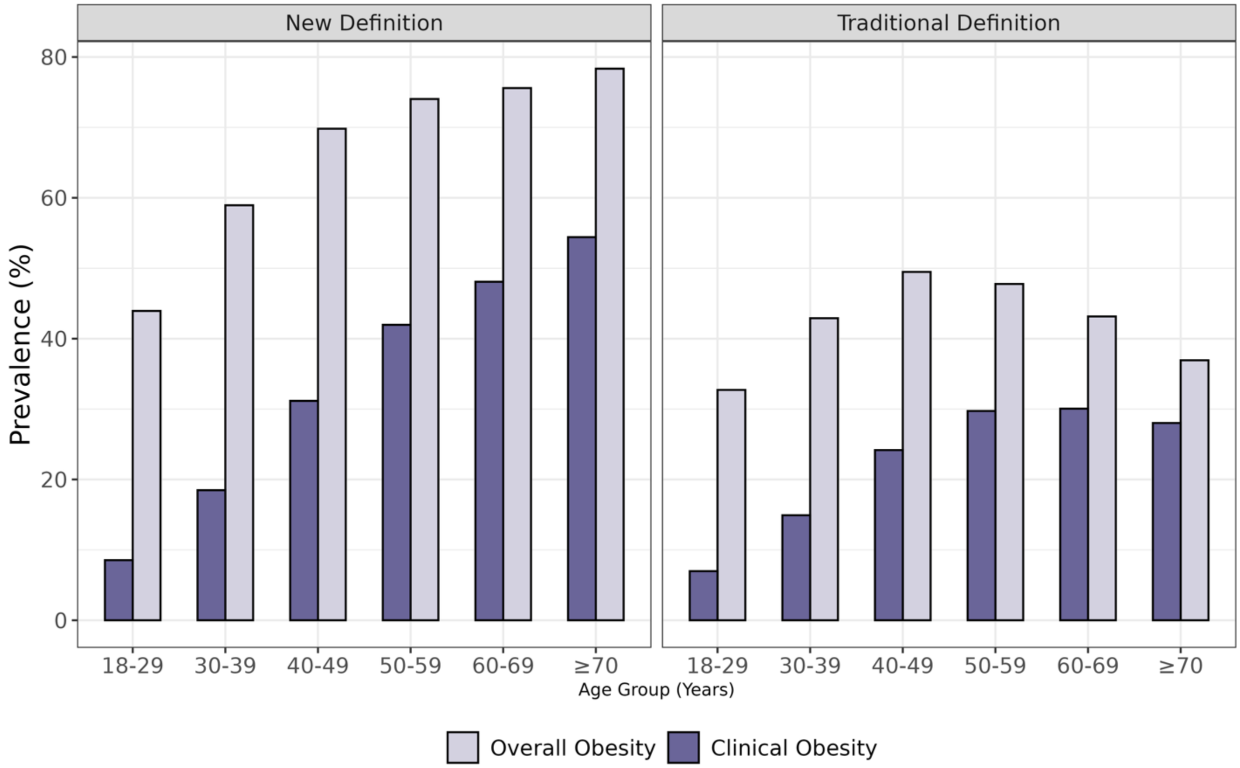

Under the new definition, prevalence of obesity and clinical obesity increased with age to comprise 78.3% and 54.4% of all individuals ≥70 years, respectively (P_{trend}<0.001 each). The transition from the traditional to the new definitions led to a rise in frequency of both obesity and clinical obesity across all age strata.

© 2025 Fourman LT et al. *JAMA Network Open*.

eFigure 9. Prevalence of Obesity and Clinical Obesity Per the New Definition by Race Among the All of Us Cohort

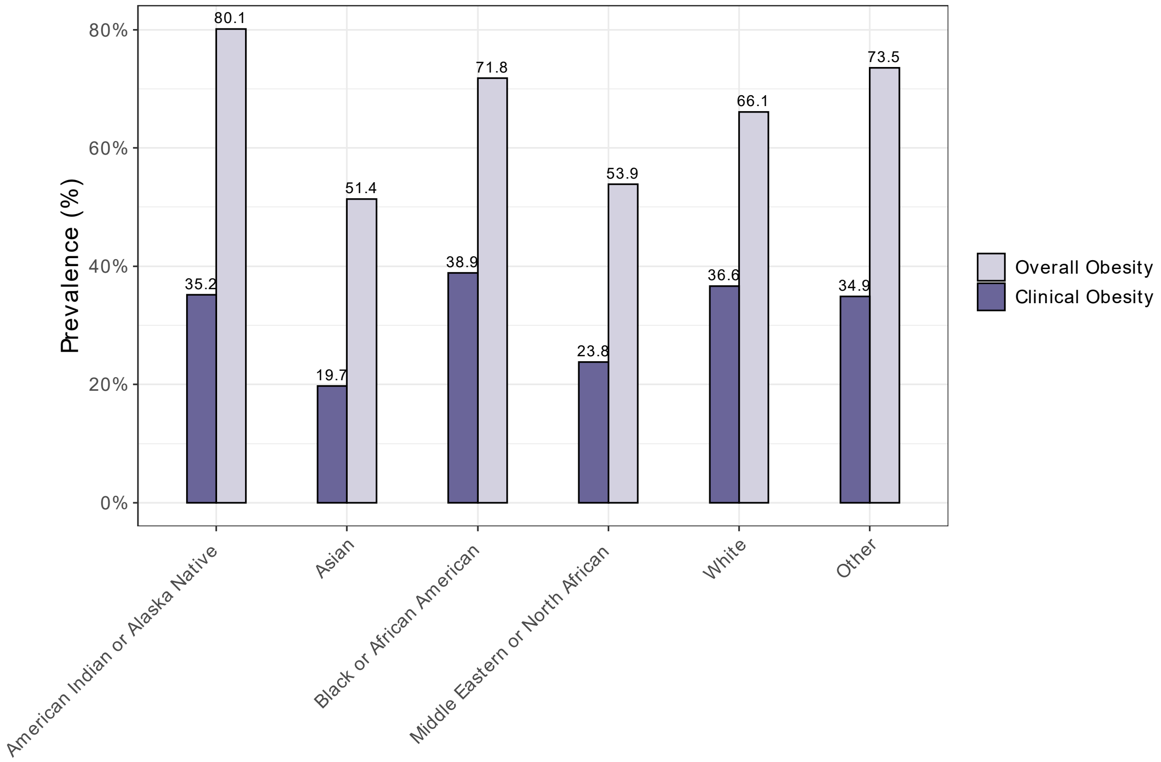

Prevalence of obesity and clinical obesity by the new *Lancet* Commission definition differed between racial groups (*P*<0.001). The lowest prevalence of obesity and clinical obesity was among Asians.

© 2025 Fourman LT et al. *JAMA Network Open*.

eFigure 10. Prevalence of Obesity by the New Definition Stratified by Traditional BMI Category

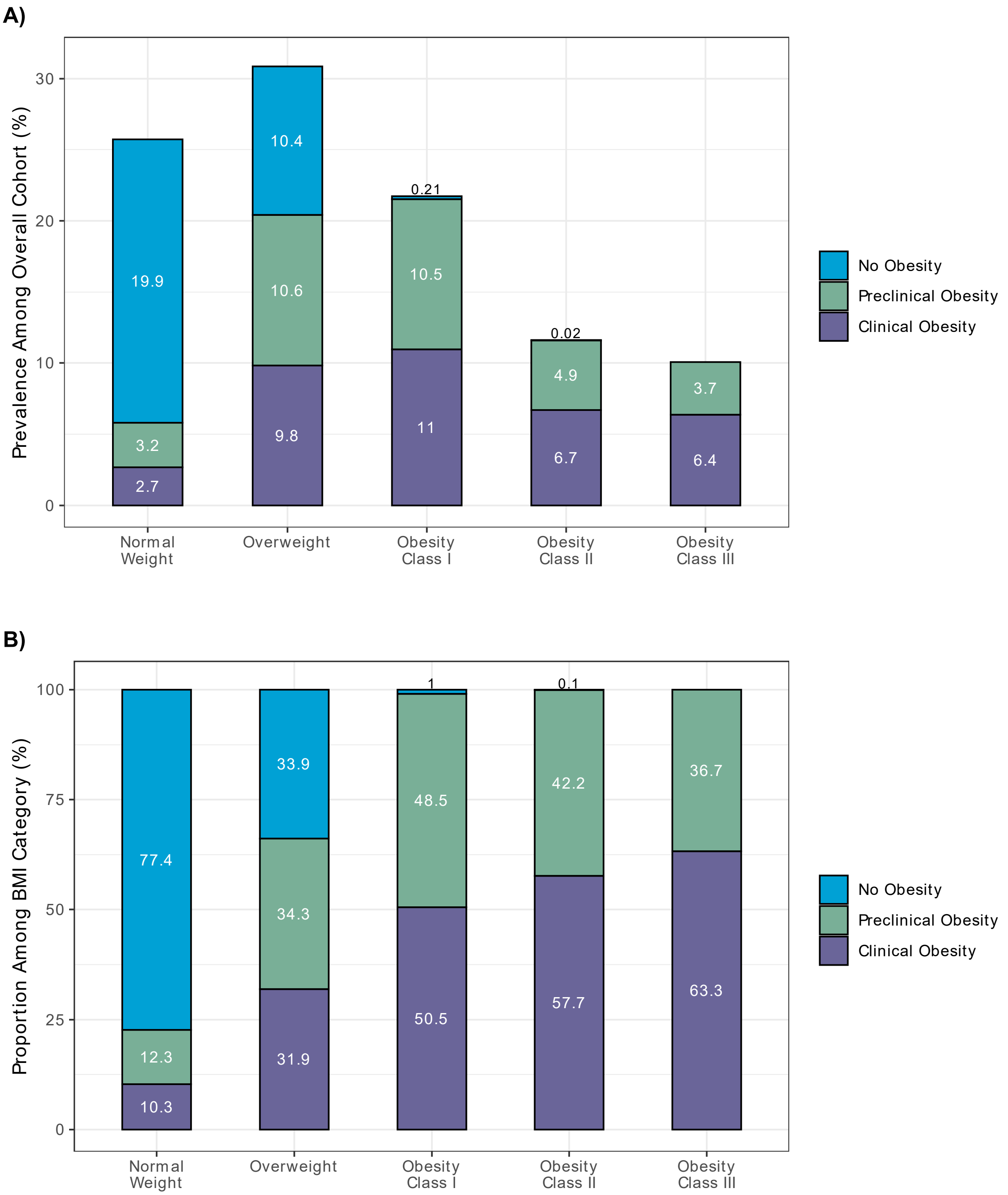

Prevalence of clinical obesity, preclinical obesity, and no obesity per the new *Lancet* Commission definition is shown **A)** among the overall cohort, stratified by BMI category, and **B)** as a proportion within each BMI category. BMI category was defined using race-specific BMI cutoffs per the traditional obesity classification.

© 2025 Fourman LT et al. *JAMA Network Open*.

eFigure 11. Burden of Organ Dysfunction by New Obesity Phenotype

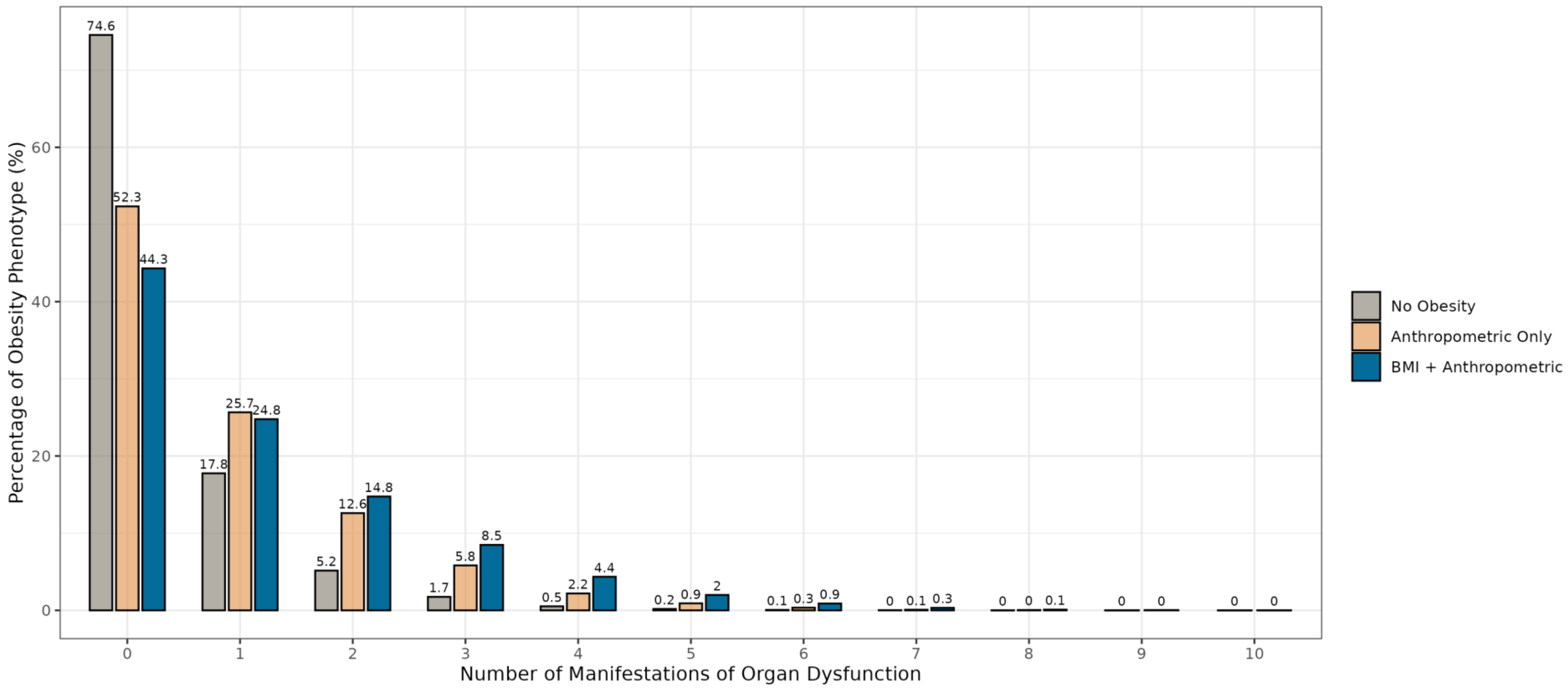

Individuals with BMI-plus-anthropometric obesity had a higher number of manifestations of organ dysfunction, as evidenced by a rightward shift in distribution, compared to individuals with anthropometric-only obesity (*P*<0.001).

© 2025 Fourman LT et al. *JAMA Network Open*.

eFigure 12. Association of Organ Dysfunction With BMI in Individuals With Obesity Per the New Definition

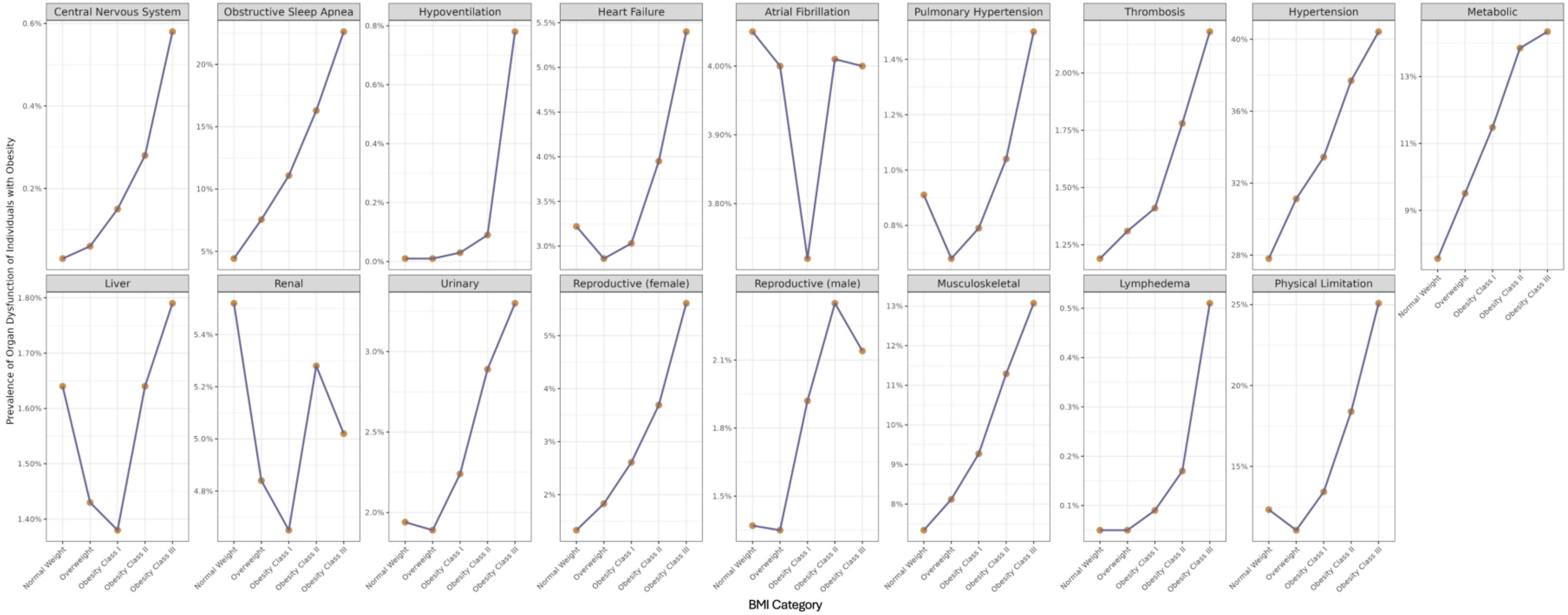

Among individuals with obesity by the new *Lancet* Commission definition, the prevalence of organ dysfunction generally rose with higher BMI category. BMI category was defined using race-specific cutoffs per the traditional obesity classification.

© 2025 Fourman LT et al. *JAMA Network Open*.

eFigure 13. Differential Characteristics of New Obesity Phenotypes by Sex

A)

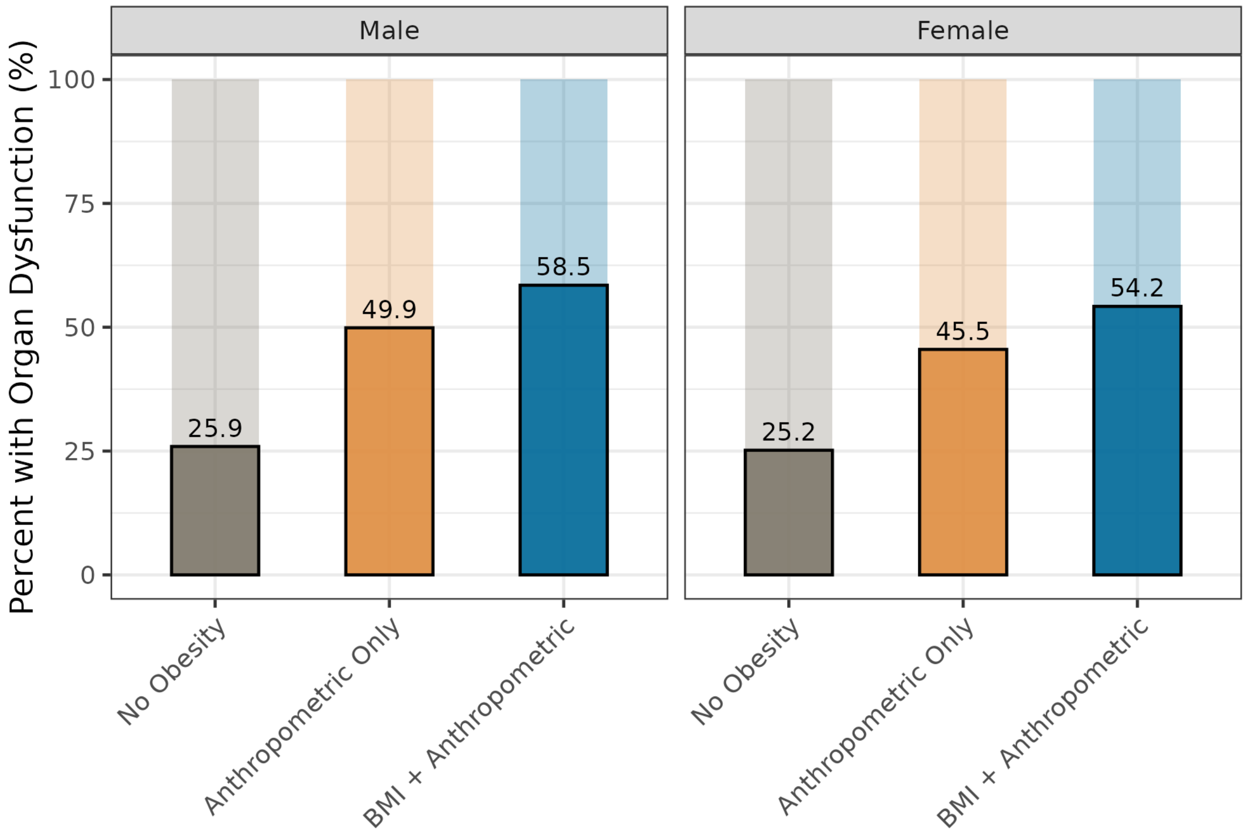

B)

Sex	Exposure	Odds Ratio (95% CI)
Male	No Obesity	Ref.
	Anthropometric Only	1.59 (1.53-1.64)
	BMI + Anthropometric	3.09 (2.99-3.2)
Female	No Obesity	Ref.
	Anthropometric Only	1.82 (1.77-1.87)
	BMI + Anthropometric	3.29 (3.21-3.38)

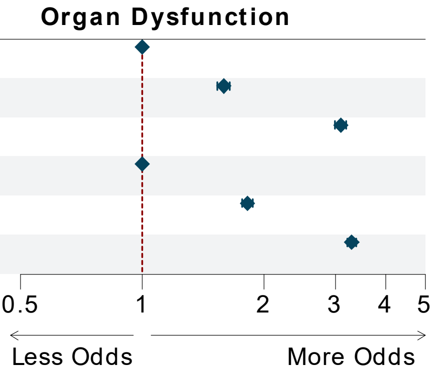

© 2025 Fourman LT et al. *JAMA Network Open*.

eFigure 13 (cont.). Differential Characteristics of New Obesity Phenotypes by Sex

C)

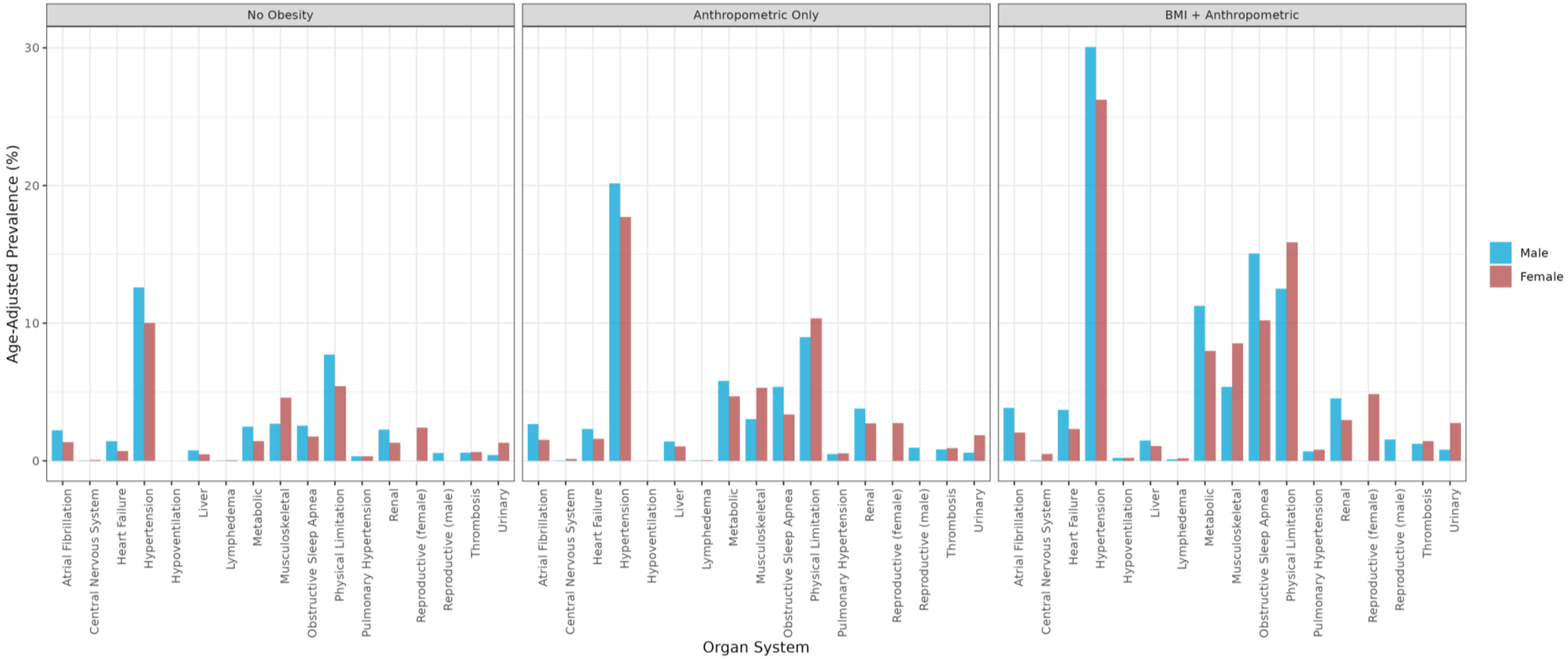

© 2025 Fourman LT et al. *JAMA Network Open*.

eFigure 13 (cont.). Differential Characteristics of New Obesity Phenotypes by Sex

D)

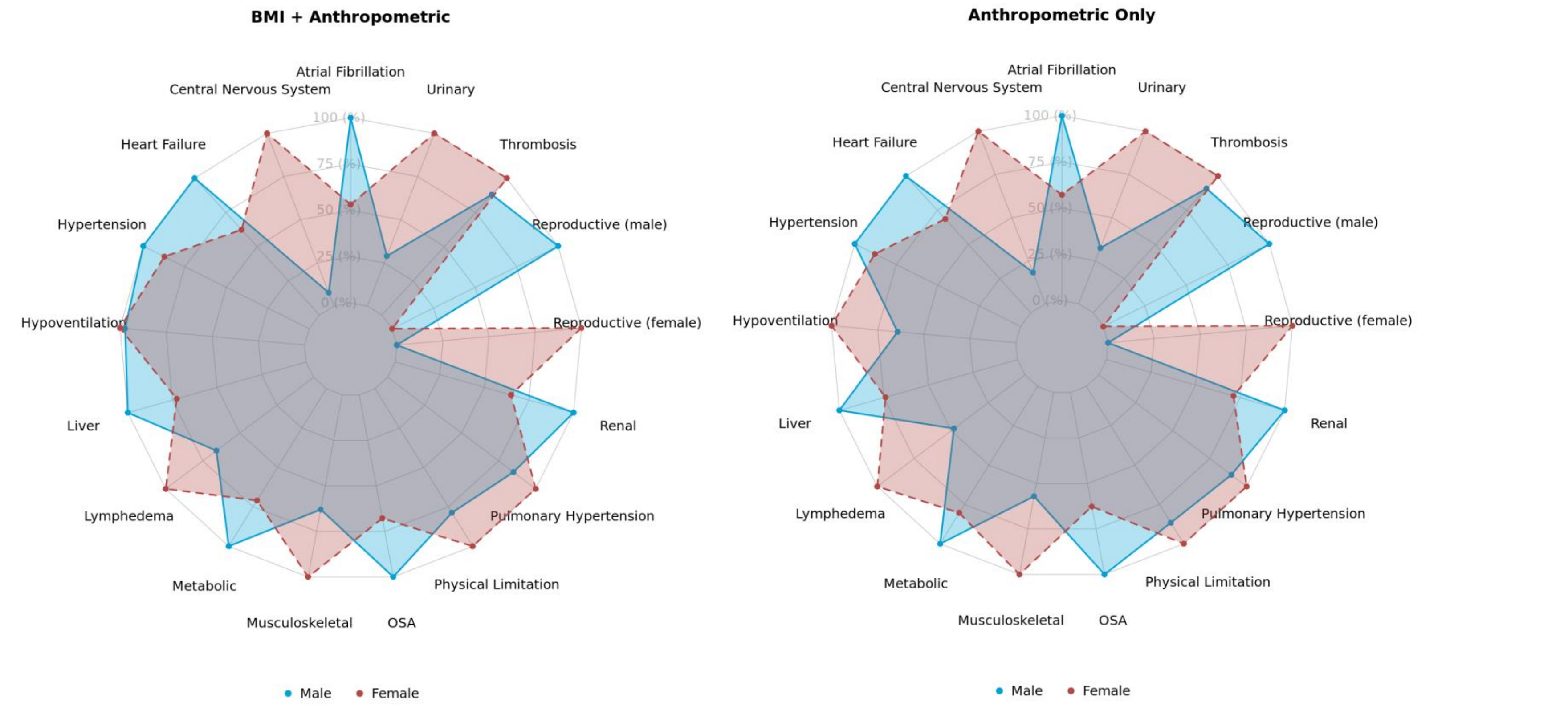

A) Proportions of individuals with organ dysfunction are shown for those with BMI-plus-anthropometric obesity, anthropometric-only obesity, and no obesity per the new *Lancet* Commission definition, stratified by sex. Within each sex, organ dysfunction tended to be higher among individuals with BMI-plus anthropometric versus anthropometric-only obesity. **B)** BMI-plus-anthropometric obesity and anthropometric-only obesity were consistently associated with elevated odds of organ dysfunction compared to no obesity across sexes. Forest plot displays odds of organ dysfunction with 95% confidence intervals in a model adjusted for age and race among individuals with BMI-plus-anthropometric and anthropometric-only obesity compared to no obesity stratified by sex. **C)** The age-adjusted prevalences of individual manifestations of organ dysfunction were compared between sexes among each obesity phenotype. **D)** Spider plots show relative age-adjusted prevalences of manifestations of organ dysfunction among individuals with BMI-plus-anthropometric and anthropometric-only obesity by sex with each axis representing a specific manifestation. The higher prevalence of each manifestation among the two groups is standardized to 100%, with the prevalence for the other sex expressed relative to this value. Non-overlapping regions highlight unique profiles of organ dysfunction by sex, suggesting differences in clinical presentation.

© 2025 Fourman LT et al. *JAMA Network Open*.

eFigure 14. Differential Characteristics of New Obesity Phenotypes Across Racial Groups

A)

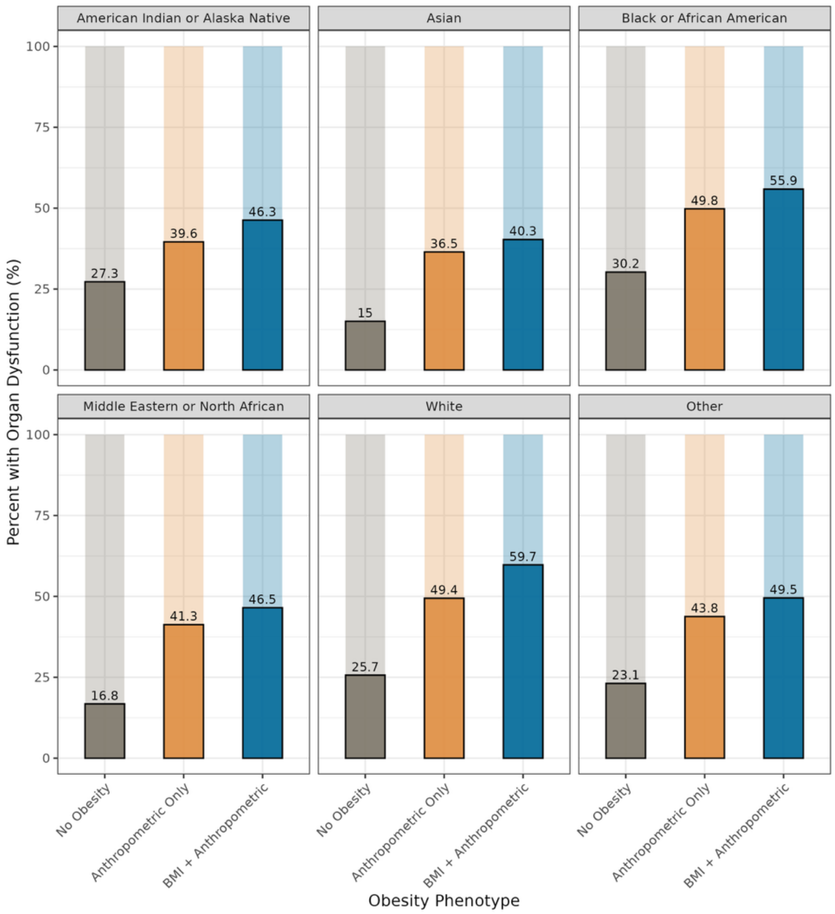

B)

Race	Exposure	Odds Ratio (95% CI)
American Indian or Alaska Native	No Obesity	Ref.
	Anthropometric Only	1.24 (1.02-1.52)
	BMI + Anthropometric	2.11 (1.76-2.54)
Asians	No Obesity	Ref.
	Anthropometric Only	1.86 (1.64-2.12)
	BMI + Anthropometric	3.15 (2.78-3.57)
Black or African American	No Obesity	Ref.
	Anthropometric Only	1.54 (1.46-1.62)
	BMI + Anthropometric	2.46 (2.35-2.57)
Middle Eastern or North African	No Obesity	Ref.
	Anthropometric Only	1.96 (1.46-2.64)
	BMI + Anthropometric	3.15 (2.41-4.13)
White	No Obesity	Ref.
	Anthropometric Only	1.84 (1.78-1.89)
	BMI + Anthropometric	3.84 (3.74-3.95)
Other	No Obesity	Ref.
	Anthropometric Only	1.58 (1.5-1.66)
	BMI + Anthropometric	2.69 (2.58-2.82)

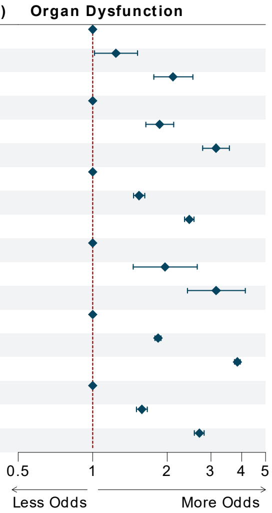

© 2025 Fourman LT et al. *JAMA Network Open*.

eFigure 14 (cont.). Differential Characteristics of New Obesity Phenotypes Across Racial Groups

C)

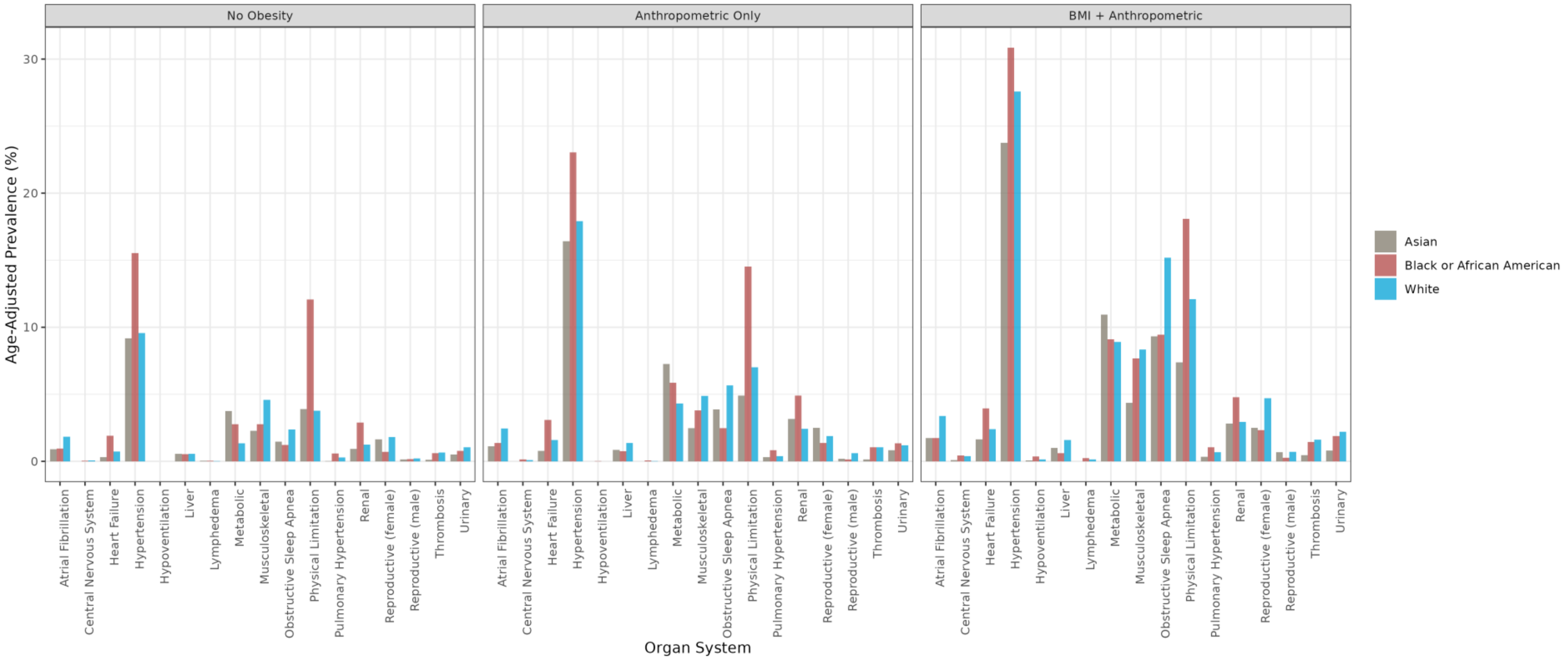

© 2025 Fourman LT et al. *JAMA Network Open*.

eFigure 14 (cont.). Differential Characteristics of New Obesity Phenotypes Across Racial Groups

D)

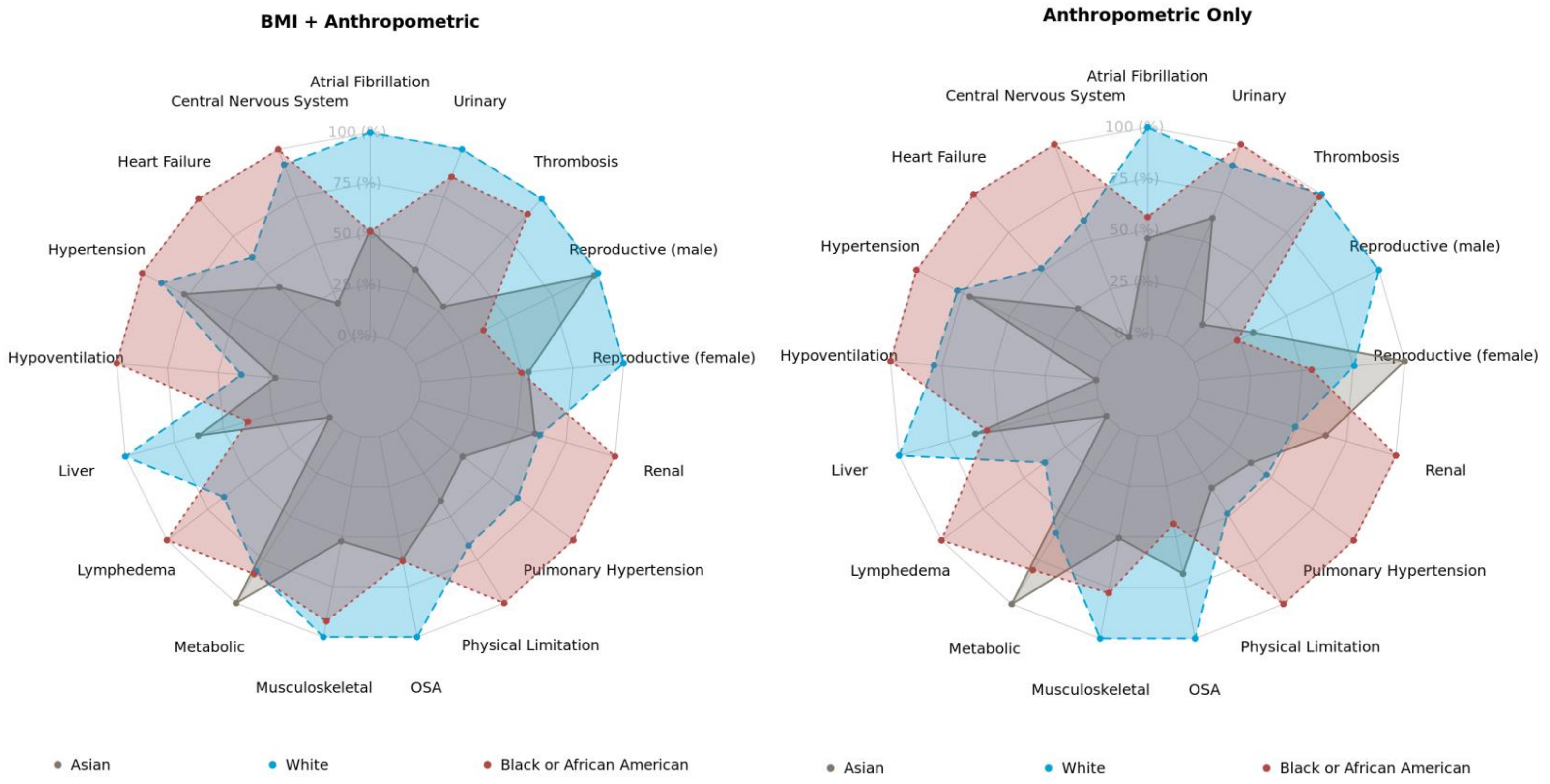

A) Proportions of individuals with organ dysfunction are shown for those with BMI-plus-anthropometric obesity, anthropometric-only obesity, and no obesity per the new *Lancet* Commission definition, stratified by race. Within each racial group, organ dysfunction tended to be higher among individuals with BMI-plus anthropometric versus anthropometric-only obesity. Asians had the lowest rates of organ dysfunction among those both with and without obesity (*P*<0.001 each). **B)** BMI-plus-anthropometric obesity and anthropometric-only obesity were consistently associated with elevated odds of organ dysfunction compared to no obesity across racial groups. Forest plot displays odds of organ dysfunction with 95% confidence intervals in a model adjusted for age and sex among individuals with BMI-plus-anthropometric and anthropometric-only obesity compared to no obesity stratified by race. **C)** The age-adjusted prevalences of individual manifestations of organ dysfunction were compared across racial groups among each obesity phenotype. Asians tended to have a lower prevalence of most manifestations of organ dysfunction, except for a higher prevalence of metabolic dysfunction (*P*<0.001). **D)** Spider plots show relative age-adjusted prevalences of manifestations of organ dysfunction among individuals with BMI-plus-anthropometric and anthropometric-only obesity by race with each axis representing a specific manifestation. The highest prevalence of each manifestation among groups is standardized to 100%, with prevalences for other races expressed relative to this value. Non-overlapping regions highlight unique profiles of organ dysfunction among racial groups, suggesting differences in clinical presentation.

© 2025 Fourman LT et al. *JAMA Network Open*.

eFigure 15. Patterns of Organ Dysfunction Among Individuals With Clinical Obesity Per the New Definition

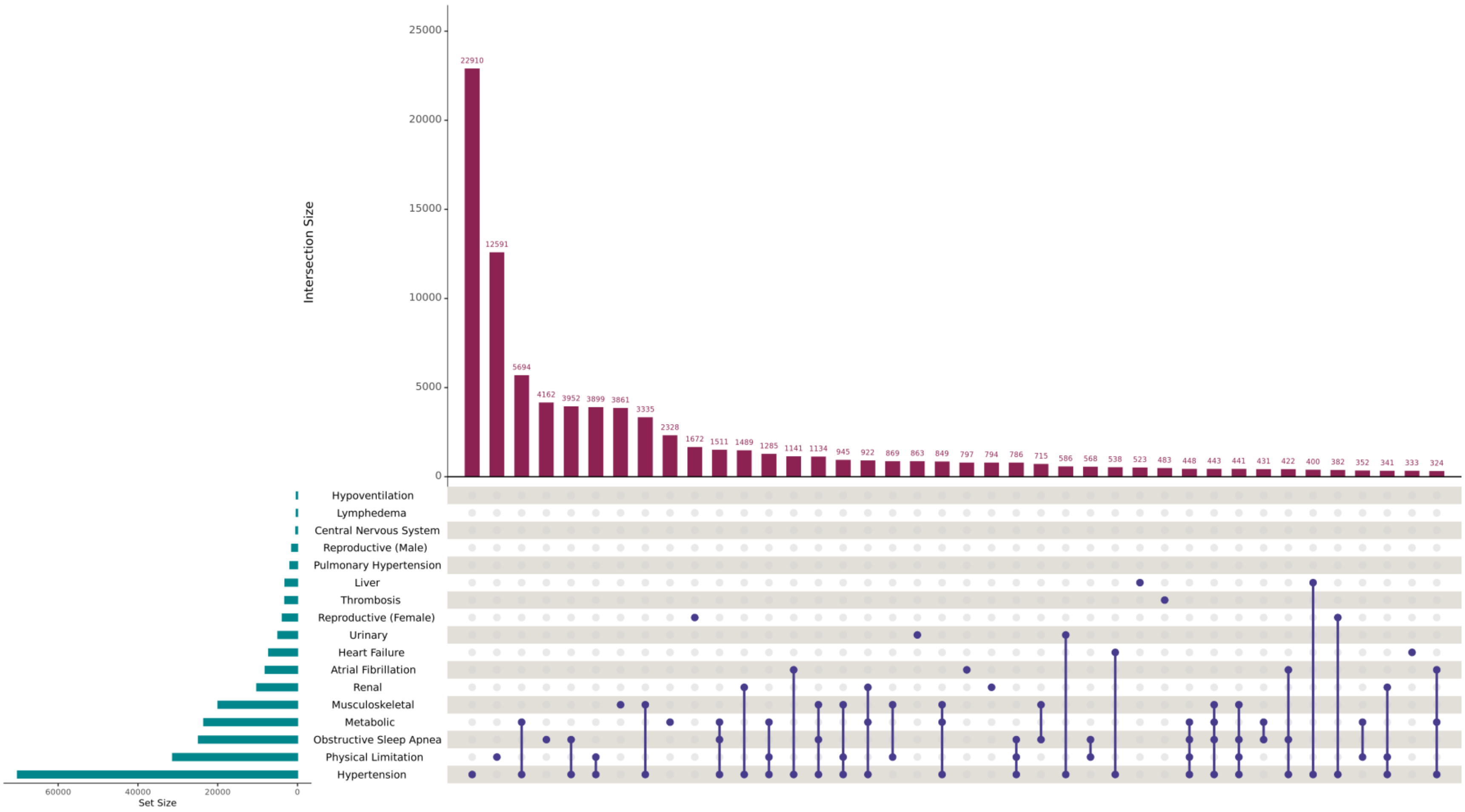

The UpSet plot shows the 40 most frequent patterns of organ dysfunction among individuals in the All of Us cohort with clinical obesity per the new *Lancet* Commission definition. The horizontal axis represents unique combinations of organ dysfunction as shown by dots and connecting lines. The vertical axis displays the number of participants that exhibit each combination of organ dysfunction. The leftward axis displays the number of individuals with each individual manifestation. The top 3 most common patterns of organ dysfunction among individuals with clinical obesity were hypertension alone, followed by physical limitation alone, followed by hypertension plus metabolic dysfunction.

© 2025 Fourman LT et al. *JAMA Network Open*.

eFigure 16. Obesity Pharmacotherapy Eligibility by Classification Scheme

A)

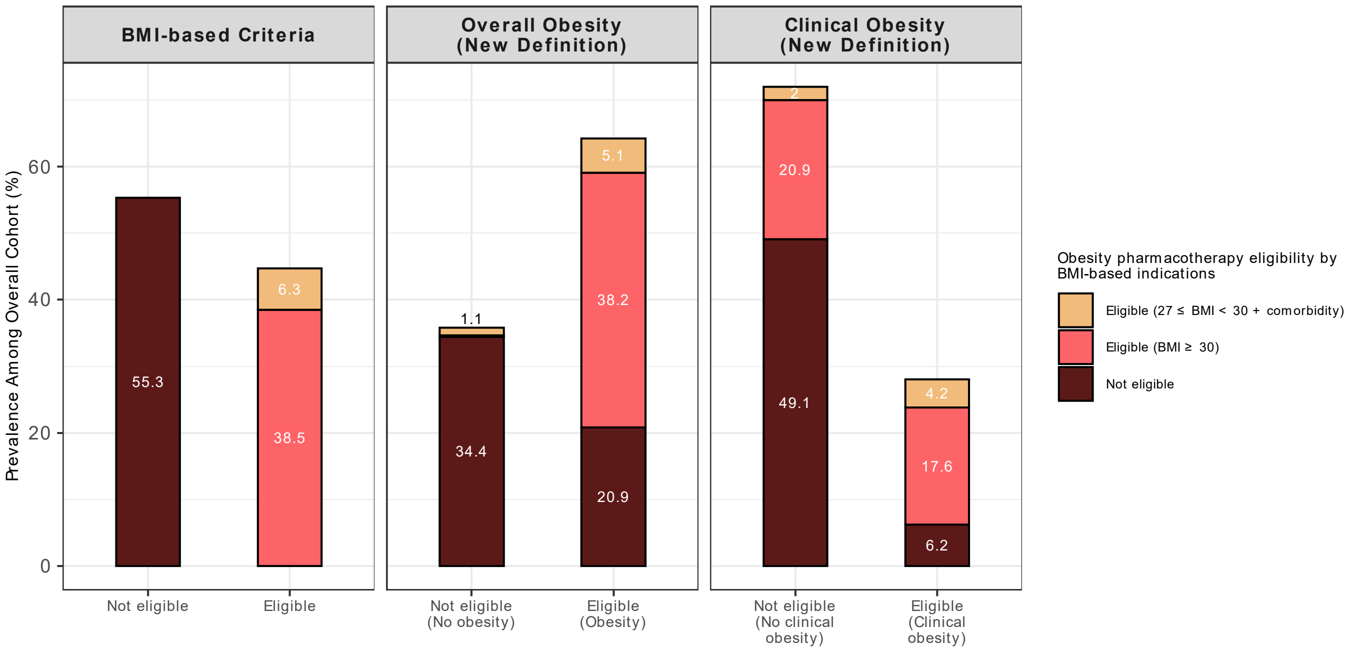

B)

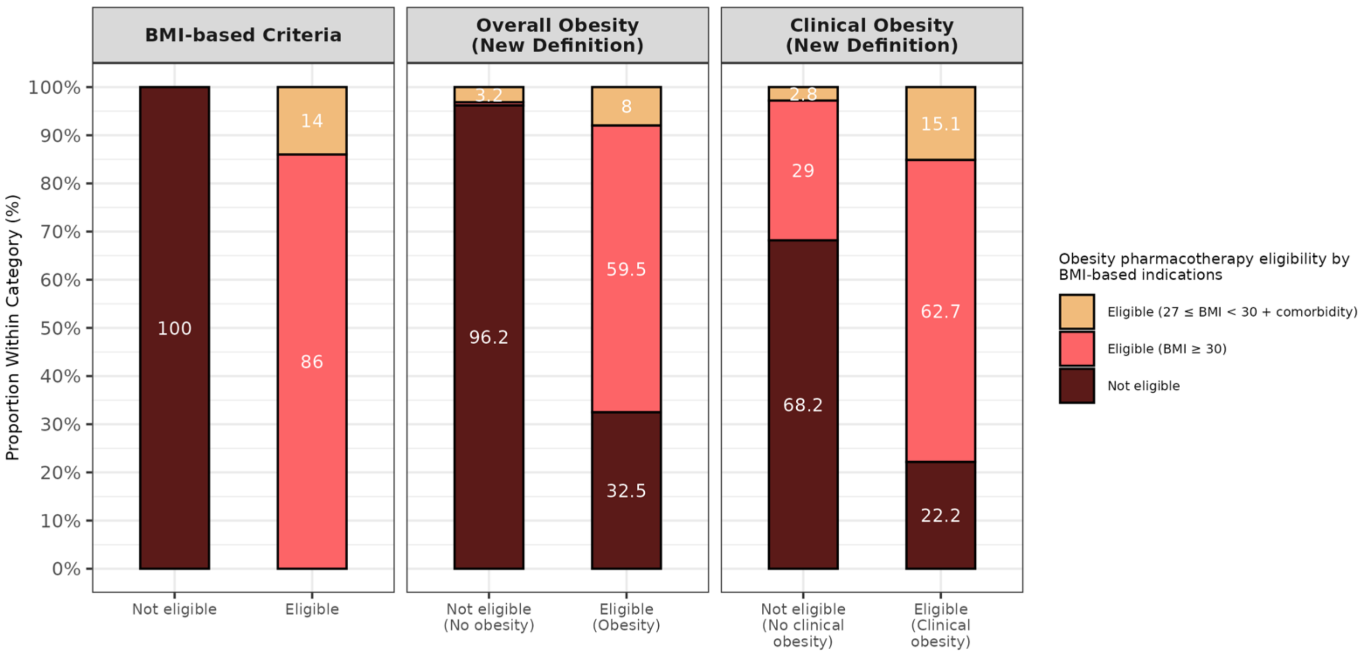

© 2025 Fourman LT et al. *JAMA Network Open*.

eFigure 16. (cont.) Obesity Pharmacotherapy Eligibility by Classification Scheme

C)

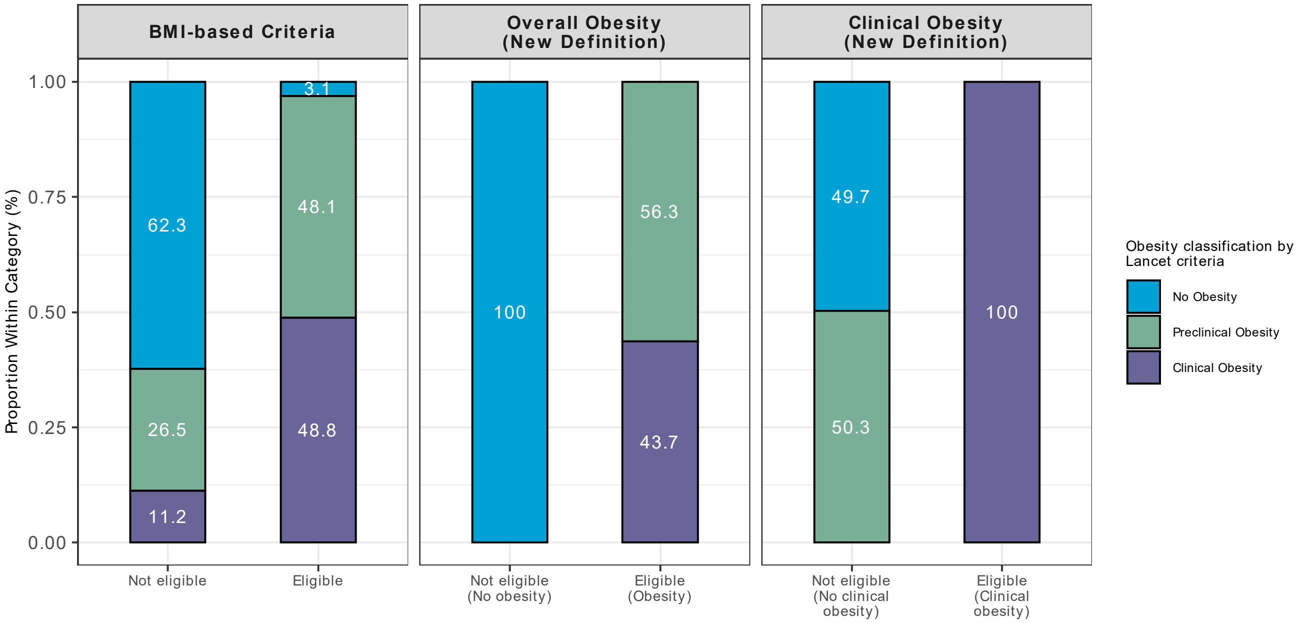

A) The prevalence of individuals meeting eligibility criteria for obesity pharmacotherapy is shown across three classification schemes: 1) BMI-based criteria, the current standard in clinical practice, which is defined as BMI ≥ 30 kg/m^2 or 27 kg/m^2 ≤ BMI < 30 kg/m^2 plus one obesity-associated comorbidity; 2) Overall obesity per the *Lancet* Commission definition; and 3) Clinical obesity per the *Lancet* Commission definition. Among individuals eligible versus not eligible for obesity pharmacotherapy by each classification scheme, the proportion meeting **B)** current BMI-based criteria and **C)** the *Lancet* Commission criteria for preclinical or clinical obesity are shown. Individuals with diabetes were excluded from this analysis as glucagon-like peptide-1 receptor agonists (GLP1RAs) are otherwise indicated in this group.

© 2025 Fourman LT et al. *JAMA Network Open*.

eFigure 17. Longitudinal Risks of Adverse Health Outcomes by Traditional vs New Obesity Definitions

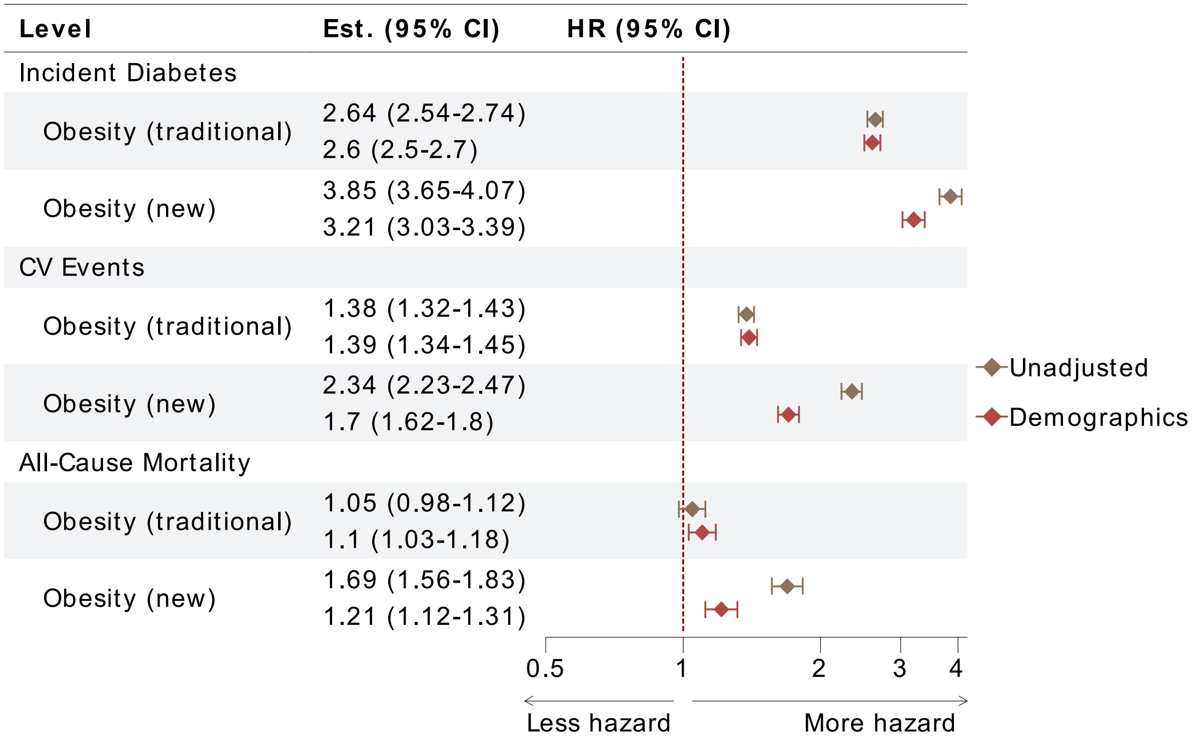

Obesity by both the traditional and new *Lancet* Commission definitions was associated with elevated risks of incident diabetes (aHR 2.60 [2.50, 2.70] vs. 3.21 [3.03, 3.39]), cardiovascular events (aHR 1.39 [1.34, 1.45] vs. 1.70 [1.62, 1.80]), and all-cause mortality (aHR 1.10 [1.03, 1.18] vs. 1.21 [1.12, 1.31]), with higher aHRs for the new definition. Forest plot displays hazard ratios with 95% confidence intervals for each longitudinal health outcome by obesity status, compared to no obesity as the reference group, per the traditional and new definitions. The unadjusted model includes only the exposure variable (obesity status). The demographics model adjusts for age, sex, and race in addition to adjusting for smoking status in analyses of cardiovascular events and all-cause mortality. *Abbreviations:* aHR, adjusted hazard ratio; CI, confidence intervals; CV, cardiovascular; Est., estimate; HR, hazard ratio.

© 2025 Fourman LT et al. *JAMA Network Open*.

eFigure 18. Longitudinal Risks of All-Cause Mortality by New Obesity Phenotype

A)

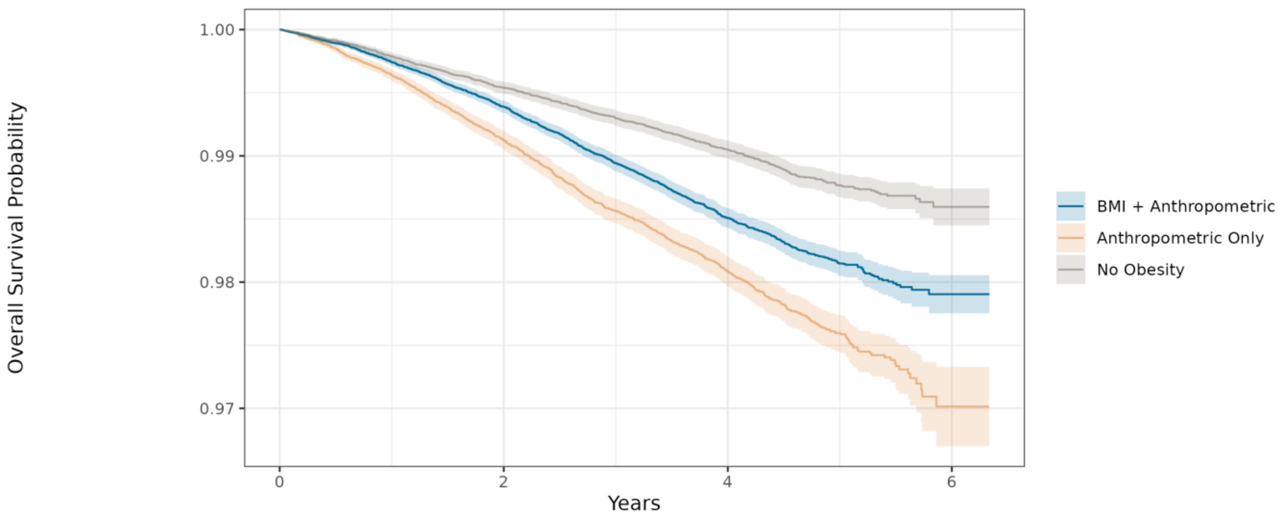

At Risk	0	2	4	6
No Obesity	94574	70269	50906	1176
Anthropometric Only	77902	54355	37647	536
BMI + Anthropometric	128129	91910	64955	967
**Events**				
No Obesity	0	371	686	798
Anthropometric Only	0	558	1072	1226
BMI + Anthropometric	0	650	1390	1568

B)

Level	HR (95% CI)
No Obesity	Ref.<br>Ref.
Anthropometric Only	2 (1.83-2.18)<br>1.2 (1.09-1.31)
BMI + Anthropometric	1.51 (1.39-1.64)<br>1.22 (1.12-1.34)

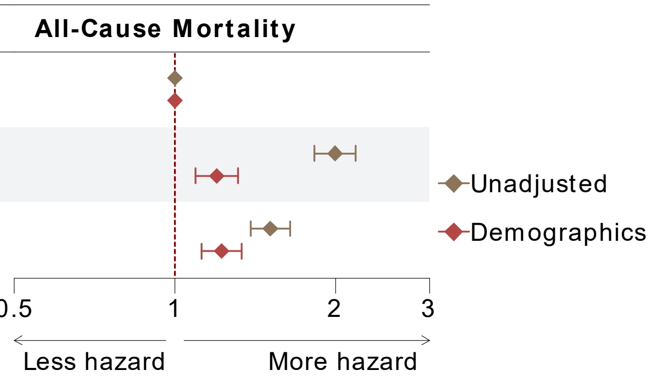

A) BMI-plus-anthropometric and anthropometric-only obesity were associated with elevated risks of all-cause mortality compared to no obesity. Kaplan-Meier curves with 95% confidence intervals (shaded) are shown for all-cause mortality by obesity phenotype. **B)** While unadjusted risk of all-cause mortality was highest among individuals with anthropometric-only obesity, adjusted risks were similar across obesity phenotypes (BMI-plus-anthropometric: aHR 1.22 [1.12, 1.34]; anthropometric-only: aHR 1.20 [1.09, 1.31]). Forest plot displays hazard ratios with 95% confidence intervals for all-cause mortality by obesity phenotype. The unadjusted model includes only the exposure variable (obesity phenotype). The demographics model adjusts for age, sex, race, and smoking status. *Abbreviations:* aHR, adjusted hazard ratio; CI, confidence intervals; HR, hazard ratio.

© 2025 Fourman LT et al. *JAMA Network Open*.

eFigure 19. Longitudinal Risks of All-Cause Mortality by Clinical Obesity Status Per the New Definition

A)

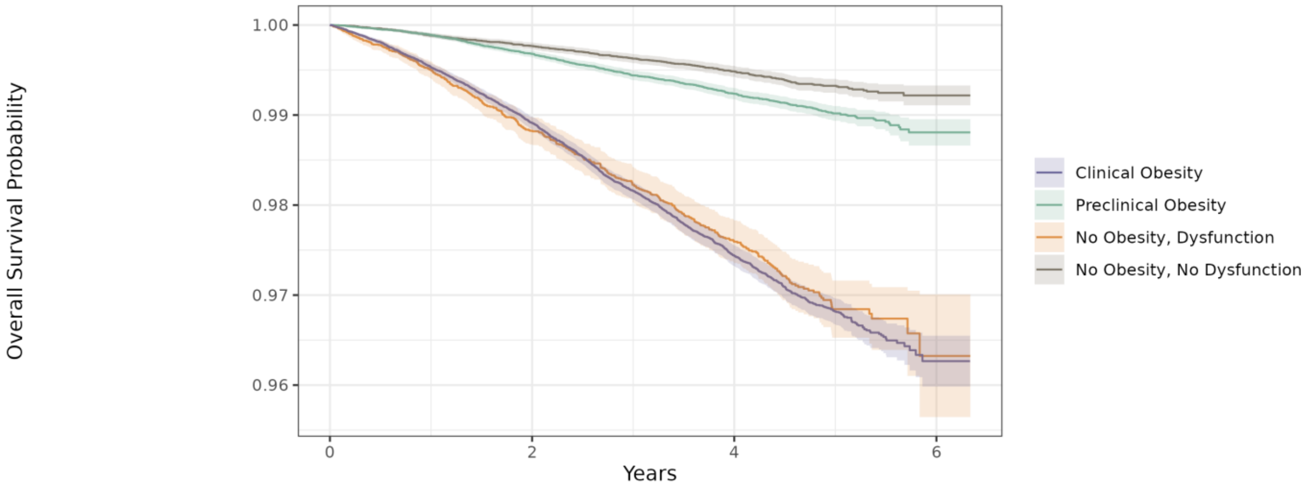

At Risk

	0	2	4	6
No Obesity, No Dysfunction	70543	54308	40273	1014
No Obesity, Dysfunction	24031	15961	10633	162
Preclinical Obesity	97632	74399	54672	903
Clinical Obesity	108399	71866	47930	600

Events

	0	2	4	6
No Obesity, No Dysfunction	0	143	283	339
No Obesity, Dysfunction	0	228	403	459
Preclinical Obesity	0	266	572	663
Clinical Obesity	0	942	1890	2131

B)

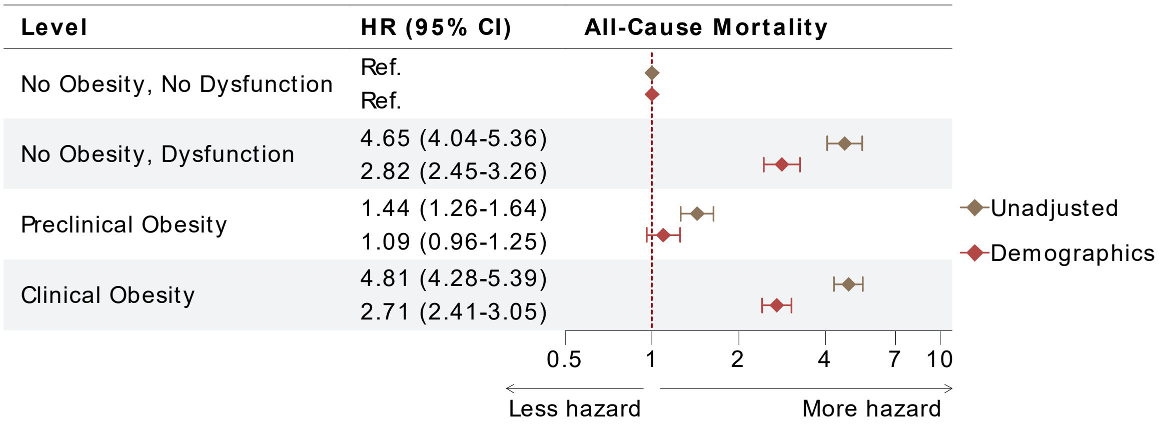

A) Individuals with clinical obesity by the new *Lancet* Commission definition and organ dysfunction in the absence of obesity exhibited elevated risks of all-cause mortality compared to those without organ dysfunction, with or without obesity. Kaplan-Meier curves with 95% confidence intervals (shaded) are shown for all-cause mortality among individuals with clinical obesity, preclinical obesity, and no obesity with or without organ dysfunction. **B)** Clinical obesity (aHR 2.71 [2.41, 3.05]) and organ dysfunction without obesity (aHR 2.82 [2.45, 3.26]) conferred similar risks of all-cause mortality, including in an adjusted model. Preclinical obesity was associated with a less marked elevation in all-cause mortality risk in unadjusted models that attenuated entirely in an adjusted model (aHR 1.09 [0.96, 1.25]). Forest plot displays hazard ratios with 95% confidence intervals for all-cause mortality among individuals with clinical obesity, preclinical obesity, and no obesity with or without organ dysfunction. The unadjusted model includes only the exposure variable (obesity and organ dysfunction status). The demographics model adjusts for age, sex, race, and smoking status. *Abbreviations:* aHR, adjusted hazard ratio; CI, confidence intervals; HR, hazard ratio.

© 2025 Fourman LT et al. *JAMA Network Open*.

eFigure 20. Associations of Clinical Obesity Status per the New Definition With Longitudinal Health Outcomes by Age Strata

A)

Age Group (Years)	Exposure	N/events	HR (95% CI)
18-29	No Obesity, No Dysfunction	18350/108	Ref.
	No Obesity, Dysfunction	1588/24	2.89 (1.86-4.51)
	Preclinical Obesity	12395/230	3.03 (2.4-3.82)
	Clinical Obesity	2600/103	7.67 (5.83-10.09)
30-39	No Obesity, No Dysfunction	14988/119	Ref.
	No Obesity, Dysfunction	2331/43	2.69 (1.9-3.81)
	Preclinical Obesity	16699/495	3.65 (2.98-4.47)
	Clinical Obesity	6057/380	9.5 (7.72-11.69)
40-49	No Obesity, No Dysfunction	10479/107	Ref.
	No Obesity, Dysfunction	2632/74	3.03 (2.26-4.08)
	Preclinical Obesity	16208/743	4.3 (3.51-5.27)
	Clinical Obesity	9596/702	8.19 (6.67-10.04)
50-59	No Obesity, No Dysfunction	10882/204	Ref.
	No Obesity, Dysfunction	4041/143	2.1 (1.7-2.61)
	Preclinical Obesity	17801/1020	3.02 (2.6-3.51)
	Clinical Obesity	15720/1494	5.97 (5.15-6.91)
60-69	No Obesity, No Dysfunction	9318/184	Ref.
	No Obesity, Dysfunction	5511/201	2.16 (1.77-2.64)
	Preclinical Obesity	16180/956	2.87 (2.45-3.37)
	Clinical Obesity	19082/1484	4.75 (4.08-5.54)
≥ 70	No Obesity, No Dysfunction	4508/99	Ref.
	No Obesity, Dysfunction	4129/144	1.94 (1.5-2.5)
	Preclinical Obesity	9353/508	2.4 (1.93-2.98)
	Clinical Obesity	15184/982	3.59 (2.91-4.42)

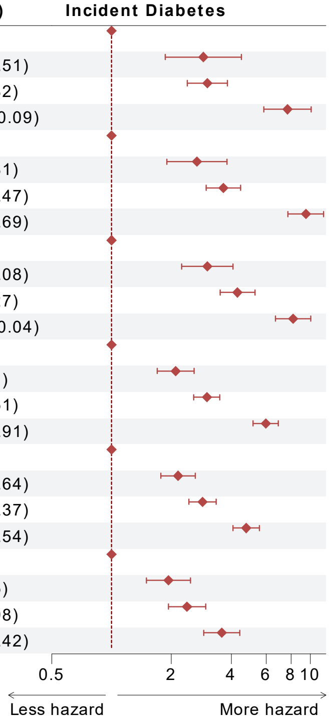

B)

Age Group (Years)	Exposure	N/events	HR (95% CI)
18-29	No Obesity, No Dysfunction	18656/31	Ref.
	No Obesity, Dysfunction	1749/20	7.24 (4.12, 12.73)
	Preclinical Obesity	12884/33	1.37 (0.83, 2.28)
	Clinical Obesity	3107/39	8.02 (4.95, 13)
30-39	No Obesity, No Dysfunction	15306/62	Ref.
	No Obesity, Dysfunction	2621/50	4.9 (3.37, 7.13)
	Preclinical Obesity	17671/100	1.22 (0.88, 1.68)
	Clinical Obesity	8066/223	6.61 (4.95, 8.82)
40-49	No Obesity, No Dysfunction	10818/72	Ref.
	No Obesity, Dysfunction	3076/116	5.87 (4.34, 7.92)
	Preclinical Obesity	17791/186	1.56 (1.18, 2.06)
	Clinical Obesity	14344/647	7.27 (5.67, 9.33)
50-59	No Obesity, No Dysfunction	11336/137	Ref.
	No Obesity, Dysfunction	4925/284	5.09 (4.13, 6.27)
	Preclinical Obesity	20084/339	1.44 (1.17, 1.76)
	Clinical Obesity	26281/1692	6.06 (5.06, 7.25)
60-69	No Obesity, No Dysfunction	9726/149	Ref.
	No Obesity, Dysfunction	6702/369	3.88 (3.2, 4.71)
	Preclinical Obesity	18511/397	1.3 (1.07, 1.57)
	Clinical Obesity	32354/2386	5.17 (4.37, 6.11)
≥ 70	No Obesity, No Dysfunction	4738/105	Ref.
	No Obesity, Dysfunction	5012/346	3.57 (2.85, 4.46)
	Preclinical Obesity	10770/293	1.14 (0.91, 1.43)
	Clinical Obesity	24498/2125	4.33 (3.54, 5.29)

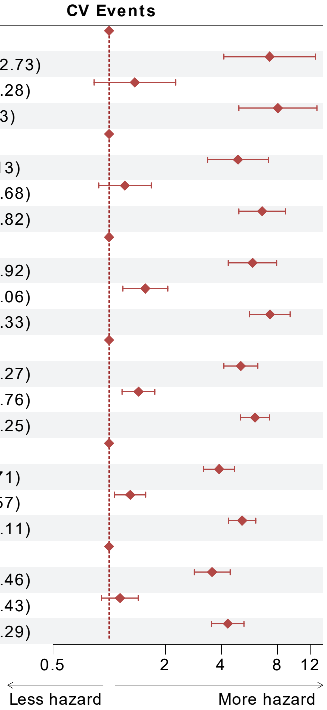

© 2025 Fourman LT et al. *JAMA Network Open*.

eFigure 20 (cont.). Associations of Clinical Obesity Status per the *Lancet* Commission Definition with Longitudinal Health Outcomes by Age Strata

C)

Age Group (Years)	Exposure	N/events	HR (95% CI)
18-29	No Obesity, No Dysfunction	18656/22	Ref.
	No Obesity, Dysfunction	1749/9	5.48 (2.36-12.75)
	Preclinical Obesity	12884/18	1.53 (0.77-3.03)
	Clinical Obesity	3107/17	5.65 (2.7-11.81)
30-39	No Obesity, No Dysfunction	15306/50	Ref.
	No Obesity, Dysfunction	2621/31	3.99 (2.43-6.55)
	Preclinical Obesity	17671/52	0.82 (0.53-1.28)
	Clinical Obesity	8066/74	3.36 (2.26-5.01)
40-49	No Obesity, No Dysfunction	10818/55	Ref.
	No Obesity, Dysfunction	3076/36	2.48 (1.59-3.87)
	Preclinical Obesity	17791/112	1.21 (0.86-1.72)
	Clinical Obesity	14344/185	2.8 (2.02-3.87)
50-59	No Obesity, No Dysfunction	11336/99	Ref.
	No Obesity, Dysfunction	4925/105	2.45 (1.83-3.27)
	Preclinical Obesity	20084/154	0.86 (0.66-1.12)
	Clinical Obesity	26281/493	2.4 (1.91-3.01)
60-69	No Obesity, No Dysfunction	9726/78	Ref.
	No Obesity, Dysfunction	6702/172	3.25 (2.45-4.29)
	Preclinical Obesity	18511/213	1.27 (0.97-1.67)
	Clinical Obesity	32354/814	3.27 (2.56-4.16)
≥ 70	No Obesity, No Dysfunction	4738/72	Ref.
	No Obesity, Dysfunction	5012/160	2.46 (1.84-3.31)
	Preclinical Obesity	10770/193	1.09 (0.82-1.46)
	Clinical Obesity	24498/799	2.3 (1.78-2.97)

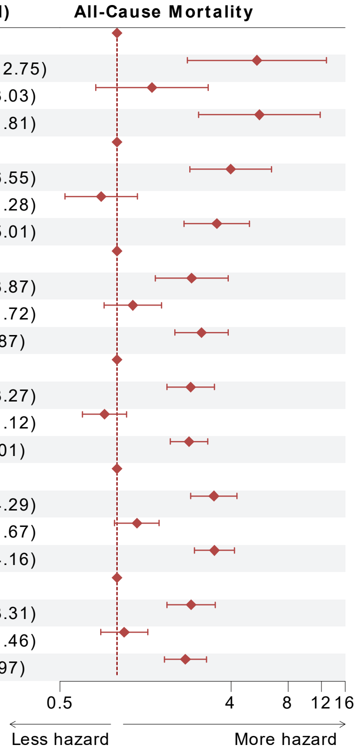

Differential risks of **A**) incident diabetes, **B)** cardiovascular events, and **C)** all-cause mortality associated with preclinical and clinical obesity by the new *Lancet* Commission definition were consistent across age strata. Forest plots display hazard ratios with 95% confidence among individuals with clinical obesity, preclinical obesity, and no obesity with or without organ dysfunction stratified by age. Models are adjusted for sex and race with additional adjustment for smoking status in analyses of cardiovascular events and all-cause mortality.

© 2025 Fourman LT et al. *JAMA Network Open*.

eReferences

1. Mayo KR, Basford MA, Carroll RJ, Dillon M, Fullen H, Leung J, et al. The All of Us Data and Research Center: Creating a Secure, Scalable, and Sustainable Ecosystem for Biomedical Research. Annu Rev Biomed Data Sci. 2023 Aug 10;6:443–64.

2. The "All of Us" Research Program. N Engl J Med. 2019 Nov 7;381(19):1883–5.

3. 30122059308052.pdf [Internet]. [cited 2025 Apr 3]. Available from: https://support.researchallofus.org/hc/en-us/article_attachments/30122059308052

4. Anderson RN, Rosenberg HM. Age standardization of death rates: implementation of the year 2000 standard. Natl Vital Stat Rep Cent Dis Control Prev Natl Cent Health Stat Natl Vital Stat Syst. 1998 Oct 7;47(3):1–16, 20.

5. Age adjustment - Health, United States [Internet]. 2024 [cited 2025 Apr 3]. Available from: https://www.cdc.gov/nchs/hus/sources-definitions/age-adjustment.htm

6. Schlueter DJ, Sulieman L, Mo H, Keaton JM, Ferrara TM, Williams A, et al. Systematic replication of smoking disease associations using survey responses and EHR data in the All of Us Research Program. J Am Med Inform Assoc. 2024 Jan 1;31(1):139–53.

© 2025 Fourman LT et al. *JAMA Network Open*.
