# Supplementary material for: Implications of a New Obesity Definition Among the All of Us Cohort
Source: JAMA Netw Open. 2025 Oct 15;8(10):e2537619. doi: 10.1001/jamanetworkopen.2025.37619 (PMC12529213; doi:10.1001/jamanetworkopen.2025.37619)
Supplement: Supplement 2. — Data Sharing Statement [file jamanetwopen-e2537619-s002.pdf]

Data Sharing Statement

Fourman. Implications of a New Obesity Definition Among the All of Us Cohort. *JAMA Netw Open*. Published October 15, 2025. doi:10.1001/jamanetworkopen.2025.37619

Data
Data available: No

Additional Information
Explanation for why data not available: The All of Us data is available to approved researchers following registration, completion of ethics training, and attestation of a data use agreement through the All of Us Research Workbench platform that is accessible via https://workbench.researchallofus.org/.
